# Bioinformatics Analysis of Global Proteomic and Phosphoproteomic Data Sets Revealed Activation of NEK2 and AURKA in Cancers

**DOI:** 10.3390/biom10020237

**Published:** 2020-02-04

**Authors:** Barnali Deb, Pratyay Sengupta, Janani Sambath, Prashant Kumar

**Affiliations:** 1 Institute of Bioinformatics, International Technology Park, Bangalore 560066, India; barnali@ibioinformatics.org (B.D.); janani@ibioinformatics.org (J.S.); 2 Manipal Academy of Higher Education (MAHE), Manipal 576104, India; 3 Department of Biotechnology, National Institute of Technology Durgapur, Mahatma Gandhi Avenue, Durgapur, West Bengal 713209, India; pratyaysengupta1628@gmail.com

**Keywords:** clear cell renal cell carcinoma, lung adenocarcinoma, uterine corpus endometrial carcinoma, phosphorylation, CPTAC, drug targets

## Abstract

Tumor heterogeneity attributes substantial challenges in determining the treatment regimen. Along with the conventional treatment, such as chemotherapy and radiotherapy, targeted therapy has greater impact in cancer management. Owing to the recent advancements in proteomics, we aimed to mine and re-interrogate the Clinical Proteomic Tumor Analysis Consortium (CPTAC) data sets which contain deep scale, mass spectrometry (MS)-based proteomic and phosphoproteomic data sets conducted on human tumor samples. Quantitative proteomic and phosphoproteomic data sets of tumor samples were explored and downloaded from the CPTAC database for six different cancers types (breast cancer, clear cell renal cell carcinoma (CCRCC), colon cancer, lung adenocarcinoma (LUAD), ovarian cancer, and uterine corpus endometrial carcinoma (UCEC)). We identified 880 phosphopeptide signatures for differentially regulated phosphorylation sites across five cancer types (breast cancer, colon cancer, LUAD, ovarian cancer, and UCEC). We identified the cell cycle to be aberrantly activated across these cancers. The correlation of proteomic and phosphoproteomic data sets identified changes in the phosphorylation of 12 kinases with unchanged expression levels. We further investigated phosphopeptide signature across five cancer types which led to the prediction of aurora kinase A (AURKA) and kinases-serine/threonine-protein kinase Nek2 (NEK2) as the most activated kinases targets. The drug designed for these kinases could be repurposed for treatment across cancer types.

## 1. Introduction

Aberrant post-translational modifications such as phosphorylation may drive numerous fundamental biological processes which may lead to tumor initiation and progression [1]. It is primarily carried out by the dysregulated phosphorylation of the signaling intermediates by altered kinase activity. Therefore, employment of advanced methods would identify activated signaling pathways and potential drug targets to delineate personalized therapeutic intervention strategies for improved treatment outcomes. Mass spectrometry has boomed as the preferred choice of technology for identifying and quantifying phosphorylation events which could reveal the dysregulated kinase activity in diseased conditions [2]. Over the last decade, multiple kinase-targeted drugs, including small-molecule inhibitors and antibodies, have been approved by FDA for clinical use in cancer treatment [3]. Kinases are the second most common class of protein drug targets for cancer treatment and have shown significant favorable treatment outcome compared to conventional cytotoxic therapy (for example, imatinib and dasatinib) [4,5]. However, the discovery of a new drug against cancer is a challenging process in terms of cost and duration, with a low probability to enter the clinical trials. Therefore, drug repurposing or use of a single therapeutic drug across cancer types would be beneficial for speeding up the progress towards cancer treatment.

Numerous mass spectrometry-based phosphoproteomic data sets have been analyzed to identify the phosphorylation levels in several cancers including breast cancer [6], lung cancer, ovarian cancer [7], hepatocellular carcinoma [8], prostate cancer [9], etc. Efforts have also been laid to identify the altered signaling pathways in cancer such as in bladder carcinoma [10]. However, much attention has not been paid to the clinical applications of these large datasets. Thus, re-analysis and deeper interpretation of proteomic data sets which are available publically are best suited for such studies where common treatment approaches could be designed as targeted therapy [11].

Clinical Proteome Tumor Analysis Consortium (CPTAC) is the data portal that is a centralized repository for the public dissemination of proteomic sequence datasets. In this study, we have integrated proteomic and phosphoproteomic data sets of breast cancer, clear cell renal cell carcinoma (CCRCC), colon cancer, lung adenocarcinoma (LUAD), ovarian cancer, and uterine corpus endometrial carcinoma (UCEC) from the CPTAC data portal. We sought to identify a common kinase target across cancers. Our study offered a comprehensive global phosphoproteomic data analysis that could be utilized for better clinical outcomes aided through recommendation of kinase inhibition across cancer types.

## 2. Materials and Methods

### 2.1. Phosphoproteomic and Proteomic Data Mining

**Figure 1:**
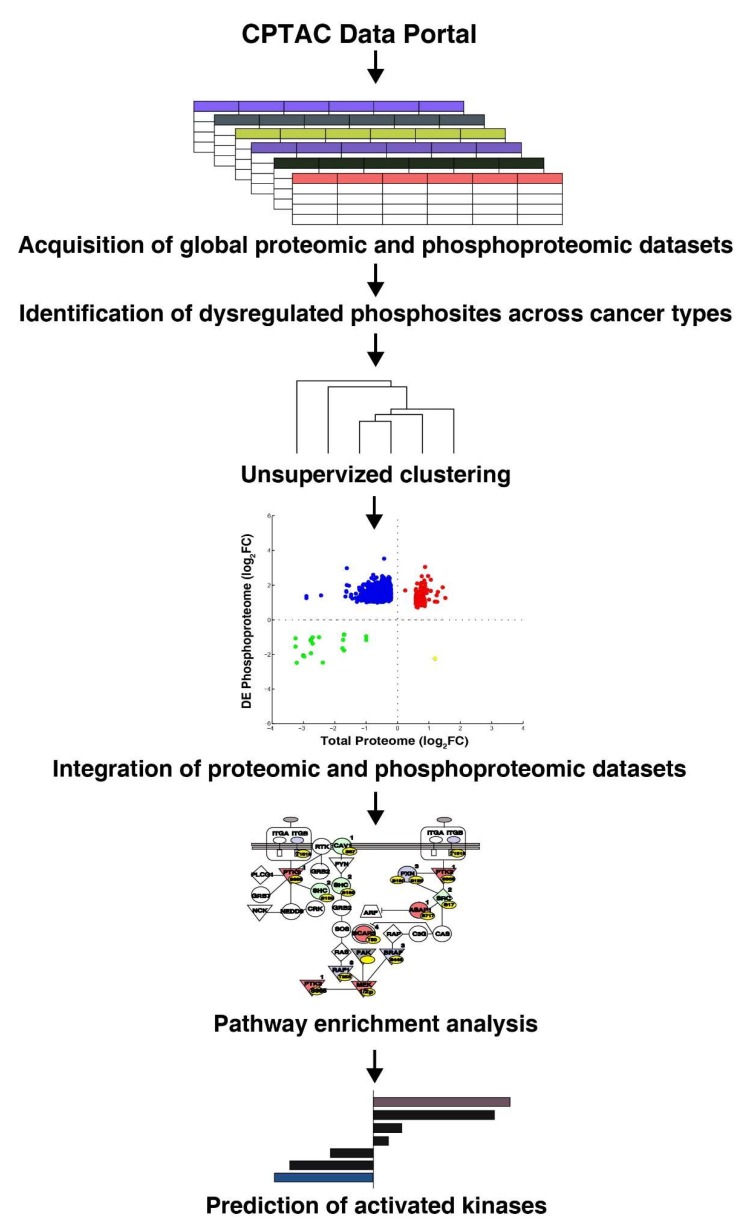
Workflow depicting re-interpretation of quantitative global and phosphoproteomic data sets acquired from the CPTAC database for six cancer types (breast cancer, clear cell renal cell carcinoma (CCRCC), colon cancer, lung adenocarcinoma (LUAD), ovarian cancer, and uterine corpus endometrial carcinoma (UCEC)).

Data used in this publication were generated by the Clinical Proteomic Tumor Analysis Consortium (NCI/NIH). The quantitative phosphoproteomic and global proteomic data sets for six cancer types including breast cancer, clear cell renal cell carcinoma (CCRCC), colon cancer, lung adenocarcinoma (LUAD), ovarian cancer, and uterine corpus endometrial carcinoma (UCEC) were downloaded (https://cptac-data-portal.georgetown.edu/cptacPublic/). The acquisition of data included 10-plex TMT enrichment protocol for the quantitation and was analyzed through the CPTAC Common Data Analysis Pipeline (CDAP). The LC-MS/MS-based analyzed datasets were downloaded (in tsv file format; phosphopeptide relative quantitation report). A 1.5-fold cut-off was used to identify the dysregulated phosphopeptides. The detailed workflow is described in Figure 1.

### 2.2. Clustering of Phosphoproteomic Data Sets

All quantified peptides (across six cancers namely breast cancer, CCRCC, colon cancer, LUAD, ovarian cancer, and UCEC were considered to identify differentially phosphorylated peptides. The median value of the fold changes of total samples for each phosphosite was considered for complete unsupervised clustering using MORPHEUS (https://software.broadinstitute.org/morpheus/). Principal component analysis (PCA) was performed using R-packages v.3.6.0 (http://www.R-project.org/).

### 2.3. Kinome Map

The kinome map was built using the KinMap online tool (http://www.kinhub.org/kinmap/index.html). The lists of identified kinases were searched and were highlighted on the kinome map.

### 2.4. Pathway Analysis

Pathway analysis was performed for dysregulated phosphopeptide signature across breast cancer, colon cancer, LUAD, ovarian cancer, and UCEC using the Reactome database (https://reactome.org/). Reactome is an open access, open source, manually curated, peer-reviewed pathway database of human pathways and processes. A false discovery rate (FDR) corrected *p* value < 0.05 cut-off was set and the list of altered signaling pathways were identified.

### 2.5. Protein–Protein Interaction Network Analysis

Interaction network was analyzed using the STRING functional protein association network (https://string-db.org; version: 11.0; University of Zurich, Zurich, Switzerland) [12]. The input was the set of dysregulated phosphopeptide signature across breast cancer, colon cancer, LUAD, ovarian cancer, and UCEC and was set to highest confidence (0.90) of active interaction. The disconnected nodes were hidden, and K-means clustering was conducted to identify three clusters in the data set.

### 2.6. Quadrant Plot for Comparative Expression and Phosphorylation Levels of Proteins

The quadrant plot for each cancer was plotted taking logarithmic fold change values of the total proteomics in the x-axis and corresponding differentially expressed phosphorylation data in the y-axis to represent their comparative regulation. MATLAB v.R2014a was used to perform these plots.

### 2.7. Prediction of Activated Kinases Using Kinase-Substrate Enrichment Analysis (KSEA) Tool and Overall Survival Estimates

Kinase-substrate enrichment analysis was done using the online KSEA tool (https://casecpb.shinyapps.io/ksea/). Phosphopeptide signature dysregulated across five cancer types was used for the input and analyzed using PhosphoSite Plus and NetworKIN as the background data sets. The p-value cut-off (for plot) and number of substrates cut-off were set to 0.05 and 10, respectively.

The survival plots for the enriched kinases through KSEA were plotted using Kaplan–Meier plotter; KMplotter (https://kmplot.com/analysis/) [13].

### 2.8. Motif Analysis

The enriched motifs in common phosphopeptides were identified using the MoMo tool (http://meme-suite.org/tools/momo) which re-implemented the Motif-X and MoDL algorithm. Phosphopeptide window of 13 amino acids were used for consensus motif search with serine and threonine as central residues. The minimum number of occurrences for a motif in the data set was set to 15 and 10 for pSer and pThr peptides, respectively with a required motif significance of 10 × 10^−6^.

## 3. Results

### 3.1. Dysregulation of Protein Phosphorylation in Cancer Types

The phosphoproteomic data sets were downloaded from the CPTAC data portal (https://cptac-data-portal.georgetown.edu/cptacPublic/). The details of the data sets used in this study are provided in Table 1.

**Figure 2:**
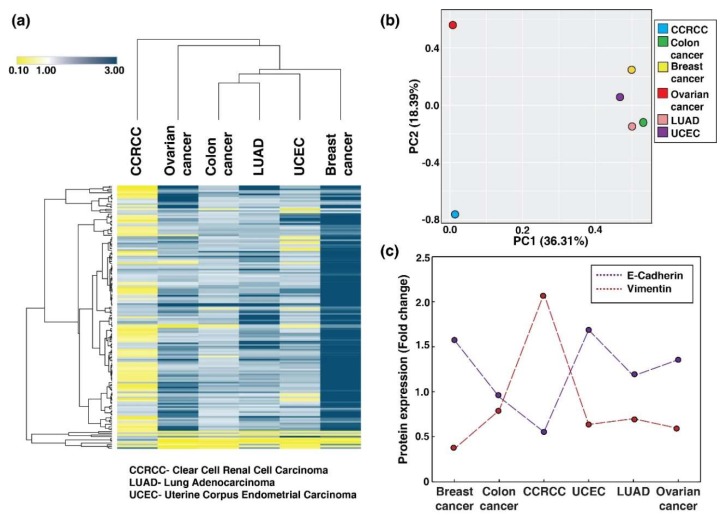
Dysregulation of protein phosphorylation and epithelial-mesenchymal transition (EMT) expression levels in cancers. (**a**) Unsupervised clustering of dysregulated phosphosites across six cancer types using Morpheus. (**b**) Principle component analysis of dysregulated phosphosites across six cancer types. (**c**) Scatter plot showing the expression of EMT markers (E-Cadherin and Vimentin) across six cancer types.

Dysregulated phosphosites (1.5-fold) across the six cancer types were used for an unsupervised clustering. One hundred and sixty-one phosphosites were commonly dysregulated across six cancer types (Table S1). Clustering shows that breast cancer, colon cancer, LUAD, ovarian cancer, and UCEC forms one cluster, however, CCRCC is a distinct offset branch with decreased phosphorylation of phosphosites as compared to the other five cancers (83 phosphosites are hypophosphorylated) (Figure 2a). Principle component analysis plotted using first and second component with 54.7% of variability represents the percentage of variance. It further confirms the clustering pattern of dysregulated phosphopeptide signature (Figure 2b).

### 3.2. Epithelial-Mesenchymal Transition (EMT) and Its Molecular Regulation Across Six Cancer Types

The epithelial and mesenchymal characteristics of each cancer type are controlled at various levels of molecular regulation which could lead to differences in expression or post-translational modifications. Hence, we also checked the protein expression for the markers of EMT such as E-cadherin 1 (CDH1) and Vimentin (VIM). We observed that CCRCC shows mesenchymal characteristics with high VIM and low CDH1 expression unlike other cancer types which reflects epithelial characteristics with high CDH1 and low VIM expressions (Figure 2c).

### 3.3. A Common Phosphorylation Signature Identified

**Figure 3:**
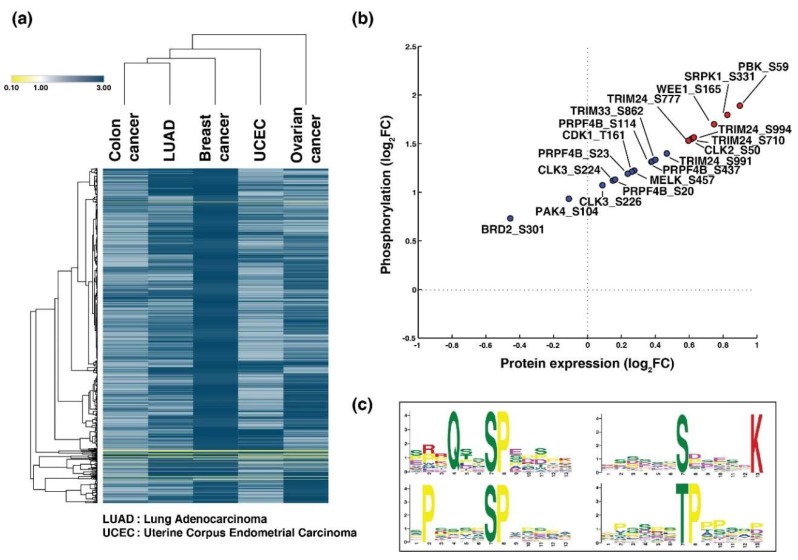
Unique phosphorylation signature across five cancers. (**a**) Unsupervised clustering of 880 phosphopeptide signature across five cancer types using Morpheus. (**b**) Scatter plot of the hyperphosphorylated kinases (y-axis) identified in the study and their corresponding protein expression (x-axis). (**c**) Motifs enriched in the phosphopeptide signature across five cancer types.

A phosphorylation pattern (residues that are phosphorylated across the cancer types following a common regulation pattern, i.e., hyperphosphorylated or hypophosphorylated (1.5-fold) across the cancer types may suggest similar biological processes contributing to tumor characteristics. Since CCRCC was identified as a distinct cluster and showed typical mesenchymal characteristics unlike the other cancer types, it was not considered for identifying the phosphorylation signature. We identified 880 phosphorylation sites that had common phosphorylation patterns across the five the cancer types (breast cancer, colon cancer, LUAD, ovarian cancer, and UCEC) (Table S2). Unsupervised clustering depicts the signature of 880 phosphorylation sites corresponding to 514 proteins (Figure 3a). Seven hundred and sixty-four serine, one hundred and ten threonine, and six tyrosine sites were identified to comprise the signature.

### 3.4. Unique Phosphorylation of Proteins Identified Through the Integration of Global Protein Expression of the Phosphopeptide Signature

Altered expression levels (overexpression or downregulation) of proteins may contribute to altered phosphorylation pattern (hyperphosphorylation or hypophosphorylation). To identify the unique phosphorylation of the proteins, which are not due to its altered expression levels, we checked the protein expression from the global proteomic data sets in the CPTAC database of the same samples across the five cancer types. 8540, 7418, 11,029, 8818, and 10,768 proteins were identified in breast cancer, colon cancer, LUAD, ovarian cancer, and UCEC, respectively. The percentage of phosphosites identified across five cancer types is depicted in Figure S1. Breast cancer, colon cancer, LUAD, ovarian cancer, and UCEC were identified to have 535, 714, 801, 785, 757 dysregulated phosphosites, respectively whereas the corresponding protein expression was observed to be unchanged or down-regulated (Supplementary Materials Figure S2).

### 3.5. Hyperphosphorylated Kinases with Basal Level Expression Were Identified

A total of 12 kinases were identified to be differentially phosphorylated across the five cancer types (Figure S3). Of these, five were CMGC (CDK, MAPK, GSK3, and CLK set of families), one each were TLK (tyrosine-like kinases), STE (homologs of yeast Sterile 7, Sterile 11, and Sterile 20 kinases), CK1 (casein kinases) and CAMK (calmodulin/calcium regulated kinases), and three atypical kinases. BRD2 (S301), PAK4 (S104), CLK3 (S226), CLK3 (S224), PRPF4B (S20), PRPF4B (S23), CDK1 (T161), MELK (S457), PRPF4B (S144), PRPF4B (S437), TRIM33 (S862), and TRIM24 (S991) were observed to be hyperphosphorylated, however, their expression levels were unchanged or were downregulated (Figure 3b).

### 3.6. Proline-Directed Motifs Were Highly Phosphorylated Across Five Cancer Types

Among the five cancer types, “proline-directed motifs” were enriched among both the serine and threonine phosphorylated peptides. Six serine phosphorylated motifs and one threonine phosphorylated motif were identified using the MoMo tool. The consensus motifs “QxxSP”, “PxxxxSP”, “SxxxxxK”, and “TP” were observed to be highly enriched (Figure 3c).

### 3.7. Cell Cycle Pathway Was Enriched Across the Five Cancer Types

**Figure 4:**
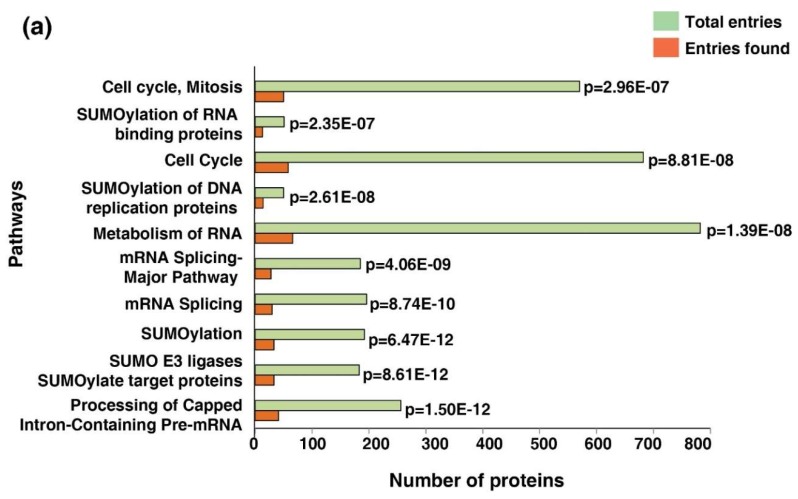

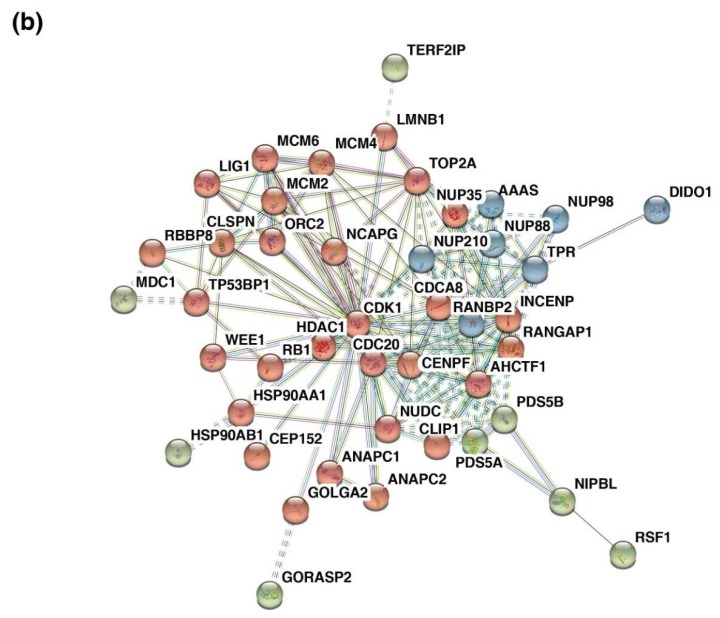
Enriched dysregulated pathways and interaction clusters across five cancer types. (**a**) Bar graph of the top enriched pathways across five cancer types identified using the Reactome pathway analysis tool. (**b**) Protein–protein interaction network showing the protein clusters involved in the cell cycle pathway with highest confidence (0.90) acquired using the STRING functional protein association network tool.

Considering the signature 514 proteins, a pathway analysis was conducted using the Reactome pathway database-analysis tool. Ten most enriched pathways (Figure 4a). The cell cycle pathway was one of the most enriched pathways across the five cancer types (*p* = 8.81 × 10^−8^; FDR = 1.02 × 10^−5^). Forty-eight proteins were enriched in the cell cycle pathway. Metabolism of the RNA pathway was among the other key pathways dysregulated across cancer types (*p* = 1.39 × 10^−8^; FDR = 1.08 × 10^−4^). The dysregulated phosphoproteins involved in the cell cycle pathway are listed in Table S3.

### 3.8. Protein Interaction Clusters Common across Five Cancers

The 48 proteins that were enriched in the cell cycle pathway were used for the network analysis (Figure S4). The network revealed two major clusters with CDK1 (Cyclin-dependent kinase 1) and RANBP2 (RAN Binding Protein 2).

CDK1 was observed to be the key hub proteins that interacted with LMNB1 (Lamin-B1), ANAPC1 and C2 (Anaphase-promoting complex subunit 1 and 2), CEP152 (Centrosomal protein of 152 kDa), HSP90AA1 (Heat shock protein HSP 90-alpha), HDAC1 (Histone deacetylase 1), MCM2,4,6 (Minichromosome Maintenance Complex Component 2, 4, and 6), RB1 (Retinoblastoma-associated protein), ORC2 (Origin recognition complex subunit 2), NCAPG (Non-SMC Condensin I Complex Subunit G), GOLGA2 (Golgin A2), WEE1 (Wee1-like protein kinase), CDC20 (Cell division cycle protein 20 homolog), PDS5A/B (Sister Chromatid Cohesion Protein PDS5 Homolog A and B), CLIP1 (CAP-Gly domain-containing linker protein 1), NUDC (Nuclear migration protein nudC), CENPF (Centromere protein F), TOP2A (DNA topoisomerase 2-alpha), CDCA8 (Cell Division Cycle Associated 8), and INCENP (Inner centromere protein).

RANBP2 interacted with AAAS (Aladin WD Repeat Nucleoporin), NUP35/88/98 (Nuclear pore complex protein Nup35, Nup88, and Nup98), TPR (Nucleoprotein TPR), RANGAP1 (Ran GTPase-activating protein 1), NUP210 (Nuclear pore membrane glycoprotein 210), and AHCTF1 (AT-Hook Containing Transcription Factor 1) (Figure 4b).

### 3.9. Serine/Threonine-Protein Kinase Nek2 (NEK2) and Aurora Kinase A (AURKA) Are the Most Predicted Activated Kinases across the Five Cancers

**Figure 5:**
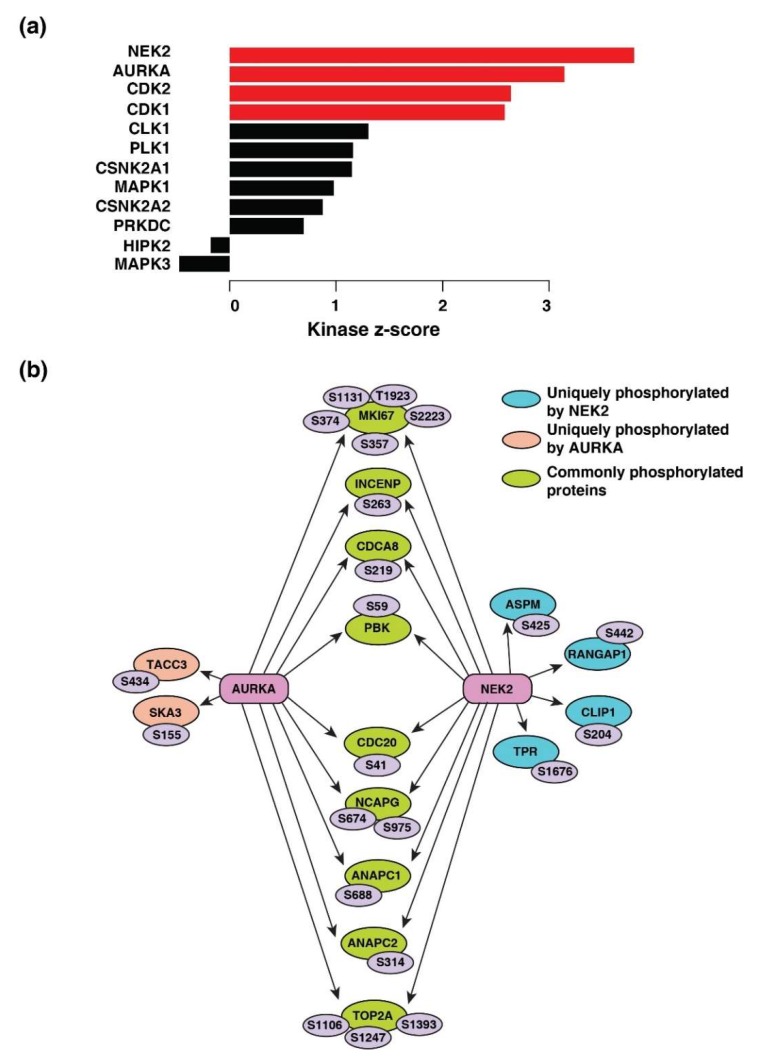
Kinase-substrate enrichment analysis. (**a**) Predicted upstream kinases enriched across five cancer types. Graph showing the positively regulated upstream kinases (red bars) predicted to be activated. (**b**) Substrates of kinases-serine/threonine-protein kinase Nek2 (NEK2) and aurora kinase A (AURKA) enriched across cancer types depicted by a schematic diagram. The respective phosphosites of the substrates identified are also highlighted.

Four kinases-serine/threonine-protein kinase Nek2 (NEK2), aurora kinase A (AURKA), cyclin-dependent kinase 1 and 2 (CDK1 and CDK2) were the predicted to be activated across breast cancer, colon cancer, LUAD, ovarian cancer, and UCEC (Figure 5a). The kinase-substrate links and the respective kinase scores are provided in Tables S4 and S5, respectively. NEK2 (z-score = 3.79; *p* = 7.34 × 10^−5^) and AURKA (z-score = 3.14; *p* = 0.0008) were predicted to be most activated and responsible for the phosphorylation of 20 and 18 downstream proteins, respectively (Figure 5b).

## 4. Discussion

Most of the oncogenic transformations in the cells are initiated primarily through mutation in the genes including the kinase coding genes. Mutations in these kinases often lead to the constitutive kinase activity followed by cellular aberrations and tumorigenesis. Thus, an extensive research is striding to identify the inhibitors for the kinases with increased activity for better clinical outcome and cancer management. Several families of kinases, such as tyrosine kinases [14], cycle-dependent kinases [15,16,17], aurora kinases [17,18], mTOR [19], and mitogen-activated protein kinases [20] already have FDA approved inhibitors, which are at different phases of clinical trials. Large profiling data sets have proved to be immensely useful for translational research in the area of cancer treatment. However, only recent efforts are being embraced for providing more repurposing opportunities for the kinase inhibitors. In this study, we have re-analyzed and integrated the global proteomic and phosphoproteomic data sets from six cancer types, namely, breast cancer, CCRCC, colon cancer, LUAD, ovarian cancer, and UCEC from the CPTAC data portal. With a combined integrative and bioinformatics approach, we identified the dysregulation in the cell cycle pathway and predicted the activation of NEK2 and AURKA across breast cancer, colon cancer, LUAD, ovarian cancer, and UCEC.

We identified CCRCC as a distinct cluster which showed a discrete phosphorylation pattern. CCRCC has been reported to have a strong propensity to metastasize and more than 30% cases are metastatic at diagnosis [21]. We observed that CCRCC tumors exhibit mesenchymal characteristics owing to the fact that they have a high VIM and low CDH1 expression pattern. The other cancer types (breast cancer, colon cancer, LUAD, ovarian cancer, and UCEC) were found to have the inverse expression pattern suggesting more epithelial characteristics. CCRCC has also previously reported to have an increased generic EMT transcript score (EMT score ranges from −1 to 1; increase in the positive value represents mesenchymal phenotype and vice versa) which reflects the mesenchymal characteristics [22]. Nevertheless, carcinoma cells can adopt several intermediate stages of transition, perhaps metastable stages; however that is yet unexplored [23]. Combination of these and many more factors could contribute to its more metastatic nature, as well as its distinct pattern of phosphorylation. Hence, CCRCC being dissimilar to the other cancer types was not considered further for the analysis to identify potential activated kinases in cancer types.

Commonly dysregulated phosphosites across the five cancer types were used for pathway annotation and led to the identification of the cell cycle pathway to be differentially regulated. Cell cycle is one of the most commonly reported activated pathways in cancer since decades [24,25,26,27,28,29]. A few kinases such as the cyclin dependent kinases (CDKs), checkpoint proteins, and other regulatory transcription factors synchronize and maintain the progress of cells through their division phase. Its role is defined to be very critical in tumor development, as the transforming cells depend on specific cell cycle proteins to inhibit tumor-suppressive programs such as senescence and apoptosis. This process selectively sensitizes cancer cells to inhibition of these proteins [30]. This eventually leads to replication and division of cells. The key player of cell cycle regulation is CDK1 that we identified to be phosphorylated on T161 which is known to be regulated by the CDK-activating kinase (CAK) [31]. Constitutive activation of CDK1 during mitotic cell division is achieved through the phosphorylation of T161. During interphase, the activity of CDK1 is known to be restricted by inhibitory phosphorylations in the active site on Y15 and to a lesser extent on T14 [32]. Moreover, we also identified proline-directed motifs to be highly enriched across the five cancer types which also suggested cells being highly proliferative and present in the mitotic stage of the cell cycle [33]. We also identified the dysregulation of RNA metabolism pathway across five cancer types. The altered metabolic features are observed generally across cancer types, thus, reprogrammed metabolism is considered to be a hallmark of cancer. The classical example of a reprogrammed metabolic pathway in cancer is the Warburg effect or aerobic glycolysis [34,35]. Selective inhibition of these kinases presents a potential attractive strategy to cancer therapy, proposing that a therapeutic window could be achieved.

Moreover, we tried to investigate the potential kinases that could be targeted therapeutically across the five cancer types. We identified that the NEK2 and AURKA kinase were predicted to be potentially activated. Although not identified in each of the five cancer type datasets, yet the enrichment of significant number of downstream target substrates assures probable activation of NEK2 and AURKA across the five cancers. NEK2 is one of the critical players in mitotic cell cycle. It is involved in centrosome duplication and separation, microtubule stabilization, kinetochore attachment, and spindle assembly checkpoint [36]. The activation of NEK2 is through the phosphorylation on its auto-phosphorylation sites such as (T170/S171 and T175) [37]. Carboxamide 11 is one of the best known selective inhibitors of NEK2 [38]. NEK2 has been reported to have a role in the progression of several malignancies, importantly in breast cancer, therefore devising the potential of an anticancer therapy [36,39,40]. AURKA is yet another essential target for a better clinical outcome in cancer treatment. It is reported to be overexpressed in numerous cancers such as breast cancer [41], ovarian cancer [42,43], colorectal adenoma [44], colon cancer [45] gastrointestinal cancer [46], and lung cancer [47], and is associated with the poor prognosis. Moreover, we observed that high grade breast cancer patients and lung adenocarcinoma patients with a high *AURKA* and *NEK2* gene expression had a significant poor overall survival. However, UCEC patients displayed a poor overall survival only for *AURKA* gene expression (Figure S5). Alisertib; a second-generation, highly selective small molecule inhibitor of AURKA has also been considered for clinical trials (NCT01045421). CDK1/2 were also identified to be activated across five cancer types. However, CDK4/6 inhibition (Abemaciclib) have been reported widely in several cancers such as Neuroblastoma Ewing sarcoma, Rhabdomyosarcoma, Osteosarcoma. Recently, CKD1/2/4/6/7 inhibitor (Flavopiridol) are in clinical trials for several solid tumors, as well as lymphoma (NCT00012181). Klaeger et al. has intricately studied the role of 243 kinase inhibitors and the benefits of repurposing them [48]. Although the potential of kinase inhibitors have been known for decades, extensive research on this area has started gaining momentum recently. Nonetheless, these predictions need to be observed explicitly through clinical validations and implemented rendering in cancer therapy and treatment.

## 5. Conclusions

This is a comprehensive re-analysis of large proteomic data sets obtained through CPTAC, an open access resource. This study highlights the imminent importance of re-interrogation and integration of publicly available “big data”. Our study provides the insights into the altered cell cycle signaling pathway and predicts NEK2 and AURKA kinases to be activated in breast cancer, colon cancer, LUAD, ovarian cancer, and UCEC. Thus, these could serve as a potential therapeutic target across these five cancers.

## Figures and Tables

**Table 1:** Details of the data sets of six cancer types downloaded from the CPTAC data portal.

Study Details	CPTAC Cancer Proteome Confirmatory Colon Study	CPTAC Ovarian Cancer Confirmatory Study	CPTAC Breast Cancer Confirmatory Study	CPTAC Uterine Corpus Endometrial Carcinoma (UCEC) Discovery Study	CPTAC Clear Cell Renal Cell Carcinoma (CCRCC) Discovery Study	CPTAC Lung Adenocarcinoma (LUAD) Discovery Study
**CPTAC Accession Number**	S037	S038	S039	S043	S044	S046
**Tumor Sample Count**	97	84	133	100	110	113
**Adjacent Normal Sample Count**	100	19	18	40	84	102
**Unique Phosphosites Identified**	40,302	43,811	65,068	43,842	41,809	45,671
**Unique Protein Identified**	4724	5299	5852	6155	5740	6020

## Supplementary Materials

The following are available online at https://www.mdpi.com/2218-273X/10/2/237/s1. Figure S1: The percentage of phosphosites identified across five cancer types (breast cancer, colon cancer, LUAD, ovarian cancer, and UCEC). Grey, green, and blue represent the percentage of serine, threonine, and tyrosine sites respectively; Figure S2: Quadrant plot representing the dysregulated phosphopeptides across five cancer types and their corresponding protein expressions; Figure S3: Kinome map depicting the identified kinases in the data set. Kinases are highlighted as in the inset legend. The map was built using the KinMap tool. Illustration reproduced courtesy of Cell Signaling Technology, Inc. (www.cellsignal.com); Figure S4: Protein–protein interaction network showing the interaction among 48 proteins involved in the cell cycle pathway with highest confidence (0.90) acquired using STRING functional protein association network tool; Figure S5: Kaplan–Meier plot for (a) breast cancer, (b) LUAD, and (c) UCEC patients stratified according to high and low expression of AURKA and NEK2; Table S1: Dysregulated phosphosites (1.5-fold) across the six cancer types; Table S2: Signature phosphosites (1.5-fold) across the five cancer types; Table S3: Proteins enriched in the cell cycle pathway; Table S4: Kinase-substrate links from KSEA analysis; Table S5: Kinase enrichment score from KSEA analysis.
