# Supplementary material for: Bioinformatics Analysis of Global Proteomic and Phosphoproteomic Data Sets Revealed Activation of NEK2 and AURKA in Cancers
Source: Biomolecules. 2020 Feb 4;10(2):237. doi: 10.3390/biom10020237 (PMC7072708; doi:10.3390/biom10020237)
Supplement: Supplementary file 1 [file biomolecules-10-00237-s001.pdf]

# Bioinformatics Analysis of Global Proteomic and Phosphoproteomic Data Sets Revealed Activation of NEK2 and AURKA in Cancers

**Barnali Deb [1,2,†], Pratyay Sengupta [3,†], Janani Sambath [1] and Prashant Kumar [1,2,*]**

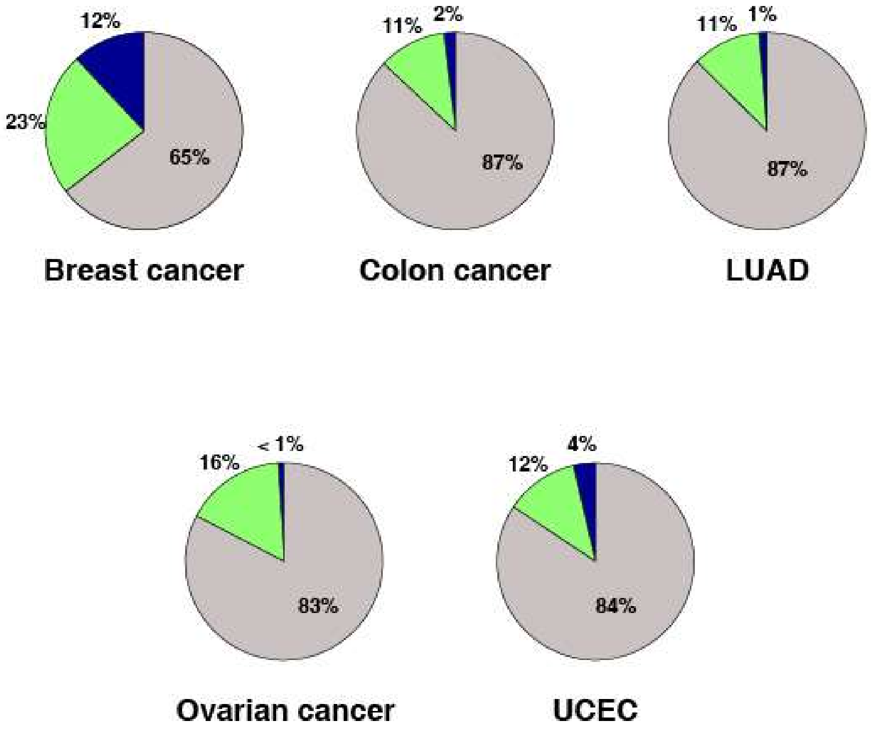


**Figure S1:** The percentage of phosphosites identified across 5 cancer types (breast cancer, colon cancer, LUAD, ovarian cancer and UCEC). Grey, green and blue represent the percentage of serine, threonine and tyrosine sites respectively.

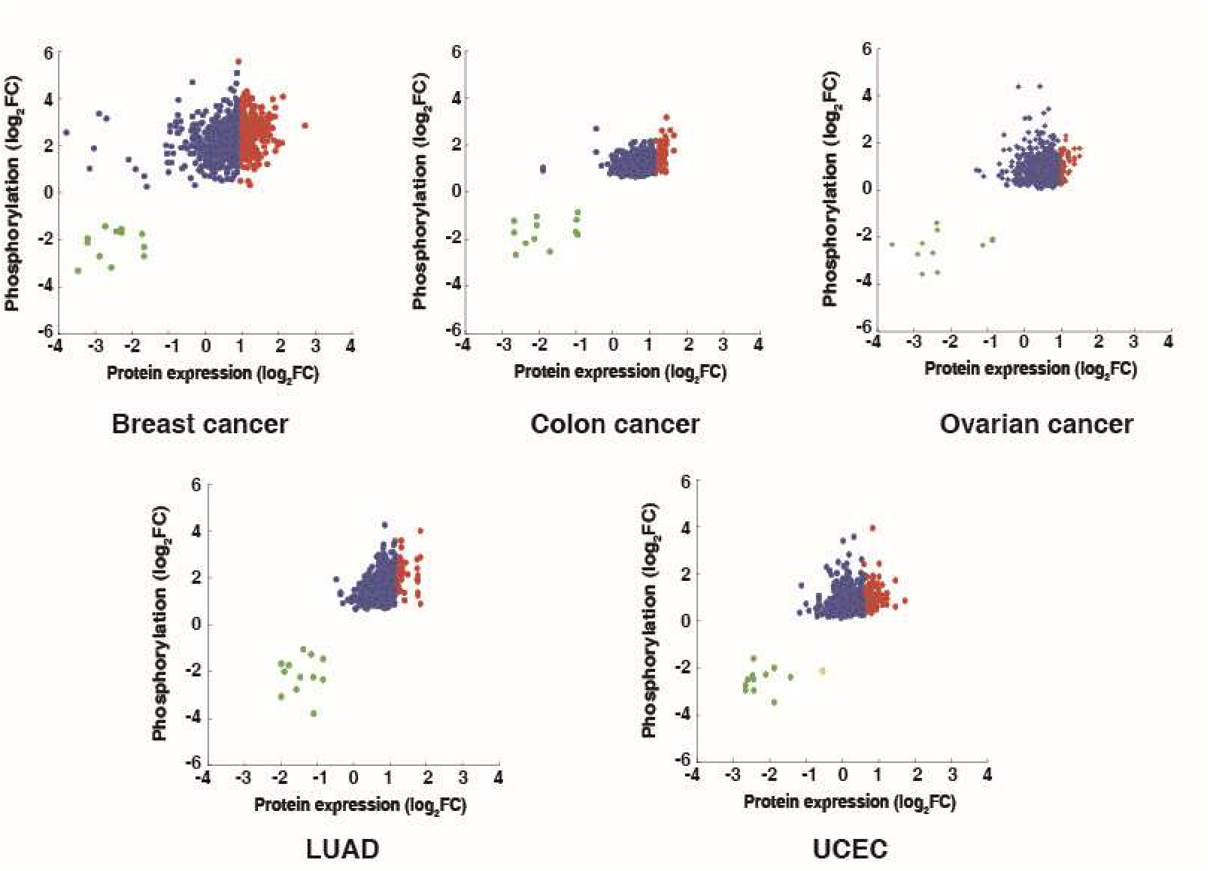


**Figure S2:** Quadrant plot representing the dysregulated phosphopeptides across 5 cancer types and their corresponding protein expressions.

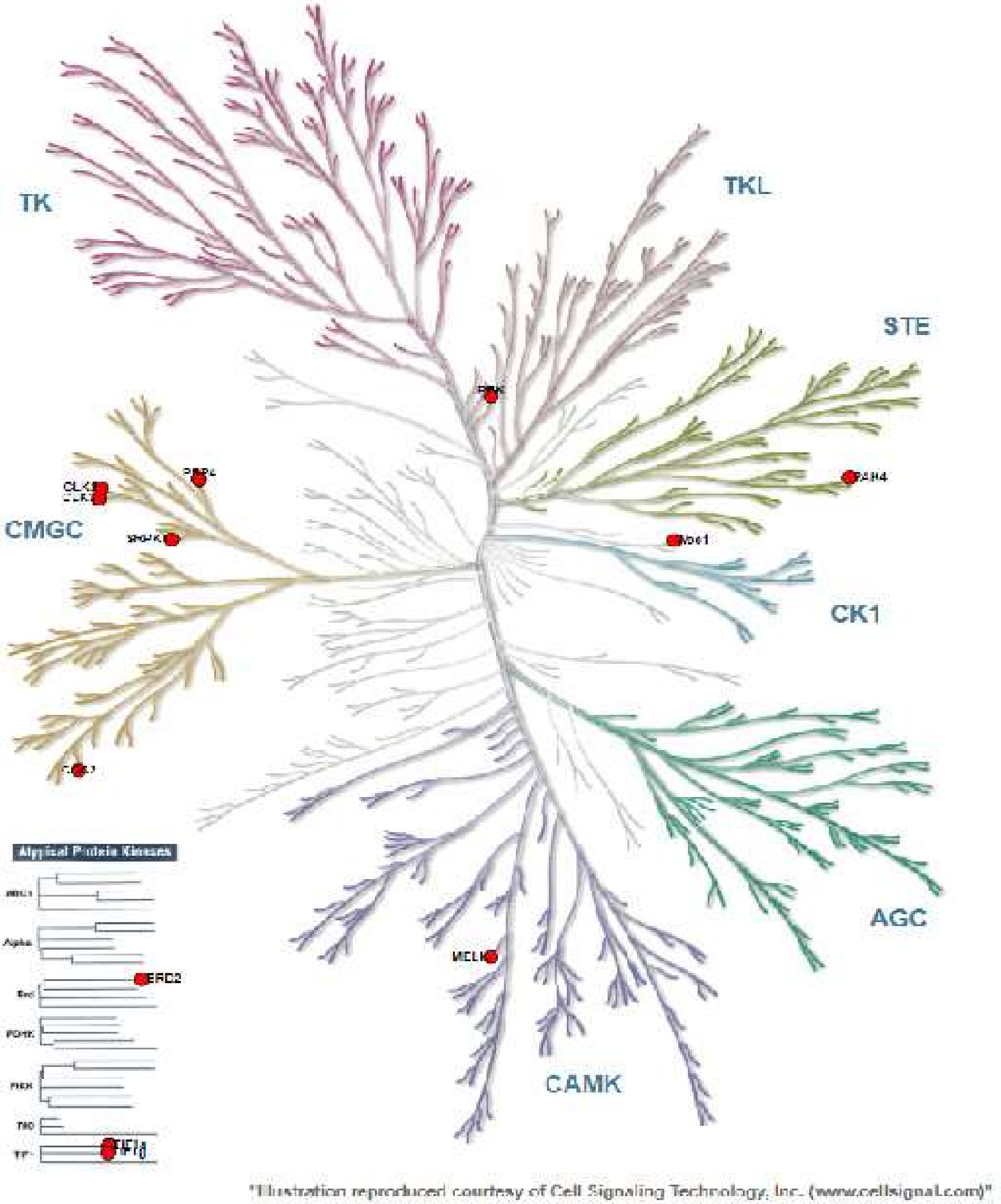


**Figure S3:** Kinome map depicting the identified kinases in the dataset. Kinases are highlighted as in the inset legend. The map was built using the KinMap tool. Illustration reproduced courtesy of Cell Signaling Technology, Inc. (www.cellsignal.com).

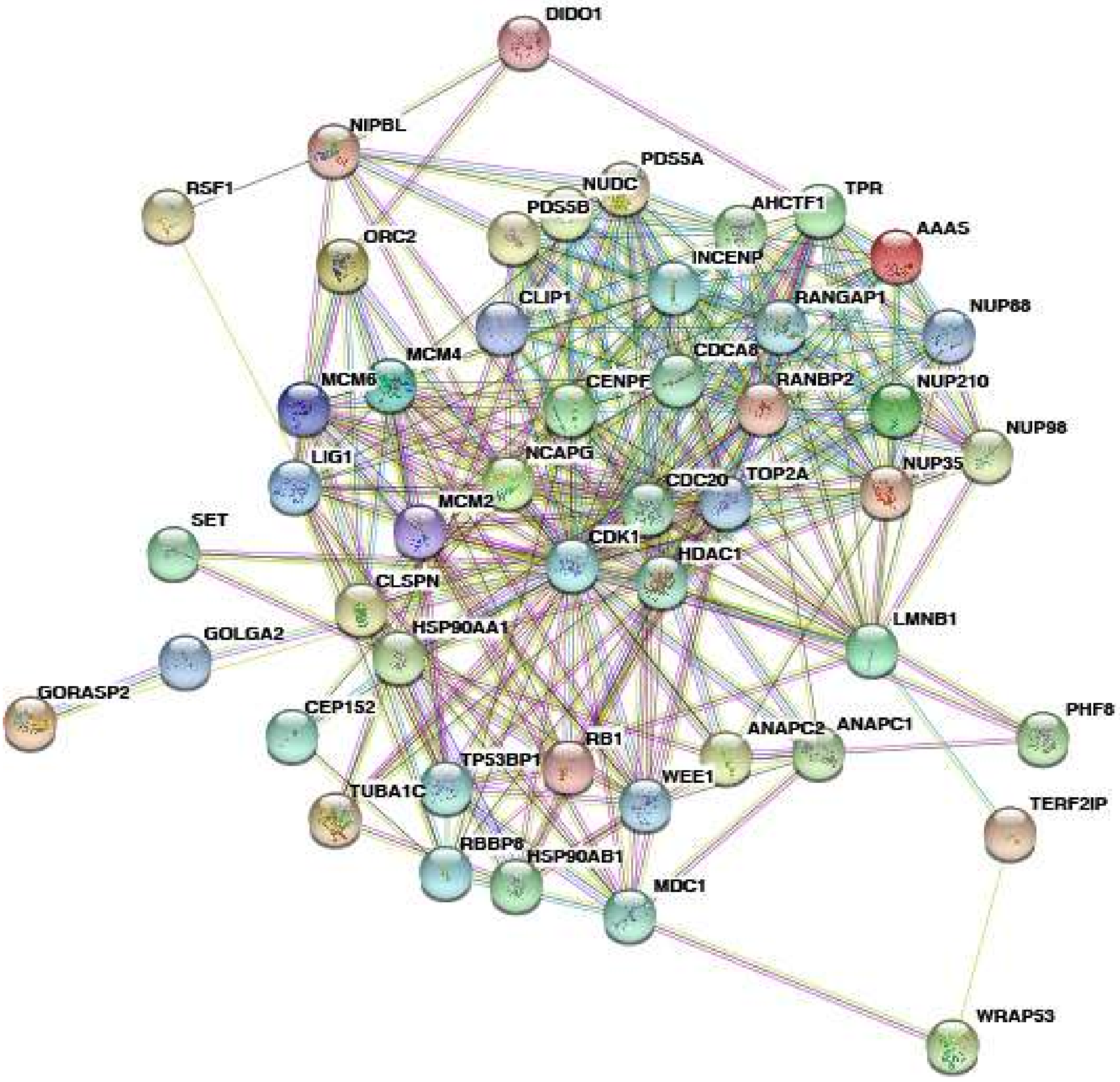


**Figure S4:** Protein-protein interaction network showing the interaction among 48 proteins involved in cell cycle pathway with highest confidence (0.90) acquired using STRING functional protein association network tool.

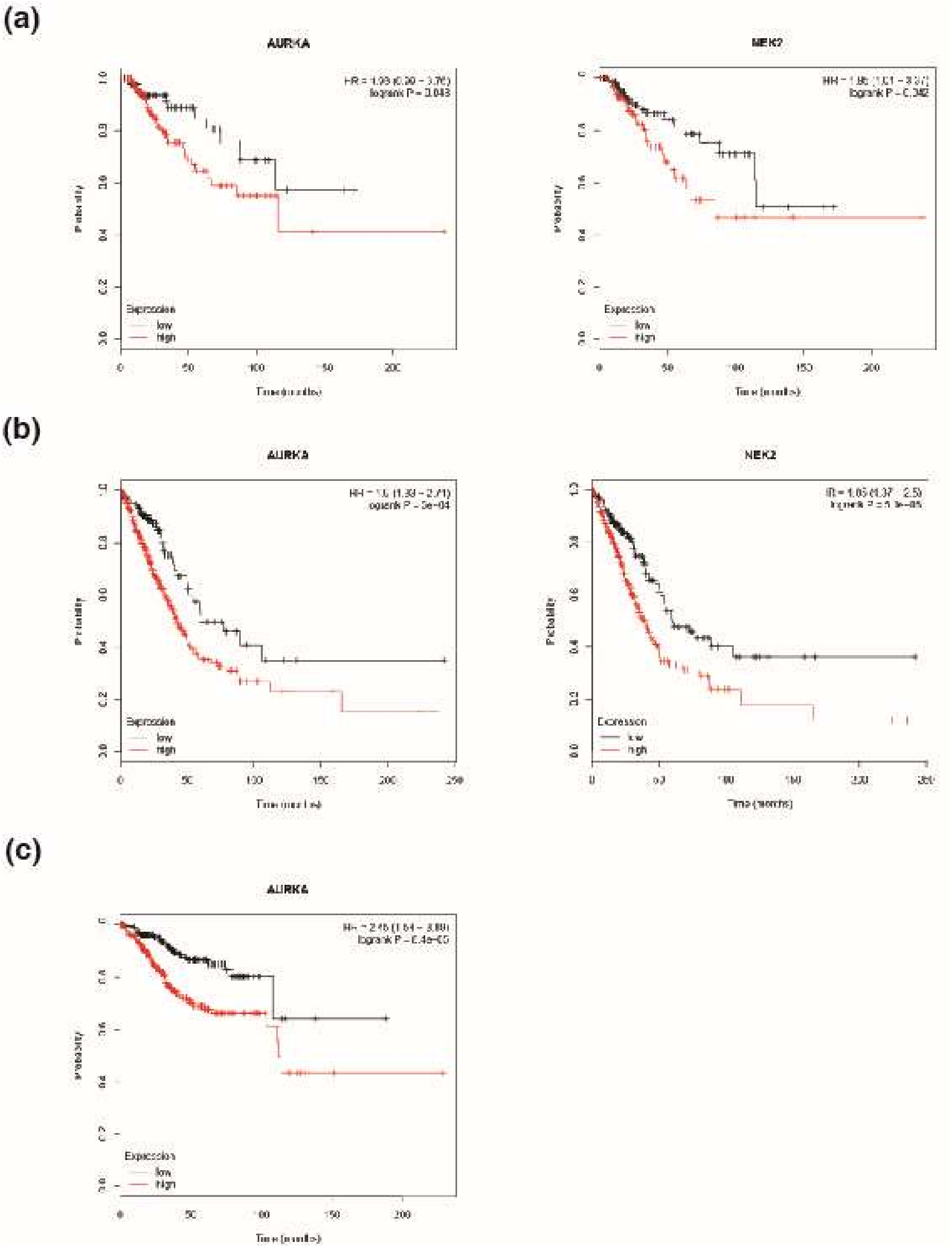


**Figure S5:** Kaplan-Meier plot for (a) breast cancer, (b) LUAD and (c) UCEC patients stratified according to high and low expression of AURKA and NEK2.

**Deb et al., 2019. Integrative global proteomic and phosphoproteomic datasets revealed activation of NEK2 and AURKA in cancer**

## Supplementary tables

Table S1: Dysregulated phosphosites (1.5 fold) across the 6 cancer types

| Protein ID | Site | Breast Cancer (FC) | Colon Cancer (FC) | Lung Adenocarcinoma (FC) | Ovarian Cancer (FC) | Clear Cell Renal Cell Carcinoma (FC) | Uterine Corpus Endometrial Carcinoma (FC) |
|---|---|---|---|---|---|---|---|
| ATRX | S1352 | 6.01 | 1.88 | 2.32 | 2.49 | 0.58 | 4.08 |
| HBA1 | S132 | 0.15 | 0.45 | 0.34 | 0.50 | 2.10 | 0.36 |
| POLR2A | S1917 | 11.35 | 1.92 | 2.67 | 1.52 | 0.61 | 1.88 |
| POLR2A | S1931 | 11.35 | 1.92 | 2.67 | 1.52 | 0.61 | 1.88 |
| CHD3 | T1611 | 9.02 | 1.81 | 3.06 | 3.81 | 0.42 | 3.52 |
| RHBDF2 | S148 | 5.25 | 1.86 | 1.62 | 2.78 | 2.17 | 2.33 |
| TMCC1 | S382 | 4.79 | 1.74 | 2.66 | 1.96 | 2.07 | 3.68 |
| CANX | S554 | 11.16 | 1.91 | 1.98 | 3.00 | 3.28 | 0.51 |
| CANX | S564 | 12.00 | 1.67 | 2.37 | 3.76 | 2.70 | 0.51 |
| CANX | T562 | 11.22 | 1.79 | 1.83 | 3.62 | 2.70 | 0.51 |
| ARFIP1 | S39 | 14.80 | 1.70 | 3.60 | 3.03 | 0.55 | 2.41 |
| FOXK1 | S213 | 5.45 | 3.78 | 3.08 | 4.42 | 2.27 | 2.60 |
| FOXK1 | S223 | 9.41 | 3.78 | 3.70 | 4.65 | 2.27 | 2.09 |
| SRSF5 | S120 | 4.29 | 2.66 | 2.57 | 2.38 | 0.55 | 2.53 |
| SPP1 | S219 | 8.66 | 1.76 | 3.49 | 1.95 | 0.35 | 3.08 |
| SPP1 | S234 | 14.32 | 4.14 | 4.59 | 2.50 | 0.21 | 2.45 |
| SPP1 | S254 | 4.89 | 2.09 | 10.09 | 5.67 | 0.20 | 2.62 |
| SPP1 | S258 | 6.16 | 2.52 | 5.67 | 4.12 | 0.17 | 4.15 |
| SPP1 | S263 | 6.86 | 2.14 | 8.40 | 5.67 | 0.17 | 2.58 |
| SPP1 | S267 | 5.96 | 2.83 | 5.79 | 40.88 | 0.17 | 2.75 |
| ACBD5 | S156 | 12.42 | 1.97 | 9.03 | 1.72 | 0.45 | 1.93 |
| CARHSP1 | S30 | 3.73 | 1.77 | 2.24 | 2.89 | 2.23 | 1.81 |
| CARHSP1 | S32 | 2.70 | 1.77 | 2.19 | 2.99 | 2.23 | 1.95 |
| RERE | S679 | 4.78 | 1.78 | 2.21 | 2.98 | 0.44 | 3.10 |
| MYH14 | S1997 | 9.51 | 1.88 | 2.48 | 1317.20 | 0.45 | 2.62 |
| TLE3 | S205 | 6.33 | 2.09 | 2.79 | 2.13 | 2.16 | 1.90 |
| DDX54 | S782 | 9.15 | 2.75 | 2.73 | 2.01 | 1.77 | 2.34 |
| NDRG1 | S332 | 2.91 | 2.25 | 2.27 | 2.37 | 2.32 | 2.25 |
| TCOF1 | S1339 | 8.82 | 2.01 | 3.58 | 0.64 | 1.72 | 2.07 |
| TCOF1 | S156 | 15.21 | 4.12 | 5.63 | 4.01 | 2.31 | 2.54 |
| RCOR3 | S433 | 5.62 | 1.87 | 1.58 | 2.34 | 0.60 | 0.58 |
| RCOR3 | T434 | 5.62 | 1.89 | 1.75 | 2.34 | 0.60 | 0.53 |

| | | | | | | | |
|---|---|---|---|---|---|---|---|
| TP53BP1 | S1072 | 13.12 | 2.23 | 3.26 | 2.59 | 1.74 | 6.08 |
| TP53BP1 | S1683 | 20.77 | 2.56 | 5.34 | 5.58 | 1.73 | 3.59 |
| PPP1R13L | S134 | 6.72 | 1.88 | 2.06 | 2.27 | 1.77 | 2.01 |
| ZNF652 | S100 | 3.62 | 2.77 | 2.66 | 14.99 | 0.41 | 2.24 |
| KDM3A | S463 | 12.21 | 2.19 | 6.92 | 1.82 | 3.05 | 2.60 |
| SLC25A5 | S42 | 6.51 | 1.89 | 2.97 | 2.53 | 0.27 | 1.73 |
| HTATSF1 | S616 | 13.35 | 2.19 | 2.75 | 2.75 | 0.44 | 2.81 |
| VDAC2 | S130 | 7.37 | 2.32 | 2.64 | 2.49 | 1.77 | 2.17 |
| MATR3 | S604 | 10.28 | 2.20 | 3.04 | 2.49 | 0.60 | 2.02 |
| SRSF2 | S189 | 5.45 | 2.17 | 2.75 | 3.83 | 0.31 | 2.01 |
| PPP1R12B | S17 | 1.71 | 0.52 | 1.52 | 2.28 | 0.59 | 0.11 |
| DPYSL3 | T623 | 4.15 | 0.51 | 2.29 | 2.57 | 1.99 | 0.42 |
| KCNAB2 | S9 | 5.10 | 1.84 | 2.03 | 1.86 | 1.82 | 1.67 |
| RBM15 | S674 | 4.15 | 2.20 | 2.25 | 1.83 | 0.48 | 1.68 |
| SLC20A2 | S256 | 7.96 | 2.11 | 3.04 | 1.68 | 0.42 | 3.10 |
| CDC20 | S41 | 7.33 | 2.04 | 5.46 | 2.97 | 2.23 | 2.51 |
| TJP3 | S112 | 21.02 | 2.85 | 2.18 | 2.45 | 0.15 | 2.43 |
| TJP3 | S319 | 11.21 | 1.85 | 2.27 | 3.26 | 0.23 | 4.58 |
| TJP3 | S327 | 8.53 | 2.06 | 1.95 | 3.26 | 0.54 | 6.50 |
| DOCK7 | S900 | 4.13 | 1.65 | 3.60 | 3.48 | 0.56 | 2.42 |
| FARP1 | S920 | 5.65 | 1.72 | 2.06 | 1.82 | 0.55 | 3.75 |
| TEX2 | S790 | 6.54 | 1.77 | 1.59 | 2.29 | 1.62 | 2.16 |
| HSH2D | S137 | 8.03 | 2.07 | 3.08 | 2.18 | 2.29 | 3.12 |
| BAZ2A | S1768 | 10.93 | 2.38 | 2.33 | 4.41 | 1.66 | 3.80 |
| BCLAF1 | S288 | 6.18 | 2.00 | 2.23 | 2.98 | 0.58 | 2.24 |
| BCLAF1 | S387 | 11.08 | 1.92 | 2.31 | 2.93 | 0.59 | 3.13 |
| SRRM1 | S768 | 8.43 | 2.02 | 3.28 | 2.23 | 0.53 | 2.38 |
| CAD | S1796 | 4.33 | 2.44 | 3.79 | 2.17 | 1.99 | 1.91 |
| HMGA1 | S36 | 6.23 | 2.44 | 2.23 | 3.70 | 0.48 | 2.34 |
| SNX27 | S51 | 3.82 | 2.43 | 2.57 | 1.87 | 0.49 | 1.86 |
| PPP1R35 | S52 | 4.29 | 1.72 | 1.89 | 3.87 | 1.78 | 1.86 |
| CRYBG1 | S22 | 4.46 | 3.19 | 2.35 | 0.46 | 0.43 | 2.09 |
| ARVCF | S343 | 3.43 | 2.61 | 2.24 | 2.21 | 0.42 | 2.42 |

| | | | | | | | |
|---|---|---|---|---|---|---|---|
| ATP2B1 | S1182 | 7.29 | 1.81 | 2.00 | 9.28 | 0.51 | 2.13 |
| CAV1 | S37 | 0.46 | 0.39 | 0.15 | 0.09 | 3.43 | 0.21 |
| DES | S28 | 0.39 | 0.48 | 0.25 | 0.26 | 0.38 | 0.50 |
| DES | S32 | 0.34 | 0.34 | 0.00 | 0.26 | 0.45 | 0.28 |
| DSG2 | S701 | 14.71 | 2.33 | 7.35 | 4.50 | 1.94 | 3.87 |
| ITPR3 | S2670 | 3.05 | 1.94 | 1.66 | 2.38 | 1.82 | 1.87 |
| KRT19 | S14 | 4.65 | 1.57 | 2.55 | 2.73 | 0.36 | 1.96 |
| LSP1 | S177 | 7.28 | 1.68 | 1.97 | 2.53 | 1.85 | 2.13 |
| LSP1 | S188 | 8.93 | 1.75 | 2.00 | 2.53 | 1.87 | 2.04 |
| LSP1 | S189 | 9.40 | 1.82 | 2.59 | 2.12 | 1.86 | 1.84 |
| LSP1 | T175 | 5.30 | 1.83 | 1.76 | 253.06 | 1.87 | 1.94 |
| MARCKS | S145 | 8.47 | 2.08 | 2.63 | 2.50 | 0.44 | 2.30 |
| MARCKS | S147 | 7.29 | 0.63 | 2.62 | 2.50 | 0.44 | 2.80 |
| MARCKS | T150 | 8.02 | 2.08 | 2.08 | 2.11 | 0.58 | 1.85 |
| MKI67 | S357 | 33.29 | 3.02 | 6.39 | 3.24 | 3.09 | 2.79 |
| MKI67 | S374 | 12.51 | 1.82 | 4.84 | 5.52 | 2.69 | 3.69 |
| PRPF4B | S144 | 4.73 | 1.95 | 2.18 | 3.00 | 0.60 | 2.50 |
| DSP | S2825 | 6.78 | 1.81 | 2.29 | 2.36 | 0.56 | 2.91 |
| MCM2 | S139 | 6.95 | 2.69 | 5.32 | 2.64 | 1.94 | 2.06 |
| TRA2B | S264 | 3.27 | 3.47 | 2.71 | 3.56 | 0.50 | 1.95 |
| DLG5 | S1000 | 8.83 | 1.72 | 2.80 | 2.26 | 2.52 | 1.83 |
| PPIG | S745 | 8.50 | 1.96 | 2.48 | 2.24 | 0.45 | 2.48 |
| ABCC1 | S930 | 9.14 | 2.87 | 3.49 | 2.48 | 2.14 | 1.83 |
| AKAP12 | S286 | 0.23 | 0.29 | 0.40 | 0.28 | 0.28 | 0.15 |
| NCL | S34 | 4.75 | 2.59 | 2.80 | 4.75 | 0.33 | 2.35 |
| NCL | S41 | 6.47 | 2.48 | 2.43 | 4.92 | 0.33 | 2.30 |
| NCL | T76 | 6.98 | 2.49 | 4.81 | 4.08 | 2.27 | 2.83 |
| ZRANB2 | S307 | 9.22 | 2.24 | 2.00 | 1.87 | 0.65 | 2.47 |
| LAD1 | S177 | 4.19 | 2.33 | 6.06 | 3.72 | 0.15 | 4.14 |
| LAD1 | S64 | 4.04 | 1.84 | 3.49 | 2.22 | 0.24 | 2.04 |
| STUB1 | S19 | 4.70 | 2.24 | 3.84 | 3.35 | 2.03 | 1.78 |
| STUB1 | S23 | 5.09 | 2.65 | 2.94 | 3.35 | 2.03 | 1.78 |
| MCM6 | S762 | 7.58 | 2.55 | 4.83 | 1.83 | 2.50 | 2.00 |
| RANBP2 | T1396 | 7.28 | 1.90 | 3.39 | 2.92 | 1.82 | 3.03 |
| NCOR2 | S554 | 16.93 | 2.28 | 2.54 | 4.25 | 1.92 | 0.38 |
| SLC12A7 | T973 | 4.38 | 1.52 | 1.99 | 1.93 | 1.79 | 1.95 |

| | | | | | | | |
|---|---|---|---|---|---|---|---|
| NUDC | T145 | 6.17 | 2.91 | 3.18 | 3.89 | 0.23 | 1.92 |
| KIF1C | S676 | 7.75 | 1.68 | 2.20 | 1.85 | 0.53 | 2.34 |
| AKAP13 | S983 | 6.72 | 1.91 | 3.49 | 1.84 | 1.75 | 1.71 |
| AKAP13 | T953 | 4.39 | 1.93 | 2.10 | 2.84 | 1.77 | 1.66 |
| OGFR | S378 | 4.60 | 2.16 | 2.27 | 2.48 | 2.68 | 1.70 |
| RABGAP1 | S56 | 6.11 | 1.90 | 2.36 | 4.79 | 0.52 | 3.15 |
| CAVIN1 | S171 | 0.17 | 0.18 | 0.18 | 0.10 | 1.68 | 0.32 |
| DNTTIP2 | T232 | 3.80 | 2.25 | 2.45 | 2.18 | 0.62 | 2.23 |
| PDAP1 | S63 | 2.60 | 1.75 | 2.70 | 2.71 | 0.34 | 2.06 |
| TBC1D9B | S432 | 3.25 | 2.06 | 1.91 | 5.57 | 1.55 | 2.52 |
| RRP1B | S736 | 2.09 | 2.21 | 1.74 | 2.25 | 2.07 | 2.19 |
| ZC3H4 | S1275 | 7.11 | 1.91 | 2.44 | 2.83 | 1.52 | 1.82 |
| LARP1 | S471 | 4.30 | 2.34 | 2.15 | 2.44 | 2.58 | 1.87 |
| LARP1 | S477 | 5.03 | 1.96 | 1.83 | 2.29 | 2.58 | 0.43 |
| LARP1 | T449 | 3.75 | 1.84 | 2.95 | 2.63 | 2.01 | 1.86 |
| SCRIB | S1306 | 5.01 | 1.74 | 2.20 | 2.67 | 0.60 | 2.09 |
| GPATCH4 | S135 | 12.30 | 3.10 | 5.43 | 3.50 | 1.75 | 3.02 |
| RSL1D1 | S427 | 21.10 | 5.77 | 6.22 | 7.17 | 3.16 | 4.34 |
| EIF5B | S1168 | 4.79 | 1.98 | 3.32 | 2.29 | 1.94 | 2.30 |
| SRRM2 | S1925 | 43.54 | 2.30 | 2.54 | 3.37 | 0.39 | 3.95 |
| SRRM2 | S1948 | 43.54 | 2.30 | 2.54 | 3.37 | 0.39 | 3.95 |
| SRRM2 | S1960 | 43.54 | 2.30 | 2.54 | 3.37 | 0.39 | 3.95 |
| SRRM2 | S783 | 8.76 | 2.05 | 2.24 | 2.30 | 0.55 | 2.28 |
| SRRM2 | S864 | 13.08 | 1.96 | 2.65 | 2.12 | 0.61 | 1.88 |
| SRRM2 | T2034 | 5.69 | 1.98 | 3.33 | 2.90 | 0.46 | 1.72 |
| GOLM1 | S187 | 6.62 | 1.69 | 1.96 | 3.07 | 0.18 | 0.49 |
| GOLM1 | S191 | 6.45 | 2.16 | 1.82 | 0.40 | 0.25 | 0.49 |
| MAPT | T522 | 3.51 | 0.42 | 2.76 | 0.51 | 2.28 | 0.42 |
| IWS1 | S422 | 10.07 | 2.39 | 5.18 | 1.82 | 0.51 | 4.63 |
| IWS1 | S513 | 13.05 | 2.72 | 2.78 | 3.96 | 0.48 | 2.39 |
| ASXL2 | S395 | 8.41 | 2.14 | 2.78 | 2.01 | 0.57 | 1.70 |
| SASH3 | S38 | 4.04 | 2.20 | 2.21 | 2.25 | 1.91 | 2.68 |
| EML4 | S134 | 9.37 | 2.46 | 4.62 | 3.73 | 1.92 | 2.62 |
| EML4 | S146 | 6.70 | 1.76 | 2.84 | 3.46 | 2.13 | 2.48 |
| EML4 | T897 | 5.87 | 1.57 | 1.78 | 4.18 | 1.78 | 1.95 |
| EML4 | T899 | 5.47 | 1.62 | 2.03 | 4.18 | 1.78 | 1.95 |

| | | | | | | | |
|---|---|---|---|---|---|---|---|
| KIAA1217 | S361 | 2.16 | 1.73 | 1.74 | 3.60 | 1.54 | 1.94 |
| ZC3HAV1 | S346 | 6.57 | 2.12 | 3.00 | 7.41 | 1.89 | 1.62 |
| CBX8 | S256 | 10.08 | 1.68 | 3.00 | 8.45 | 2.14 | 2.28 |
| CAMSAP3 | S814 | 13.41 | 2.46 | 3.30 | 3.26 | 0.46 | 2.63 |
| NDUFV3 | S158 | 5.22 | 1.88 | 2.29 | 1.76 | 0.58 | 1.87 |
| PGM5 | S122 | 0.47 | 0.23 | 0.52 | 0.18 | 0.47 | 0.32 |
| SUDS3 | S53 | 8.14 | 2.09 | 2.91 | 2.82 | 0.44 | 2.23 |
| SUDS3 | T49 | 6.97 | 2.31 | 2.29 | 2.38 | 0.50 | 2.28 |
| CCDC86 | S110 | 3.91 | 2.62 | 2.63 | 3.30 | 2.28 | 8.33 |
| IRF2BPL | T636 | 6.69 | 1.84 | 2.36 | 3.47 | 0.56 | 0.56 |
| RNF219 | S210 | 22.08 | 4.84 | 6.29 | 6.00 | 2.24 | 3.15 |
| TANC2 | S400 | 11.51 | 1.97 | 2.91 | 2.92 | 1.73 | 1.76 |
| NSRP1 | S254 | 8.03 | 2.13 | 3.40 | 6.88 | 0.47 | 3.05 |
| CALD1 | S759 | 3.41 | 0.46 | 2.13 | 0.11 | 0.50 | 0.54 |
| CALD1 | S498 | 3.41 | 0.46 | 2.13 | 0.11 | 0.50 | 0.54 |
| PML | S403 | 3.34 | 2.69 | 2.55 | 3.34 | 1.89 | 1.72 |
| PML | T409 | 7.73 | 2.66 | 2.55 | 4.59 | 1.89 | 1.72 |
| AHNAK2 | S851 | 2.95 | 0.38 | 2.98 | 0.25 | 3.73 | 0.44 |
| GLCCI1 | S148 | 3.06 | 1.58 | 2.67 | 2.62 | 1.66 | 2.13 |
| LMNA | S390 | 1.86 | 1.63 | 1.90 | 2.78 | 1.86 | 0.44 |
| LMNA | T409 | 7.87 | 1.93 | 2.53 | 2.76 | 1.55 | 0.29 |
| DIP2B | S146 | 4.62 | 1.74 | 2.52 | 2.07 | 0.63 | 2.17 |
| STT3B | S498 | 7.91 | 2.31 | 2.32 | 3.68 | 0.56 | 2.17 |
| STT3B | S499 | 4.59 | 2.17 | 2.14 | 3.11 | 0.56 | 1.96 |

Table S2: Signature phosphosites (1.5 fold) across the 5 cancer types

| **Protein ID** | **Site** | **Regulation Pattern** | **Breast Cancer** | **Colon Cancer** | **Lung Adenocarcinoma** | **Ovarian Cancer** | **Uterine Corpus Endometrial Carcinoma** |
|---|---|---|---|---|---|---|---|
| MSH6 | S830 | Hyper | 6.44 | 2.12 | 4.58 | 2.96 | 2.13 |
| KRT18 | S30 | Hyper | 8.60 | 1.67 | 5.10 | 3.45 | 3.28 |
| LIG1 | S141 | Hyper | 7.31 | 2.07 | 2.60 | 2.00 | 1.92 |
| LIG1 | S51 | Hyper | 23.87 | 2.34 | 9.96 | 2.89 | 4.68 |

| | | | | | | | |
|---|---|---|---|---|---|---|---|
| LIG1 | T182 | Hyper | 12.82 | 2.03 | 5.48 | 2.06 | 2.46 |
| LIG1 | T183 | Hyper | 9.27 | 2.61 | 6.02 | 4.21 | 3.32 |
| PGK1 | S203 | Hyper | 4.31 | 2.34 | 4.04 | 3.19 | 2.71 |
| RB1 | S249 | Hyper | 10.51 | 2.80 | 2.51 | 2.28 | 2.34 |
| RB1 | S807 | Hyper | 17.25 | 3.05 | 2.32 | 3.22 | 2.14 |
| RB1 | T373 | Hyper | 3.69 | 2.05 | 2.85 | 1.86 | 2.78 |
| SMN1 | S31 | Hyper | 5.73 | 2.14 | 2.52 | 2.10 | 2.30 |
| KRT17 | S32 | Hyper | 2.27 | 2.22 | 2.35 | 2.76 | 2.09 |
| ATRX | S1352 | Hyper | 6.01 | 1.88 | 2.32 | 2.49 | 4.08 |
| ATRX | S1996 | Hyper | 6.42 | 1.87 | 2.41 | 2.82 | 2.12 |
| HBA1 | S132 | Hypo | 0.15 | 0.45 | 0.34 | 0.50 | 0.36 |
| DCK | T72 | Hyper | 8.12 | 1.73 | 2.15 | 1.81 | 1.83 |
| POLR2A | S1850 | Hyper | 5.23 | 2.28 | 2.69 | 1.86 | 1.65 |
| POLR2A | S1917 | Hyper | 11.35 | 1.92 | 2.67 | 1.52 | 1.88 |
| POLR2A | S1931 | Hyper | 11.35 | 1.92 | 2.67 | 1.52 | 1.88 |
| RPL12 | S38 | Hyper | 8.00 | 3.38 | 5.91 | 3.95 | 3.71 |
| RPL18A | S123 | Hyper | 4.65 | 1.80 | 2.85 | 2.19 | 3.33 |
| AFTPH | S382 | Hyper | 8.12 | 2.44 | 2.06 | 1.91 | 2.42 |
| AFTPH | S411 | Hyper | 14.56 | 1.65 | 3.14 | 5.57 | 2.03 |
| RAB11FIP1 | S389 | Hyper | 17.00 | 2.45 | 2.75 | 2.15 | 2.62 |
| GPATCH8 | S740 | Hyper | 9.66 | 2.35 | 2.61 | 3.63 | 2.03 |
| GPATCH8 | S914 | Hyper | 12.04 | 2.43 | 2.61 | 2.83 | 3.01 |
| GPATCH8 | S981 | Hyper | 3.92 | 1.99 | 2.15 | 2.38 | 1.86 |
| PKP2 | S155 | Hyper | 8.67 | 2.04 | 1.83 | 3.53 | 2.22 |
| CHD3 | T1611 | Hyper | 9.02 | 1.81 | 3.06 | 3.81 | 3.52 |
| RHBDF2 | S148 | Hyper | 5.25 | 1.86 | 1.62 | 2.78 | 2.33 |
| WASHC2A | S158 | Hyper | 4.86 | 1.73 | 1.64 | 3.96 | 1.73 |
| ARHGAP17 | S575 | Hyper | 4.96 | 2.16 | 1.97 | 2.00 | 1.79 |
| ATN1 | S101 | Hyper | 7.85 | 1.87 | 2.11 | 3.60 | 2.16 |
| USP20 | S413 | Hyper | 11.93 | 1.69 | 3.20 | 2.01 | 1.82 |
| ABI1 | S183 | Hyper | 4.53 | 1.61 | 1.80 | 2.16 | 1.69 |
| PAK4 | S104 | Hyper | 4.60 | 1.81 | 1.92 | 2.45 | 1.70 |
| ZNF638 | S128 | Hyper | 8.02 | 1.85 | 3.47 | 2.43 | 1.74 |
| RTKN | S106 | Hyper | 11.60 | 2.56 | 1.81 | 2.81 | 2.55 |
| RTKN | S543 | Hyper | 7.19 | 2.11 | 2.38 | 3.19 | 2.56 |
| PHF6 | S154 | Hyper | 5.64 | 2.93 | 3.00 | 3.31 | 3.29 |
| TMCC1 | S382 | Hyper | 4.79 | 1.74 | 2.66 | 1.96 | 3.68 |
| HSP90AA1 | S385 | Hyper | 4.68 | 1.65 | 2.16 | 2.58 | 1.74 |
| FNBP1L | S443 | Hyper | 4.07 | 1.87 | 2.63 | 2.96 | 2.23 |
| ABCF1 | S109 | Hyper | 2.12 | 2.46 | 2.79 | 3.00 | 1.86 |
| ARFIP1 | S39 | Hyper | 14.80 | 1.70 | 3.60 | 3.03 | 2.41 |
| NOP2 | S177 | Hyper | 10.95 | 2.34 | 2.82 | 2.96 | 1.66 |
| NOP2 | S67 | Hyper | 26.05 | 5.71 | 4.38 | 3.71 | 1.97 |

| | | | | | | | |
|---|---|---|---|---|---|---|---|
| GTF3C2 | S167 | Hyper | 12.83 | 2.16 | 1.90 | 3.66 | 2.05 |
| FOXK1 | S213 | Hyper | 5.45 | 3.78 | 3.08 | 4.42 | 2.60 |
| FOXK1 | S223 | Hyper | 9.41 | 3.78 | 3.70 | 4.65 | 2.09 |
| FOXK1 | S257 | Hyper | 2.91 | 1.82 | 1.76 | 1.54 | 2.08 |
| FOXK1 | S441 | Hyper | 5.41 | 2.09 | 1.91 | 2.16 | 2.74 |
| FOXK1 | S445 | Hyper | 4.51 | 2.59 | 1.99 | 2.21 | 2.74 |
| FOXK1 | T436 | Hyper | 4.68 | 2.01 | 2.18 | 3.15 | 1.99 |
| FOXK1 | Y219 | Hyper | 9.09 | 4.05 | 4.30 | 3.61 | 2.40 |
| NCKAP5L | S630 | Hyper | 9.19 | 2.01 | 3.13 | 9.63 | 2.19 |
| SRSF5 | S120 | Hyper | 4.29 | 2.66 | 2.57 | 2.38 | 2.53 |
| SRSF5 | S233 | Hyper | 2.55 | 2.63 | 2.94 | 2.60 | 4.06 |
| SPP1 | S219 | Hyper | 8.66 | 1.76 | 3.49 | 1.95 | 3.08 |
| SPP1 | S234 | Hyper | 14.32 | 4.14 | 4.59 | 2.50 | 2.45 |
| SPP1 | S254 | Hyper | 4.89 | 2.09 | 10.09 | 5.67 | 2.62 |
| SPP1 | S258 | Hyper | 6.16 | 2.52 | 5.67 | 4.12 | 4.15 |
| SPP1 | S263 | Hyper | 6.86 | 2.14 | 8.40 | 5.67 | 2.58 |
| SPP1 | S267 | Hyper | 5.96 | 2.83 | 5.79 | 40.88 | 2.75 |
| INCENP | S263 | Hyper | 4.01 | 2.40 | 1.86 | 1.98 | 2.24 |
| ANKZF1 | S56 | Hyper | 14.78 | 3.14 | 3.81 | 4.36 | 3.75 |
| ACBD5 | S156 | Hyper | 12.42 | 1.97 | 9.03 | 1.72 | 1.93 |
| ACBD5 | S374 | Hyper | 20.45 | 2.06 | 6.17 | 1.71 | 2.97 |
| CARHSP1 | S30 | Hyper | 3.73 | 1.77 | 2.24 | 2.89 | 1.81 |
| CARHSP1 | S32 | Hyper | 2.70 | 1.77 | 2.19 | 2.99 | 1.95 |
| CARHSP1 | T45 | Hyper | 2.67 | 1.63 | 2.06 | 9.40 | 2.73 |
| RRBP1 | S185 | Hyper | 8.57 | 2.93 | 5.60 | 2.51 | 2.01 |
| RRBP1 | S526 | Hyper | 3.74 | 1.72 | 2.98 | 1.92 | 1.70 |
| PARP2 | S33 | Hyper | 16.92 | 6.02 | 3.50 | 3.03 | 5.96 |
| PARP2 | S34 | Hyper | 7.59 | 3.06 | 1.91 | 2.44 | 2.26 |
| RERE | S679 | Hyper | 4.78 | 1.78 | 2.21 | 2.98 | 3.10 |
| UHRF1 | S287 | Hyper | 9.37 | 3.24 | 4.15 | 2.54 | 3.58 |
| TOP2A | S1106 | Hyper | 25.82 | 4.31 | 6.40 | 3.81 | 1.89 |
| TOP2A | S1247 | Hyper | 23.54 | 3.85 | 13.14 | 5.44 | 3.49 |
| TOP2A | S1393 | Hyper | 19.41 | 8.32 | 1.81 | 4.07 | 2.31 |
| TOP2B | S1335 | Hyper | 5.40 | 3.15 | 2.86 | 1.72 | 2.59 |
| TOP2B | S1337 | Hyper | 4.16 | 3.15 | 2.45 | 2.04 | 2.28 |
| TOP2B | S1408 | Hyper | 5.64 | 1.92 | 2.19 | 3.69 | 2.37 |
| TOP2B | T1398 | Hyper | 6.58 | 2.02 | 2.16 | 2.63 | 2.37 |
| ACSS2 | S267 | Hyper | 1.68 | 1.96 | 2.07 | 2.11 | 1.94 |
| ZC3H13 | S831 | Hyper | 6.83 | 2.14 | 2.23 | 2.05 | 3.03 |
| ZC3H13 | S833 | Hyper | 6.56 | 2.17 | 2.23 | 1.84 | 3.64 |
| ZC3H13 | S877 | Hyper | 4.16 | 2.24 | 1.72 | 2.19 | 2.23 |
| ZC3H13 | S993 | Hyper | 8.15 | 1.92 | 2.60 | 2.43 | 1.66 |
| MYH14 | S1991 | Hyper | 9.51 | 1.94 | 2.34 | 1317.20 | 2.79 |

| | | | | | | | |
|---|---|---|---|---|---|---|---|
| MYH14 | S1997 | Hyper | 9.51 | 1.88 | 2.48 | 1317.20 | 2.62 |
| SEC31A | S799 | Hyper | 7.72 | 1.84 | 2.97 | 2.69 | 1.69 |
| IRF2BP2 | S344 | Hyper | 5.86 | 2.45 | 2.22 | 2.88 | 1.71 |
| HNRNPC | S220 | Hyper | 7.47 | 1.86 | 2.34 | 4.61 | 1.90 |
| HNRNPC | S225 | Hyper | 12.37 | 1.96 | 2.88 | 2.83 | 1.61 |
| HNRNPC | S226 | Hyper | 12.49 | 2.08 | 2.58 | 3.53 | 2.15 |
| KTN1 | S75 | Hyper | 9.50 | 1.71 | 2.15 | 3.91 | 2.07 |
| KTN1 | T153 | Hyper | 8.15 | 1.57 | 3.12 | 2.08 | 1.88 |
| PAICS | S27 | Hyper | 5.91 | 2.40 | 5.56 | 2.13 | 3.09 |
| HNRNPUL2 | S168 | Hyper | 19.97 | 2.06 | 1.81 | 2.07 | 2.22 |
| DIAPH1 | S22 | Hyper | 4.49 | 2.08 | 2.47 | 3.35 | 2.72 |
| CCDC88C | S227 | Hyper | 13.13 | 2.00 | 2.55 | 2.00 | 2.94 |
| U2SURP | S67 | Hyper | 13.85 | 1.81 | 4.37 | 3.66 | 2.60 |
| MYO1C | S1022 | Hypo | 0.44 | 0.32 | 0.25 | 0.24 | 0.30 |
| THOC2 | S1393 | Hyper | 4.79 | 1.95 | 1.66 | 2.09 | 3.53 |
| TMEM214 | T97 | Hyper | 16.16 | 3.04 | 4.53 | 2.08 | 2.30 |
| ACLY | S481 | Hyper | 6.30 | 1.80 | 1.87 | 2.38 | 1.82 |
| ZBTB21 | S435 | Hyper | 3.65 | 2.37 | 3.57 | 3.02 | 1.60 |
| HDAC7 | T413 | Hyper | 4.94 | 1.83 | 1.58 | 2.22 | 1.71 |
| BAG6 | S958 | Hyper | 5.17 | 2.04 | 2.30 | 3.05 | 1.83 |
| BAG6 | S967 | Hyper | 5.39 | 2.12 | 2.30 | 3.05 | 2.04 |
| AKT1S1 | S203 | Hyper | 6.51 | 2.35 | 2.12 | 1.92 | 2.01 |
| AKT1S1 | S211 | Hyper | 8.79 | 2.31 | 2.20 | 2.07 | 2.22 |
| AKT1S1 | S212 | Hyper | 9.90 | 2.31 | 2.54 | 2.52 | 1.92 |
| PDS5A | S1305 | Hyper | 4.68 | 2.06 | 2.04 | 1.98 | 1.90 |
| NRDC | S96 | Hyper | 8.31 | 1.79 | 2.04 | 2.15 | 2.33 |
| ZFAND5 | S48 | Hyper | 3.88 | 1.76 | 2.53 | 2.72 | 1.98 |
| TLE3 | S205 | Hyper | 6.33 | 2.09 | 2.79 | 2.13 | 1.90 |
| TLE3 | S286 | Hyper | 7.31 | 1.81 | 2.59 | 1.78 | 1.78 |
| MYBBP1A | S1163 | Hyper | 6.15 | 3.45 | 2.76 | 3.79 | 2.77 |
| UBE4B | S124 | Hyper | 2.87 | 1.85 | 2.14 | 2.59 | 1.72 |
| DDX54 | S782 | Hyper | 9.15 | 2.75 | 2.73 | 2.01 | 2.34 |
| BRD2 | S301 | Hyper | 4.34 | 1.56 | 2.34 | 1.64 | 1.67 |
| LSM14A | S183 | Hyper | 3.57 | 1.91 | 1.76 | 2.54 | 1.76 |
| NCBP3 | S415 | Hyper | 10.03 | 2.34 | 3.17 | 1.98 | 1.66 |
| PHKA1 | S758 | Hyper | 3.68 | 3.07 | 5.06 | 2.74 | 3.08 |
| SET | S63 | Hyper | 8.55 | 3.05 | 2.92 | 1.99 | 1.76 |
| SET | S7 | Hyper | 12.31 | 1.89 | 2.58 | 3.26 | 2.35 |
| RACGAP1 | S203 | Hyper | 10.29 | 1.92 | 6.38 | 2.53 | 4.51 |
| RACGAP1 | T342 | Hyper | 8.41 | 2.26 | 4.24 | 2.68 | 2.81 |
| TFRC | S34 | Hyper | 7.18 | 2.82 | 2.70 | 3.09 | 2.84 |
| CLK3 | S224 | Hyper | 4.30 | 2.19 | 2.49 | 1.83 | 1.68 |
| CLK3 | S226 | Hyper | 4.30 | 1.92 | 2.12 | 14.71 | 1.64 |

| | | | | | | | |
|---|---|---|---|---|---|---|---|
| CLDN5 | T292 | Hypo | 0.46 | 0.44 | 0.09 | 0.19 | 0.19 |
| MEF2A | S108 | Hyper | 2.87 | 1.65 | 1.84 | 5.23 | 3.61 |
| ARHGEF18 | S733 | Hyper | 5.01 | 2.15 | 1.84 | 2.78 | 1.87 |
| TBC1D5 | S796 | Hyper | 2.60 | 2.00 | 2.22 | 2.07 | 1.79 |
| NDRG1 | S332 | Hyper | 2.91 | 2.25 | 2.27 | 2.37 | 2.25 |
| TCOF1 | S1193 | Hyper | 3.08 | 2.20 | 2.70 | 2.03 | 1.57 |
| TCOF1 | S156 | Hyper | 15.21 | 4.12 | 5.63 | 4.01 | 2.54 |
| TCOF1 | S381 | Hyper | 9.06 | 3.02 | 3.52 | 3.33 | 2.02 |
| TCOF1 | T1072 | Hyper | 7.05 | 2.18 | 2.12 | 2.59 | 1.84 |
| TCOF1 | T249 | Hyper | 8.07 | 1.96 | 3.12 | 2.02 | 1.96 |
| TCOF1 | T914 | Hyper | 10.15 | 2.47 | 2.71 | 2.11 | 1.71 |
| GRAMD1A | S260 | Hyper | 9.49 | 3.16 | 5.34 | 7.51 | 3.30 |
| TRAF3IP1 | S359 | Hyper | 7.12 | 1.80 | 1.83 | 2.01 | 2.27 |
| TP53BP1 | S1033 | Hyper | 5.85 | 2.05 | 1.85 | 2.55 | 2.22 |
| TP53BP1 | S1072 | Hyper | 13.12 | 2.23 | 3.26 | 2.59 | 6.08 |
| TP53BP1 | S1073 | Hyper | 16.94 | 2.74 | 3.28 | 3.81 | 6.08 |
| TP53BP1 | S1099 | Hyper | 11.98 | 2.68 | 2.70 | 2.52 | 1.82 |
| TP53BP1 | S1106 | Hyper | 11.98 | 3.11 | 2.78 | 2.72 | 1.79 |
| TP53BP1 | S1431 | Hyper | 5.22 | 2.22 | 3.28 | 1.92 | 1.74 |
| TP53BP1 | S1623 | Hyper | 6.94 | 2.15 | 4.12 | 1.68 | 2.41 |
| TP53BP1 | S1683 | Hyper | 20.77 | 2.56 | 5.34 | 5.58 | 3.59 |
| TP53BP1 | S530 | Hyper | 9.01 | 2.10 | 7.03 | 1.99 | 1.71 |
| TP53BP1 | S644 | Hyper | 5.41 | 1.70 | 2.80 | 2.22 | 1.89 |
| TP53BP1 | S645 | Hyper | 4.63 | 1.70 | 2.39 | 1.94 | 1.89 |
| TP53BP1 | S836 | Hyper | 3.90 | 1.83 | 2.36 | 2.29 | 1.98 |
| TP53BP1 | S839 | Hyper | 3.90 | 3.25 | 2.46 | 2.71 | 2.21 |
| PPP1R13L | S134 | Hyper | 6.72 | 1.88 | 2.06 | 2.27 | 2.01 |
| SAR1A | T139 | Hyper | 3.29 | 1.71 | 2.23 | 2.04 | 2.37 |
| ARHGAP32 | S952 | Hyper | 5.42 | 1.89 | 2.06 | 1.89 | 2.32 |
| ARHGAP32 | T1236 | Hyper | 5.31 | 1.84 | 2.27 | 2.07 | 3.61 |
| C17orf49 | S96 | Hyper | 6.82 | 2.96 | 4.94 | 2.41 | 2.95 |
| WRAP53 | S26 | Hyper | 8.46 | 2.14 | 2.93 | 2.73 | 1.95 |
| CTPS2 | S571 | Hyper | 5.08 | 1.68 | 2.37 | 334.30 | 2.46 |
| CTPS2 | S574 | Hyper | 5.11 | 1.81 | 2.28 | 2.86 | 1.70 |
| RRP12 | T77 | Hyper | 5.68 | 2.32 | 2.43 | 1.77 | 2.23 |
| KIAA1671 | S1701 | Hyper | 6.47 | 3.13 | 3.11 | 3.49 | 2.10 |
| ELF1 | S143 | Hyper | 3.01 | 1.98 | 1.78 | 2.14 | 2.10 |
| SIN3A | S860 | Hyper | 8.57 | 2.42 | 2.25 | 3.04 | 1.92 |
| ZNF652 | S100 | Hyper | 3.62 | 2.77 | 2.66 | 14.99 | 2.24 |
| RBM17 | T71 | Hyper | 6.26 | 1.61 | 2.58 | 2.73 | 1.76 |
| KDM3A | S463 | Hyper | 12.21 | 2.19 | 6.92 | 1.82 | 2.60 |
| SLC25A5 | S42 | Hyper | 6.51 | 1.89 | 2.97 | 2.53 | 1.73 |
| RBMXL1 | S208 | Hyper | 5.08 | 2.32 | 3.12 | 2.51 | 1.67 |

| | | | | | | | |
|---|---|---|---|---|---|---|---|
| RBMXL1 | S58 | Hyper | 6.96 | 1.79 | 2.52 | 2.08 | 1.73 |
| HTATSF1 | S529 | Hyper | 11.27 | 2.02 | 4.15 | 3.65 | 2.28 |
| HTATSF1 | S616 | Hyper | 13.35 | 2.19 | 2.75 | 2.75 | 2.81 |
| PTBP3 | S426 | Hyper | 3.37 | 3.21 | 3.00 | 2.61 | 2.51 |
| CHAMP1 | S204 | Hyper | 8.63 | 1.97 | 2.08 | 3.45 | 1.92 |
| CHAMP1 | S214 | Hyper | 9.07 | 2.27 | 2.72 | 2.82 | 1.88 |
| CHAMP1 | S386 | Hyper | 3.67 | 2.34 | 1.90 | 2.26 | 2.01 |
| CHAMP1 | S432 | Hyper | 7.78 | 1.76 | 1.71 | 3.67 | 2.02 |
| FLNB | S1536 | Hyper | 7.55 | 2.97 | 3.44 | 3.09 | 2.31 |
| RAPGEF6 | S1502 | Hyper | 2.77 | 1.65 | 1.85 | 1.95 | 1.96 |
| SKA3 | S155 | Hyper | 4.78 | 2.02 | 2.36 | 2.27 | 1.98 |
| UTP14A | S353 | Hyper | 4.69 | 2.24 | 2.28 | 6.89 | 2.36 |
| CHD8 | S1420 | Hyper | 5.57 | 1.74 | 3.19 | 3.94 | 2.06 |
| CHD8 | S1424 | Hyper | 5.57 | 1.74 | 3.47 | 3.17 | 2.68 |
| LAS1L | S543 | Hyper | 9.13 | 1.76 | 2.60 | 2.18 | 1.88 |
| DCAF1 | S978 | Hyper | 3.86 | 2.43 | 2.34 | 2.23 | 1.90 |
| RAB3GAP1 | T542 | Hyper | 10.27 | 2.09 | 3.05 | 1.83 | 2.32 |
| SHROOM1 | T103 | Hyper | 3.33 | 1.92 | 2.91 | 2.30 | 1.58 |
| AAAS | S462 | Hyper | 5.27 | 2.84 | 2.52 | 3.93 | 2.09 |
| CARMIL1 | S122 | Hyper | 5.49 | 2.30 | 2.09 | 3.77 | 1.62 |
| CARMIL1 | S1282 | Hyper | 4.16 | 1.76 | 1.91 | 2.60 | 1.92 |
| BRD7 | S279 | Hyper | 8.98 | 1.93 | 2.21 | 2.87 | 1.72 |
| SMG7 | S739 | Hyper | 5.15 | 1.98 | 3.35 | 1.89 | 2.29 |
| SRPRA | S270 | Hyper | 10.57 | 2.75 | 2.25 | 6.30 | 1.84 |
| SF1 | S205 | Hyper | 7.46 | 1.82 | 2.40 | 1.88 | 2.45 |
| VDAC2 | S130 | Hyper | 7.37 | 2.32 | 2.64 | 2.49 | 2.17 |
| PHF8 | S804 | Hyper | 9.45 | 2.14 | 2.11 | 2.06 | 4.37 |
| DGCR8 | S95 | Hyper | 4.13 | 1.66 | 2.13 | 2.14 | 1.86 |
| CLSPN | S83 | Hyper | 33.47 | 4.85 | 10.01 | 4.39 | 3.21 |
| DIDO1 | S152 | Hyper | 4.96 | 2.43 | 2.39 | 2.25 | 2.39 |
| DIDO1 | S805 | Hyper | 4.54 | 1.90 | 1.83 | 2.42 | 2.59 |
| DIDO1 | S809 | Hyper | 5.61 | 1.81 | 1.92 | 1.74 | 1.94 |
| MATR3 | S598 | Hyper | 7.40 | 2.07 | 2.40 | 2.09 | 2.04 |
| MATR3 | S604 | Hyper | 10.28 | 2.20 | 3.04 | 2.49 | 2.02 |
| MATR3 | S766 | Hyper | 10.33 | 1.55 | 2.58 | 3.98 | 1.86 |
| CEP152 | S1461 | Hyper | 5.50 | 1.97 | 2.87 | 2.51 | 2.19 |
| DENND1B | S596 | Hyper | 4.51 | 1.69 | 2.78 | 2.17 | 1.97 |
| SRSF2 | S189 | Hyper | 5.45 | 2.17 | 2.75 | 3.83 | 2.01 |
| SRSF2 | S191 | Hyper | 5.45 | 2.64 | 2.75 | 3.48 | 2.38 |
| CLINT1 | S227 | Hyper | 8.32 | 1.76 | 2.54 | 4.03 | 1.93 |
| CLINT1 | S299 | Hyper | 7.40 | 1.67 | 2.61 | 2.84 | 3.12 |
| CLINT1 | T294 | Hyper | 8.30 | 1.71 | 3.04 | 2.23 | 2.37 |
| KMT2A | S351 | Hyper | 5.62 | 2.67 | 2.58 | 2.91 | 1.66 |

| | | | | | | | |
|---|---|---|---|---|---|---|---|
| EIF4G3 | S231 | Hyper | 5.89 | 1.62 | 2.35 | 1.69 | 1.92 |
| LRBA | S2485 | Hyper | 3.45 | 1.72 | 2.94 | 2.94 | 2.00 |
| KCNAB2 | S9 | Hyper | 5.10 | 1.84 | 2.03 | 1.86 | 1.67 |
| PGM3 | S92 | Hyper | 4.23 | 1.82 | 2.53 | 2.55 | 2.12 |
| SAFB | S383 | Hyper | 6.03 | 2.60 | 2.54 | 2.84 | 2.01 |
| SAFB | S761 | Hyper | 9.80 | 1.74 | 3.19 | 2.65 | 1.80 |
| CORO7 | S21 | Hyper | 4.89 | 1.56 | 2.27 | 2.53 | 1.72 |
| RBM15 | S656 | Hyper | 11.07 | 2.19 | 2.29 | 3.41 | 1.75 |
| RBM15 | S670 | Hyper | 6.68 | 2.66 | 2.68 | 2.84 | 2.11 |
| RBM15 | S674 | Hyper | 4.15 | 2.20 | 2.25 | 1.83 | 1.68 |
| RBM15 | S700 | Hyper | 6.05 | 2.15 | 2.89 | 3.03 | 1.68 |
| RBM15 | S741 | Hyper | 11.85 | 2.30 | 2.69 | 3.62 | 1.92 |
| EZH2 | T487 | Hyper | 11.82 | 2.08 | 3.33 | 1.67 | 2.40 |
| RBM10 | S801 | Hyper | 8.27 | 1.97 | 2.12 | 1.66 | 1.75 |
| RBM10 | S803 | Hyper | 8.27 | 2.80 | 2.16 | 1.75 | 1.93 |
| RBM10 | S862 | Hyper | 4.17 | 1.56 | 2.09 | 2.58 | 1.76 |
| RBM10 | T156 | Hyper | 4.47 | 1.68 | 2.20 | 1.96 | 2.16 |
| ASPM | S425 | Hyper | 5.25 | 1.73 | 2.89 | 3.14 | 1.83 |
| AKAP1 | S592 | Hyper | 14.55 | 2.41 | 5.09 | 2.46 | 1.70 |
| CLIP1 | S204 | Hyper | 4.27 | 1.86 | 2.17 | 2.02 | 1.93 |
| KRT8 | S302 | Hyper | 10.25 | 2.29 | 5.48 | 3.29 | 2.70 |
| KRT8 | S52 | Hyper | 9.30 | 1.87 | 2.05 | 2.83 | 3.16 |
| E2F8 | S417 | Hyper | 5.02 | 2.43 | 4.82 | 2.38 | 2.26 |
| MELK | S457 | Hyper | 10.50 | 2.44 | 2.34 | 2.35 | 2.32 |
| CDCA8 | S219 | Hyper | 6.43 | 2.72 | 4.46 | 3.46 | 2.59 |
| SLC20A2 | S256 | Hyper | 7.96 | 2.11 | 3.04 | 1.68 | 3.10 |
| HNRNPH1 | S63 | Hyper | 4.63 | 2.38 | 2.55 | 2.41 | 2.05 |
| CDC20 | S41 | Hyper | 7.33 | 2.04 | 5.46 | 2.97 | 2.51 |
| WDR4 | S390 | Hyper | 6.59 | 3.66 | 4.08 | 2.88 | 1.88 |
| KIF18B | S674 | Hyper | 14.60 | 2.01 | 3.24 | 3.78 | 2.73 |
| LARP7 | T264 | Hyper | 12.52 | 2.64 | 3.03 | 3.91 | 2.03 |
| TJP3 | S112 | Hyper | 21.02 | 2.85 | 2.18 | 2.45 | 2.43 |
| TJP3 | S319 | Hyper | 11.21 | 1.85 | 2.27 | 3.26 | 4.58 |
| TJP3 | S327 | Hyper | 8.53 | 2.06 | 1.95 | 3.26 | 6.50 |
| HSP90AB1 | S226 | Hyper | 6.04 | 3.36 | 2.84 | 2.68 | 1.71 |
| DOCK7 | S900 | Hyper | 4.13 | 1.65 | 3.60 | 3.48 | 2.42 |
| USP10 | S580 | Hyper | 3.66 | 1.81 | 2.65 | 2.99 | 1.67 |
| CHD2 | S1085 | Hyper | 7.88 | 1.99 | 1.74 | 41.41 | 12.06 |
| ESF1 | S153 | Hyper | 10.74 | 4.28 | 7.33 | 3.12 | 3.54 |
| ESF1 | S663 | Hyper | 6.66 | 3.02 | 3.24 | 3.78 | 1.74 |
| STIM1 | S257 | Hyper | 2.27 | 2.41 | 2.34 | 3.10 | 1.91 |
| LILRB4 | S318 | Hyper | 4.49 | 1.91 | 2.98 | 1.68 | 1.99 |
| LILRB4 | S319 | Hyper | 10.43 | 1.76 | 2.98 | 2.22 | 1.99 |

| | | | | | | | |
|---|---|---|---|---|---|---|---|
| RANGAP1 | S442 | Hyper | 11.83 | 4.22 | 4.64 | 4.20 | 2.23 |
| PBK | S59 | Hyper | 9.29 | 2.33 | 3.74 | 6.19 | 2.95 |
| ARHGAP27 | S466 | Hyper | 10.49 | 1.68 | 2.20 | 1.97 | 1.84 |
| ADNP | S98 | Hyper | 14.15 | 2.21 | 3.49 | 5.37 | 2.53 |
| JMJD1C | S470 | Hyper | 6.30 | 2.67 | 2.36 | 4.04 | 1.96 |
| CCAR1 | S682 | Hyper | 12.59 | 2.21 | 2.60 | 2.25 | 1.78 |
| SIPA1L1 | S1181 | Hyper | 5.57 | 2.25 | 2.18 | 2.76 | 2.40 |
| SIPA1L1 | S1249 | Hyper | 11.74 | 1.83 | 3.51 | 3.16 | 1.86 |
| SIPA1L1 | S1487 | Hyper | 6.05 | 2.30 | 2.94 | 3.25 | 2.10 |
| SIPA1L1 | S1507 | Hyper | 2.92 | 2.52 | 2.65 | 3.19 | 2.55 |
| NOLC1 | S518 | Hyper | 13.90 | 4.84 | 4.50 | 3.68 | 3.39 |
| NOLC1 | S573 | Hyper | 16.43 | 3.20 | 2.84 | 2.65 | 2.23 |
| C2CD5 | S284 | Hyper | 3.48 | 2.38 | 1.77 | 3.46 | 1.71 |
| C2CD5 | S662 | Hyper | 4.79 | 2.06 | 2.00 | 2.20 | 2.11 |
| GTF3C1 | S1068 | Hyper | 8.16 | 2.31 | 2.23 | 1.91 | 2.81 |
| GTF3C1 | T1873 | Hyper | 19.79 | 2.48 | 3.10 | 3.30 | 2.33 |
| SLC35B2 | S422 | Hyper | 6.11 | 2.24 | 3.31 | 2.16 | 2.32 |
| PHRF1 | S1360 | Hyper | 9.34 | 2.16 | 2.43 | 2.60 | 2.55 |
| PHRF1 | S1371 | Hyper | 6.62 | 2.24 | 2.46 | 2.60 | 3.42 |
| PHRF1 | S991 | Hyper | 7.60 | 1.80 | 2.46 | 3.49 | 1.72 |
| FARP1 | S920 | Hyper | 5.65 | 1.72 | 2.06 | 1.82 | 3.75 |
| NUP35 | S262 | Hyper | 4.71 | 2.86 | 6.51 | 2.29 | 2.07 |
| NUP35 | T291 | Hyper | 10.45 | 1.87 | 3.94 | 4.84 | 1.64 |
| CYFIP1 | T1234 | Hyper | 4.44 | 1.90 | 1.97 | 2.92 | 2.52 |
| TEX2 | S790 | Hyper | 6.54 | 1.77 | 1.59 | 2.29 | 2.16 |
| KLC4 | T162 | Hyper | 6.33 | 3.03 | 2.11 | 7.44 | 2.03 |
| EI24 | S304 | Hyper | 13.10 | 2.17 | 3.03 | 2.22 | 2.15 |
| DOCK1 | T1793 | Hyper | 2.15 | 1.57 | 1.79 | 2.88 | 1.57 |
| PHF3 | S1045 | Hyper | 8.23 | 2.10 | 2.34 | 1.79 | 1.61 |
| HSH2D | S137 | Hyper | 8.03 | 2.07 | 3.08 | 2.18 | 3.12 |
| KDM6A | T879 | Hyper | 5.90 | 2.31 | 2.36 | 2.53 | 2.06 |
| AKAP8L | S222 | Hyper | 8.74 | 2.40 | 2.30 | 2.33 | 1.73 |
| NOP14 | S148 | Hyper | 5.60 | 2.27 | 2.29 | 1.56 | 2.12 |
| CLK2 | S50 | Hyper | 8.63 | 2.38 | 3.72 | 2.95 | 2.25 |
| ZC3H18 | S67 | Hyper | 11.42 | 2.02 | 2.32 | 2.46 | 2.60 |
| ZC3H18 | S74 | Hyper | 10.98 | 2.35 | 2.25 | 2.06 | 3.44 |
| CHD4 | S1524 | Hyper | 7.12 | 2.21 | 2.43 | 1.87 | 1.88 |
| CHD4 | S1569 | Hyper | 17.95 | 2.61 | 2.66 | 3.02 | 1.76 |
| CHD4 | T1542 | Hyper | 6.34 | 1.67 | 2.16 | 1.75 | 2.27 |
| CHD4 | T1546 | Hyper | 8.01 | 1.72 | 2.97 | 2.19 | 2.27 |
| RPRD2 | S902 | Hyper | 12.93 | 1.62 | 2.53 | 4.09 | 2.47 |
| BAZ2A | S1768 | Hyper | 10.93 | 2.38 | 2.33 | 4.41 | 3.80 |
| GATAD2A | S100 | Hyper | 4.33 | 1.76 | 2.09 | 2.37 | 2.06 |

| | | | | | | | |
|---|---|---|---|---|---|---|---|
| BCLAF1 | S194 | Hyper | 6.05 | 3.03 | 2.82 | 2.72 | 2.41 |
| BCLAF1 | S196 | Hyper | 7.22 | 1.70 | 2.89 | 3.22 | 1.70 |
| BCLAF1 | S266 | Hyper | 9.83 | 2.67 | 3.36 | 3.07 | 2.65 |
| BCLAF1 | S288 | Hyper | 6.18 | 2.00 | 2.23 | 2.98 | 2.24 |
| BCLAF1 | S387 | Hyper | 11.08 | 1.92 | 2.31 | 2.93 | 3.13 |
| BCLAF1 | S395 | Hyper | 6.60 | 2.37 | 2.56 | 2.21 | 1.65 |
| BCLAF1 | S494 | Hyper | 6.04 | 2.17 | 2.46 | 2.48 | 1.70 |
| BCLAF1 | S510 | Hyper | 6.30 | 2.28 | 2.39 | 3.01 | 1.67 |
| BCLAF1 | S758 | Hyper | 4.44 | 2.33 | 3.42 | 2.97 | 2.96 |
| BCLAF1 | T400 | Hyper | 8.81 | 2.58 | 2.58 | 2.47 | 1.73 |
| BCLAF1 | T403 | Hyper | 10.08 | 2.51 | 2.85 | 2.37 | 1.65 |
| WDR75 | S715 | Hyper | 7.56 | 2.89 | 3.54 | 3.31 | 1.94 |
| WDR75 | S718 | Hyper | 7.56 | 3.09 | 4.75 | 3.31 | 1.90 |
| WDR75 | S732 | Hyper | 10.52 | 3.45 | 3.01 | 4.45 | 13.44 |
| TUBA1C | S118 | Hyper | 10.57 | 2.49 | 4.71 | 3.94 | 6.42 |
| HEXIM2 | T54 | Hyper | 7.67 | 1.67 | 2.46 | 2.07 | 2.21 |
| SRRM1 | S260 | Hyper | 6.77 | 1.99 | 2.15 | 1.96 | 1.62 |
| SRRM1 | S391 | Hyper | 5.51 | 2.02 | 1.97 | 4.69 | 2.73 |
| SRRM1 | S393 | Hyper | 5.51 | 2.18 | 2.17 | 5.09 | 2.48 |
| SRRM1 | S463 | Hyper | 7.44 | 2.08 | 2.00 | 3.76 | 1.91 |
| SRRM1 | S609 | Hyper | 4.72 | 1.76 | 3.58 | 2.53 | 2.45 |
| SRRM1 | S617 | Hyper | 2.11 | 1.84 | 3.48 | 2.88 | 1.94 |
| SRRM1 | S628 | Hyper | 6.22 | 1.72 | 2.20 | 1.84 | 2.01 |
| SRRM1 | S650 | Hyper | 9.09 | 2.18 | 2.46 | 2.59 | 2.10 |
| SRRM1 | S750 | Hyper | 9.65 | 2.12 | 2.33 | 2.09 | 1.88 |
| SRRM1 | S752 | Hyper | 5.06 | 2.21 | 2.22 | 2.08 | 1.98 |
| SRRM1 | S768 | Hyper | 8.43 | 2.02 | 3.28 | 2.23 | 2.38 |
| SRRM1 | S781 | Hyper | 6.58 | 1.92 | 2.63 | 2.12 | 5.02 |
| SRRM1 | S787 | Hyper | 6.48 | 1.83 | 2.02 | 2.12 | 5.20 |
| SRRM1 | S809 | Hyper | 4.85 | 3.11 | 2.30 | 3.05 | 2.30 |
| SRRM1 | S886 | Hyper | 6.55 | 2.34 | 2.42 | 2.89 | 1.80 |
| SRRM1 | T220 | Hyper | 6.59 | 2.03 | 2.33 | 2.63 | 1.69 |
| SRRM1 | T416 | Hyper | 18.11 | 3.08 | 4.35 | 8.20 | 1.89 |
| SRRM1 | T584 | Hyper | 8.79 | 2.68 | 2.70 | 2.42 | 3.65 |
| SRRM1 | T586 | Hyper | 8.79 | 2.58 | 2.49 | 2.47 | 2.35 |
| SRRM1 | T790 | Hyper | 6.13 | 1.93 | 2.02 | 2.12 | 5.38 |
| THUMPD1 | S88 | Hyper | 6.75 | 2.35 | 2.08 | 3.21 | 2.03 |
| ZNF687 | S1057 | Hyper | 7.54 | 2.19 | 2.99 | 4.01 | 1.90 |
| ZNF687 | S1106 | Hyper | 7.75 | 3.33 | 1.78 | 5.23 | 2.11 |
| ZNF687 | S140 | Hyper | 8.22 | 1.80 | 2.68 | 2.86 | 1.93 |
| ZNF687 | S253 | Hyper | 9.99 | 2.38 | 3.36 | 2.20 | 3.02 |
| ZNF687 | S266 | Hyper | 10.52 | 2.18 | 3.81 | 1.92 | 2.61 |
| ZNF687 | S271 | Hyper | 10.52 | 2.24 | 3.81 | 2.80 | 2.85 |

| | | | | | | | |
|---|---|---|---|---|---|---|---|
| ZNF687 | S374 | Hyper | 12.45 | 2.42 | 3.22 | 2.10 | 1.91 |
| ZNF687 | T377 | Hyper | 6.23 | 1.90 | 2.51 | 2.32 | 3.01 |
| ZNF687 | T432 | Hyper | 3.19 | 1.71 | 3.00 | 3.98 | 2.15 |
| BRD1 | S945 | Hyper | 6.95 | 3.93 | 2.93 | 3.36 | 2.57 |
| CIC | S2306 | Hyper | 5.82 | 1.62 | 1.79 | 3.13 | 1.93 |
| CIC | S2318 | Hyper | 5.26 | 1.95 | 2.31 | 4.23 | 1.93 |
| CIC | T2307 | Hyper | 4.69 | 1.77 | 1.86 | 2.47 | 1.93 |
| CIC | T2317 | Hyper | 10.95 | 1.65 | 2.48 | 2.62 | 1.93 |
| CAD | S1796 | Hyper | 4.33 | 2.44 | 3.79 | 2.17 | 1.91 |
| TICRR | S598 | Hyper | 11.15 | 2.53 | 4.94 | 2.49 | 2.55 |
| ARHGAP39 | S407 | Hyper | 7.89 | 2.09 | 2.59 | 2.94 | 1.87 |
| DOK3 | S308 | Hyper | 9.25 | 1.74 | 1.98 | 2.53 | 1.75 |
| MAP1S | S449 | Hyper | 3.28 | 1.96 | 1.78 | 3.44 | 1.65 |
| MAP1S | S737 | Hyper | 3.83 | 1.72 | 1.80 | 1.95 | 4.82 |
| PCYT1A | S323 | Hyper | 4.10 | 2.51 | 3.58 | 1.87 | 3.91 |
| KDM5B | S1492 | Hyper | 9.04 | 1.68 | 6.39 | 3.39 | 1.85 |
| CLIC6 | S303 | Hyper | 5.08 | 2.04 | 2.45 | 3.33 | 1.75 |
| XRN2 | S526 | Hyper | 22.48 | 2.72 | 2.35 | 2.20 | 2.02 |
| XRN2 | S577 | Hyper | 5.89 | 2.52 | 2.79 | 1.73 | 1.94 |
| XRN2 | S579 | Hyper | 6.67 | 2.74 | 2.44 | 2.70 | 2.12 |
| HNRNPK | S284 | Hyper | 4.88 | 1.81 | 2.34 | 2.49 | 1.69 |
| DNMT1 | S143 | Hyper | 7.51 | 2.87 | 2.78 | 2.74 | 2.76 |
| DNMT1 | S154 | Hyper | 16.19 | 2.91 | 2.78 | 4.94 | 3.91 |
| DNMT1 | S714 | Hyper | 9.63 | 2.46 | 4.11 | 2.83 | 2.70 |
| NFX1 | S150 | Hyper | 9.46 | 1.74 | 1.97 | 2.59 | 2.76 |
| EIF2A | S481 | Hyper | 7.66 | 2.59 | 3.31 | 3.81 | 2.24 |
| HMGA1 | S36 | Hyper | 6.23 | 2.44 | 2.23 | 3.70 | 2.34 |
| HMGA1 | S44 | Hyper | 5.49 | 4.24 | 2.66 | 2.55 | 1.97 |
| HMGA1 | T39 | Hyper | 6.39 | 3.23 | 2.49 | 1.95 | 2.53 |
| HMGA1 | T53 | Hyper | 8.06 | 3.73 | 5.58 | 3.27 | 2.78 |
| ZC3H11A | S171 | Hyper | 8.60 | 1.95 | 2.69 | 4.17 | 2.06 |
| ZC3H11A | S758 | Hyper | 4.37 | 3.42 | 2.67 | 1.89 | 1.76 |
| ZC3H11A | S759 | Hyper | 5.27 | 2.43 | 2.67 | 3.51 | 1.76 |
| NUP88 | S517 | Hyper | 10.14 | 2.61 | 4.23 | 2.71 | 2.55 |
| CDK1 | T161 | Hyper | 12.53 | 1.73 | 5.53 | 2.33 | 2.02 |
| GPS1 | S536 | Hyper | 3.01 | 1.94 | 2.80 | 4.55 | 2.02 |
| GPS1 | S551 | Hyper | 7.89 | 2.08 | 1.90 | 4.50 | 2.04 |
| GPS1 | T547 | Hyper | 7.89 | 1.82 | 2.18 | 1.75 | 1.98 |
| SLC4A7 | S288 | Hyper | 8.73 | 2.02 | 2.53 | 5.32 | 2.70 |
| SLC4A7 | S292 | Hyper | 4.55 | 2.17 | 4.50 | 3.35 | 2.44 |
| THRAP3 | S243 | Hyper | 3.25 | 2.15 | 2.36 | 1.84 | 1.80 |
| THRAP3 | S248 | Hyper | 5.26 | 2.13 | 2.77 | 3.01 | 1.66 |
| THRAP3 | S253 | Hyper | 5.39 | 2.34 | 1.89 | 3.07 | 1.66 |

| | | | | | | | |
|---|---|---|---|---|---|---|---|
| THRAP3 | S315 | Hyper | 6.96 | 1.95 | 2.20 | 2.27 | 5.02 |
| THRAP3 | S320 | Hyper | 6.96 | 1.84 | 2.29 | 1.96 | 5.02 |
| NCOA2 | S29 | Hyper | 5.62 | 1.89 | 2.13 | 2.19 | 1.63 |
| AHCTF1 | S1222 | Hyper | 7.34 | 2.24 | 3.16 | 2.39 | 1.95 |
| ADAT1 | S42 | Hyper | 4.28 | 2.62 | 3.45 | 2.72 | 2.07 |
| WASHC2C | S158 | Hyper | 4.86 | 1.73 | 1.64 | 3.96 | 1.73 |
| TIMELESS | S1120 | Hyper | 5.08 | 2.41 | 1.78 | 1.68 | 2.35 |
| TIMELESS | S1148 | Hyper | 5.33 | 2.52 | 4.51 | 4.60 | 1.91 |
| HELZ | S1318 | Hyper | 13.03 | 1.93 | 2.95 | 3.47 | 1.94 |
| HELZ | T1164 | Hyper | 5.77 | 1.61 | 1.75 | 2.54 | 2.02 |
| SNX27 | S51 | Hyper | 3.82 | 2.43 | 2.57 | 1.87 | 1.86 |
| CCDC97 | S192 | Hyper | 4.56 | 1.58 | 2.33 | 4.62 | 1.85 |
| CTR9 | S1063 | Hyper | 12.38 | 1.96 | 3.25 | 5.50 | 2.19 |
| CTR9 | S917 | Hyper | 27.47 | 2.62 | 5.39 | 2.21 | 4.70 |
| CTR9 | T951 | Hyper | 7.38 | 2.23 | 2.46 | 2.60 | 2.16 |
| PPP1R35 | S52 | Hyper | 4.29 | 1.72 | 1.89 | 3.87 | 1.86 |
| FRA10AC1 | S278 | Hyper | 5.69 | 2.17 | 1.76 | 2.15 | 1.90 |
| SETD2 | S570 | Hyper | 11.65 | 1.87 | 1.87 | 2.92 | 3.10 |
| PBRM1 | S522 | Hyper | 5.12 | 1.99 | 2.49 | 3.39 | 2.14 |
| PBRM1 | S526 | Hyper | 5.12 | 1.57 | 2.13 | 2.31 | 1.73 |
| YEATS2 | S627 | Hyper | 6.06 | 1.89 | 2.68 | 3.44 | 2.20 |
| SETX | S1663 | Hyper | 4.88 | 1.90 | 2.29 | 2.08 | 1.92 |
| TRAPPC10 | S736 | Hyper | 11.43 | 2.41 | 2.87 | 2.95 | 1.82 |
| ELAVL1 | S202 | Hyper | 6.24 | 1.64 | 2.23 | 2.71 | 1.83 |
| HDAC2 | S422 | Hyper | 5.80 | 2.13 | 2.51 | 2.09 | 2.85 |
| EIF3E | S399 | Hyper | 6.20 | 1.87 | 3.12 | 2.36 | 2.12 |
| ARVCF | S343 | Hyper | 3.43 | 2.61 | 2.24 | 2.21 | 2.42 |
| ATP2B1 | S1182 | Hyper | 7.29 | 1.81 | 2.00 | 9.28 | 2.13 |
| CAV1 | S37 | Hypo | 0.46 | 0.39 | 0.15 | 0.09 | 0.21 |
| CAV1 | Y42 | Hypo | 0.50 | 0.49 | 0.36 | 0.26 | 0.24 |
| DES | S28 | Hypo | 0.39 | 0.48 | 0.25 | 0.26 | 0.50 |
| DES | S32 | Hypo | 0.34 | 0.34 | 0.00 | 0.26 | 0.28 |
| DSG2 | S700 | Hyper | 9.59 | 2.33 | 3.75 | 3.32 | 4.70 |
| DSG2 | S701 | Hyper | 14.71 | 2.33 | 7.35 | 4.50 | 3.87 |
| EEF2 | T59 | Hyper | 5.35 | 2.38 | 2.64 | 2.85 | 1.91 |
| ETV6 | S22 | Hyper | 2.31 | 1.84 | 1.64 | 6.91 | 2.22 |
| FKBP3 | S36 | Hyper | 4.33 | 1.74 | 2.85 | 2.52 | 4.36 |
| RBMX | S208 | Hyper | 5.08 | 2.32 | 3.12 | 2.51 | 1.67 |
| RBMX | S58 | Hyper | 6.96 | 1.79 | 2.52 | 2.08 | 1.73 |
| HSPA4 | S647 | Hyper | 12.13 | 1.79 | 1.94 | 2.98 | 1.82 |
| HSPD1 | S70 | Hyper | 7.36 | 3.22 | 3.63 | 1.95 | 1.86 |
| ITPR3 | S2670 | Hyper | 3.05 | 1.94 | 1.66 | 2.38 | 1.87 |
| KRT19 | S14 | Hyper | 4.65 | 1.57 | 2.55 | 2.73 | 1.96 |

| | | | | | | | |
|---|---|---|---|---|---|---|---|
| LIG3 | S210 | Hyper | 7.38 | 2.22 | 2.77 | 2.84 | 2.00 |
| LSP1 | S177 | Hyper | 7.28 | 1.68 | 1.97 | 2.53 | 2.13 |
| LSP1 | S188 | Hyper | 8.93 | 1.75 | 2.00 | 2.53 | 2.04 |
| LSP1 | S189 | Hyper | 9.40 | 1.82 | 2.59 | 2.12 | 1.84 |
| LSP1 | T175 | Hyper | 5.30 | 1.83 | 1.76 | 253.06 | 1.94 |
| MARCKS | S145 | Hyper | 8.47 | 2.08 | 2.63 | 2.50 | 2.30 |
| MARCKS | T150 | Hyper | 8.02 | 2.08 | 2.08 | 2.11 | 1.85 |
| MKI67 | S1131 | Hyper | 6.91 | 3.76 | 3.46 | 2.60 | 2.42 |
| MKI67 | S2223 | Hyper | 12.63 | 2.88 | 3.74 | 3.54 | 2.15 |
| MKI67 | S357 | Hyper | 33.29 | 3.02 | 6.39 | 3.24 | 2.79 |
| MKI67 | S374 | Hyper | 12.51 | 1.82 | 4.84 | 5.52 | 3.69 |
| MKI67 | T1923 | Hyper | 5.43 | 2.21 | 5.69 | 2.71 | 2.41 |
| OSBP | S198 | Hyper | 5.58 | 1.96 | 1.87 | 2.34 | 2.25 |
| OSBP | S382 | Hyper | 3.30 | 1.97 | 2.36 | 1.66 | 3.63 |
| OSBP | S386 | Hyper | 6.26 | 1.94 | 3.19 | 2.15 | 1.87 |
| OSBP | S389 | Hyper | 6.48 | 2.40 | 3.07 | 2.50 | 2.13 |
| PGAM1 | S31 | Hyper | 4.31 | 2.17 | 3.98 | 2.70 | 2.22 |
| PTBP1 | S459 | Hyper | 5.20 | 2.65 | 3.21 | 2.12 | 2.69 |
| RBBP8 | S163 | Hyper | 2.82 | 1.74 | 2.29 | 8.65 | 1.86 |
| SGTA | T81 | Hyper | 6.10 | 2.17 | 2.02 | 2.01 | 2.38 |
| SLC9A1 | S605 | Hyper | 2.83 | 1.67 | 2.00 | 2.41 | 2.85 |
| SMARCC1 | S310 | Hyper | 4.42 | 2.60 | 3.42 | 4.18 | 2.39 |
| SNRNP70 | S226 | Hyper | 13.54 | 2.28 | 2.85 | 4.18 | 1.81 |
| SP100 | S231 | Hyper | 8.80 | 2.48 | 2.12 | 6.38 | 2.06 |
| SP100 | S409 | Hyper | 5.94 | 2.36 | 3.43 | 2.17 | 1.84 |
| SP100 | S453 | Hyper | 4.51 | 1.75 | 2.57 | 2.97 | 1.72 |
| SPAG1 | S418 | Hyper | 6.38 | 3.11 | 5.06 | 2.33 | 3.50 |
| SRPK1 | S311 | Hyper | 8.96 | 2.55 | 4.04 | 3.50 | 2.89 |
| SSRP1 | S444 | Hyper | 8.31 | 2.24 | 2.55 | 5.28 | 1.52 |
| SSRP1 | Y438 | Hyper | 6.50 | 2.35 | 1.82 | 5.31 | 1.52 |
| SEC62 | S315 | Hyper | 19.51 | 2.33 | 2.65 | 2.48 | 1.66 |
| TPR | S1676 | Hyper | 9.48 | 2.99 | 6.63 | 4.37 | 2.30 |
| VDAC1 | S104 | Hyper | 6.46 | 3.20 | 3.57 | 2.58 | 3.29 |
| CLIP2 | S207 | Hyper | 10.31 | 1.71 | 1.60 | 3.19 | 2.20 |
| WEE1 | S165 | Hyper | 10.87 | 2.52 | 3.60 | 3.28 | 2.33 |
| KMT2D | S3130 | Hyper | 8.31 | 1.83 | 2.35 | 2.60 | 1.72 |
| AXIN1 | S486 | Hyper | 9.65 | 1.93 | 2.76 | 4.18 | 6.52 |
| AXIN1 | S493 | Hyper | 8.14 | 2.10 | 2.76 | 5.84 | 4.30 |
| YBX3 | S203 | Hyper | 3.84 | 1.64 | 3.66 | 3.44 | 2.69 |
| YBX3 | S204 | Hyper | 3.26 | 1.64 | 3.21 | 4.25 | 2.69 |
| DENR | S73 | Hyper | 6.85 | 2.38 | 4.05 | 1.92 | 2.33 |
| TRIM24 | S710 | Hyper | 5.42 | 2.19 | 2.40 | 3.13 | 2.96 |
| TRIM24 | S777 | Hyper | 8.34 | 2.91 | 2.62 | 3.34 | 1.80 |

| | | | | | | | |
|---|---|---|---|---|---|---|---|
| TRIM24 | S991 | Hyper | 9.89 | 2.46 | 2.56 | 3.45 | 2.66 |
| TRIM24 | S994 | Hyper | 11.79 | 3.02 | 2.67 | 2.90 | 2.98 |
| SQSTM1 | S152 | Hyper | 5.33 | 1.79 | 3.95 | 2.59 | 2.11 |
| SQSTM1 | S24 | Hyper | 3.55 | 2.12 | 2.05 | 2.20 | 1.75 |
| SQSTM1 | S272 | Hyper | 6.07 | 2.49 | 3.97 | 2.28 | 2.17 |
| PRPF4B | S144 | Hyper | 4.73 | 1.95 | 2.18 | 3.00 | 2.50 |
| PRPF4B | S20 | Hyper | 12.47 | 2.21 | 2.66 | 2.17 | 1.85 |
| PRPF4B | S23 | Hyper | 11.86 | 2.27 | 2.88 | 2.30 | 1.80 |
| PRPF4B | S437 | Hyper | 2.86 | 2.01 | 2.51 | 4.01 | 2.47 |
| ST13 | S75 | Hyper | 2.54 | 2.46 | 2.50 | 14.73 | 1.76 |
| UBE2M | S28 | Hyper | 10.94 | 3.86 | 2.76 | 2.21 | 2.77 |
| EIF4EBP1 | T70 | Hyper | 3.11 | 1.78 | 1.74 | 5.81 | 2.24 |
| TARBP2 | S131 | Hyper | 5.75 | 1.99 | 4.03 | 1.90 | 3.47 |
| ARHGAP1 | S51 | Hyper | 5.40 | 2.46 | 1.97 | 1.89 | 1.66 |
| ASPH | S22 | Hyper | 4.35 | 3.16 | 3.39 | 3.90 | 3.47 |
| DAP | S51 | Hyper | 5.67 | 2.01 | 2.88 | 2.95 | 1.72 |
| DSP | S178 | Hyper | 4.76 | 1.98 | 3.94 | 1.76 | 2.54 |
| DSP | S2820 | Hyper | 6.73 | 1.72 | 3.53 | 1.96 | 2.91 |
| DSP | S2821 | Hyper | 6.66 | 1.72 | 2.47 | 2.48 | 2.17 |
| DSP | S2825 | Hyper | 6.78 | 1.81 | 2.29 | 2.36 | 2.91 |
| BPTF | S1231 | Hyper | 3.13 | 1.99 | 3.02 | 2.14 | 1.96 |
| BPTF | S2370 | Hyper | 8.28 | 1.99 | 2.39 | 2.62 | 1.78 |
| BPTF | S572 | Hyper | 10.69 | 1.76 | 2.26 | 2.85 | 2.61 |
| GOLGA2 | S66 | Hyper | 7.54 | 2.85 | 7.36 | 8.56 | 2.32 |
| MCM2 | S139 | Hyper | 6.95 | 2.69 | 5.32 | 2.64 | 2.06 |
| YBX1 | S165 | Hyper | 6.46 | 2.22 | 2.58 | 2.40 | 2.76 |
| YBX1 | S174 | Hyper | 6.46 | 2.06 | 4.11 | 2.40 | 1.70 |
| TRA2B | S264 | Hyper | 3.27 | 3.47 | 2.71 | 3.56 | 1.95 |
| TRA2B | S266 | Hyper | 5.32 | 3.70 | 2.63 | 2.22 | 3.07 |
| PRRC2A | S1089 | Hyper | 6.77 | 2.73 | 2.74 | 2.11 | 2.82 |
| PRRC2A | S1092 | Hyper | 13.00 | 2.93 | 4.46 | 1.81 | 2.27 |
| PRRC2A | S350 | Hyper | 3.75 | 1.99 | 2.52 | 2.69 | 2.41 |
| PRRC2A | S380 | Hyper | 8.28 | 2.23 | 2.61 | 2.67 | 1.74 |
| PRRC2A | T610 | Hyper | 6.63 | 1.66 | 2.87 | 2.12 | 1.91 |
| CAVIN2 | S293 | Hypo | 0.23 | 0.26 | 0.29 | 0.40 | 0.21 |
| PRPF3 | S619 | Hyper | 9.02 | 2.53 | 3.37 | 2.16 | 1.81 |
| SCAF11 | S413 | Hyper | 5.46 | 1.73 | 2.49 | 1.67 | 1.73 |
| SCAF11 | S776 | Hyper | 5.84 | 1.98 | 2.42 | 3.64 | 1.88 |
| DDX21 | S121 | Hyper | 2.77 | 4.98 | 4.56 | 5.28 | 3.19 |
| DDX21 | S173 | Hyper | 9.94 | 3.19 | 3.48 | 4.99 | 3.13 |
| DLG5 | S1000 | Hyper | 8.83 | 1.72 | 2.80 | 2.26 | 1.83 |
| MED1 | S1401 | Hyper | 11.00 | 2.24 | 2.47 | 3.99 | 2.16 |
| PPIG | S696 | Hyper | 4.63 | 1.94 | 4.18 | 3.13 | 2.77 |

| | | | | | | | |
|---|---|---|---|---|---|---|---|
| PPIG | S745 | Hyper | 8.50 | 1.96 | 2.48 | 2.24 | 2.48 |
| SYMPK | S1243 | Hyper | 18.71 | 1.82 | 3.02 | 2.57 | 2.64 |
| SYMPK | S1259 | Hyper | 12.83 | 2.04 | 2.65 | 2.92 | 2.64 |
| HDAC1 | S421 | Hyper | 6.40 | 1.96 | 2.84 | 2.23 | 2.77 |
| HDAC1 | S423 | Hyper | 8.60 | 2.77 | 2.07 | 2.86 | 4.12 |
| ABCC1 | S930 | Hyper | 9.14 | 2.87 | 3.49 | 2.48 | 1.83 |
| TRIM25 | S100 | Hyper | 5.57 | 1.74 | 2.60 | 2.89 | 2.18 |
| ZRSR2 | S384 | Hyper | 7.88 | 2.65 | 2.05 | 2.25 | 1.70 |
| AKAP12 | S286 | Hypo | 0.23 | 0.29 | 0.40 | 0.28 | 0.15 |
| AKAP12 | S290 | Hypo | 0.30 | 0.56 | 0.23 | 0.30 | 0.39 |
| MED12 | S635 | Hyper | 11.23 | 2.23 | 2.44 | 2.29 | 1.64 |
| BCL3 | S374 | Hyper | 6.53 | 2.17 | 4.06 | 3.24 | 3.58 |
| CTTN | S418 | Hyper | 5.48 | 2.86 | 2.23 | 1.62 | 1.89 |
| LMO7 | S1182 | Hyper | 4.63 | 1.78 | 3.52 | 1.73 | 2.77 |
| NCL | S34 | Hyper | 4.75 | 2.59 | 2.80 | 4.75 | 2.35 |
| NCL | S41 | Hyper | 6.47 | 2.48 | 2.43 | 4.92 | 2.30 |
| NCL | S42 | Hyper | 6.39 | 2.48 | 2.37 | 4.57 | 2.30 |
| NCL | S67 | Hyper | 8.27 | 4.19 | 4.61 | 2.46 | 2.70 |
| NCL | T76 | Hyper | 6.98 | 2.49 | 4.81 | 4.08 | 2.83 |
| ARHGEF5 | S983 | Hyper | 8.31 | 1.78 | 3.27 | 4.04 | 4.23 |
| CCDC6 | S240 | Hyper | 2.00 | 1.89 | 2.01 | 1.92 | 1.74 |
| CCDC6 | S52 | Hyper | 6.18 | 3.01 | 3.47 | 7.33 | 2.77 |
| ZRANB2 | S307 | Hyper | 9.22 | 2.24 | 2.00 | 1.87 | 2.47 |
| SH2D3A | S180 | Hyper | 5.53 | 2.20 | 2.85 | 2.33 | 4.81 |
| CFL1 | T25 | Hyper | 6.16 | 2.09 | 2.50 | 2.40 | 1.79 |
| HSF1 | S307 | Hyper | 8.27 | 2.09 | 2.70 | 2.36 | 1.86 |
| LAD1 | S177 | Hyper | 4.19 | 2.33 | 6.06 | 3.72 | 4.14 |
| LAD1 | S64 | Hyper | 4.04 | 1.84 | 3.49 | 2.22 | 2.04 |
| LMNB1 | S393 | Hyper | 1.75 | 2.36 | 2.65 | 5.34 | 1.74 |
| TCF20 | S574 | Hyper | 32.06 | 2.39 | 3.59 | 2.38 | 2.09 |
| TCF20 | S640 | Hyper | 8.31 | 2.28 | 2.38 | 2.61 | 2.08 |
| CEBPZ | S629 | Hyper | 11.57 | 2.81 | 3.65 | 4.85 | 2.53 |
| RBM6 | S1025 | Hyper | 3.99 | 1.72 | 2.03 | 2.85 | 2.06 |
| RBM6 | S891 | Hyper | 10.50 | 2.12 | 2.42 | 3.51 | 1.99 |
| AKAP8 | S328 | Hyper | 9.62 | 2.07 | 2.68 | 1.79 | 1.88 |
| AKAP8 | S339 | Hyper | 7.01 | 3.16 | 2.54 | 4.04 | 2.05 |
| STUB1 | S19 | Hyper | 4.70 | 2.24 | 3.84 | 3.35 | 1.78 |
| STUB1 | S23 | Hyper | 5.09 | 2.65 | 2.94 | 3.35 | 1.78 |
| MCM4 | S120 | Hyper | 15.79 | 2.63 | 4.46 | 2.64 | 3.71 |
| MCM6 | S762 | Hyper | 7.58 | 2.55 | 4.83 | 1.83 | 2.00 |
| MLLT1 | S475 | Hyper | 5.97 | 2.96 | 2.43 | 4.60 | 1.55 |
| ORC2 | T226 | Hyper | 17.23 | 2.16 | 6.82 | 4.02 | 3.26 |
| PRKAB1 | S96 | Hyper | 5.44 | 1.95 | 2.25 | 1.80 | 2.64 |

| | | | | | | | | |
|---|---|---|---|---|---|---|---|---|
| RANBP2 | S781 | Hyper | 9.11 | 2.93 | 3.31 | 2.74 | 4.15 |
| RANBP2 | T1396 | Hyper | 7.28 | 1.90 | 3.39 | 2.92 | 3.03 |
| RANBP2 | T2153 | Hyper | 6.67 | 2.41 | 3.69 | 3.09 | 2.39 |
| RANBP2 | T799 | Hyper | 9.71 | 2.86 | 4.36 | 3.18 | 3.13 |
| SRSF6 | S303 | Hyper | 7.68 | 1.88 | 2.42 | 2.00 | 1.87 |
| XRCC1 | S241 | Hyper | 8.96 | 3.23 | 8.26 | 5.45 | 3.30 |
| XRCC1 | S447 | Hyper | 7.62 | 2.36 | 3.44 | 3.67 | 1.81 |
| XRCC1 | T453 | Hyper | 8.43 | 2.36 | 3.25 | 3.35 | 3.65 |
| PGRMC2 | T235 | Hyper | 3.67 | 1.69 | 1.72 | 1.73 | 2.11 |
| TACC3 | S434 | Hyper | 16.26 | 1.87 | 3.75 | 2.02 | 1.85 |
| NOP56 | S519 | Hyper | 5.74 | 2.72 | 2.32 | 2.08 | 1.93 |
| ANP32B | T15 | Hyper | 7.43 | 2.47 | 2.45 | 4.35 | 2.02 |
| ARFGEF1 | S52 | Hyper | 7.46 | 2.21 | 3.20 | 4.64 | 2.72 |
| RGS14 | S288 | Hyper | 4.35 | 2.65 | 1.68 | 1.95 | 1.79 |
| SLC12A7 | T973 | Hyper | 4.38 | 1.52 | 1.99 | 1.93 | 1.95 |
| NUDC | S139 | Hyper | 11.25 | 3.08 | 3.36 | 2.08 | 1.94 |
| NUDC | T145 | Hyper | 6.17 | 2.91 | 3.18 | 3.89 | 1.92 |
| KIF1C | S676 | Hyper | 7.75 | 1.68 | 2.20 | 1.85 | 2.34 |
| SRCAP | S2430 | Hyper | 11.71 | 1.93 | 3.05 | 3.09 | 3.28 |
| SRCAP | S2869 | Hyper | 1.96 | 2.28 | 3.33 | 4.61 | 1.92 |
| SUB1 | S118 | Hyper | 5.39 | 1.84 | 2.85 | 1.92 | 1.66 |
| SUB1 | S15 | Hyper | 4.49 | 2.51 | 3.33 | 2.53 | 2.51 |
| SUB1 | S19 | Hyper | 7.11 | 3.23 | 3.44 | 1.80 | 2.07 |
| AKAP13 | S1686 | Hyper | 2.48 | 1.87 | 2.71 | 9.36 | 2.81 |
| AKAP13 | S983 | Hyper | 6.72 | 1.91 | 3.49 | 1.84 | 1.71 |
| AKAP13 | T953 | Hyper | 4.39 | 1.93 | 2.10 | 2.84 | 1.66 |
| SF3B2 | S309 | Hyper | 6.74 | 2.34 | 2.68 | 1.94 | 2.89 |
| SF3B2 | S347 | Hyper | 20.58 | 2.58 | 3.50 | 2.79 | 2.16 |
| SF3B2 | T311 | Hyper | 5.95 | 2.34 | 3.03 | 3.90 | 2.89 |
| ZFHX3 | S2230 | Hyper | 6.33 | 1.88 | 1.87 | 2.54 | 1.69 |
| TMEM115 | T329 | Hyper | 9.99 | 2.15 | 3.85 | 3.20 | 2.29 |
| KATNA1 | S170 | Hyper | 5.21 | 2.17 | 1.88 | 1.96 | 1.84 |
| DDX20 | T705 | Hyper | 5.42 | 2.29 | 3.00 | 1.81 | 1.80 |
| SEC63 | T537 | Hyper | 9.00 | 2.50 | 2.59 | 3.13 | 2.75 |
| PNKP | S114 | Hyper | 3.75 | 4.16 | 1.73 | 2.03 | 1.93 |
| CBX3 | S97 | Hyper | 2.51 | 2.40 | 2.80 | 3.33 | 2.23 |
| OGFR | S378 | Hyper | 4.60 | 2.16 | 2.27 | 2.48 | 1.70 |
| DDX42 | S185 | Hyper | 6.12 | 2.10 | 2.53 | 2.25 | 1.59 |
| DDX42 | Y183 | Hyper | 7.37 | 1.96 | 2.59 | 3.21 | 2.23 |
| CD2AP | S510 | Hyper | 11.03 | 2.47 | 3.84 | 2.61 | 3.87 |
| CD2AP | S514 | Hyper | 10.53 | 2.67 | 4.89 | 2.49 | 2.99 |
| CD2AP | T512 | Hyper | 8.73 | 2.85 | 4.92 | 4.51 | 3.31 |
| AATF | S321 | Hyper | 5.25 | 2.65 | 3.48 | 2.15 | 2.75 |

| | | | | | | | | |
|---|---|---|---|---|---|---|---|---|
| RABGAP1 | S56 | Hyper | 6.11 | 1.90 | 2.36 | 4.79 | 3.15 |
| CAVIN1 | S171 | Hypo | 0.17 | 0.18 | 0.18 | 0.10 | 0.32 |
| KDM2A | S718 | Hyper | 4.65 | 1.64 | 2.14 | 2.17 | 2.25 |
| SF3B1 | S488 | Hyper | 8.66 | 2.78 | 2.92 | 2.35 | 1.96 |
| SF3B1 | T350 | Hyper | 6.39 | 2.18 | 1.93 | 2.12 | 2.01 |
| PSD4 | S134 | Hyper | 6.57 | 1.81 | 1.97 | 2.59 | 3.08 |
| PSD4 | S143 | Hyper | 6.93 | 1.97 | 2.19 | 2.61 | 2.70 |
| VPS4A | S95 | Hyper | 4.06 | 1.66 | 2.09 | 1.98 | 2.15 |
| VPS4A | S97 | Hyper | 4.18 | 1.90 | 2.09 | 2.82 | 1.77 |
| VPS4A | S99 | Hyper | 4.18 | 1.74 | 2.13 | 4.39 | 2.37 |
| TRA2A | S260 | Hyper | 3.27 | 3.47 | 2.71 | 3.56 | 1.95 |
| TRA2A | S262 | Hyper | 5.32 | 3.70 | 2.63 | 2.22 | 3.07 |
| PRRC2B | S563 | Hyper | 4.68 | 1.84 | 2.13 | 1.66 | 2.51 |
| PAXBP1 | S262 | Hyper | 4.89 | 2.37 | 2.60 | 4.81 | 1.80 |
| ANAPC2 | S314 | Hyper | 5.48 | 1.86 | 2.80 | 2.06 | 2.91 |
| BAZ1A | T731 | Hyper | 10.06 | 1.95 | 2.48 | 2.70 | 2.06 |
| NOB1 | S201 | Hyper | 8.36 | 2.97 | 2.59 | 2.11 | 2.08 |
| ATAD2 | S342 | Hyper | 16.94 | 4.08 | 15.55 | 3.25 | 4.04 |
| ZC3H7A | S738 | Hyper | 4.75 | 2.11 | 1.75 | 2.22 | 2.84 |
| AP2A1 | T653 | Hyper | 4.14 | 1.67 | 1.82 | 2.42 | 1.88 |
| CELSR1 | S2761 | Hyper | 11.46 | 2.14 | 1.96 | 2.89 | 2.08 |
| CELSR1 | S2764 | Hyper | 9.57 | 2.14 | 1.70 | 2.89 | 2.49 |
| CELSR1 | S2867 | Hyper | 5.88 | 2.78 | 1.86 | 2.28 | 2.67 |
| ZNF318 | S1856 | Hyper | 11.49 | 2.28 | 2.74 | 3.15 | 1.81 |
| PELP1 | S531 | Hyper | 10.30 | 2.55 | 2.73 | 3.71 | 1.95 |
| ERO1A | S106 | Hyper | 4.93 | 2.15 | 4.75 | 2.27 | 2.30 |
| DNTTIP2 | S117 | Hyper | 3.31 | 1.70 | 2.57 | 2.80 | 1.74 |
| DNTTIP2 | T232 | Hyper | 3.80 | 2.25 | 2.45 | 2.18 | 2.23 |
| MAGED2 | S191 | Hyper | 7.34 | 2.10 | 2.48 | 2.37 | 2.49 |
| MAGED2 | S194 | Hyper | 7.33 | 2.34 | 2.20 | 2.27 | 2.23 |
| MDC1 | S498 | Hyper | 9.18 | 2.31 | 2.34 | 2.40 | 2.26 |
| PHF14 | T287 | Hyper | 7.21 | 2.04 | 2.88 | 1.75 | 2.95 |
| SETD1A | S464 | Hyper | 6.22 | 2.35 | 3.80 | 5.79 | 2.29 |
| KMT2B | S2288 | Hyper | 4.70 | 1.91 | 5.71 | 2.91 | 2.01 |
| KMT2B | T2083 | Hyper | 5.28 | 1.82 | 2.08 | 2.14 | 1.90 |
| PDAP1 | S63 | Hyper | 2.60 | 1.75 | 2.70 | 2.71 | 2.06 |
| SPEN | S1380 | Hyper | 2.88 | 2.01 | 2.27 | 3.46 | 1.69 |
| SPEN | S1918 | Hyper | 6.97 | 2.07 | 4.13 | 4.11 | 1.72 |
| SPEN | S727 | Hyper | 6.09 | 2.11 | 2.71 | 1.93 | 1.74 |
| SPEN | S736 | Hyper | 10.04 | 1.83 | 2.18 | 2.45 | 3.14 |
| CEP170B | S918 | Hyper | 3.66 | 1.73 | 2.13 | 2.06 | 1.60 |
| PDS5B | S1177 | Hyper | 4.60 | 1.99 | 1.74 | 3.24 | 1.66 |
| PDS5B | S1182 | Hyper | 4.94 | 1.89 | 1.88 | 4.68 | 1.66 |

| | | | | | | | |
|---|---|---|---|---|---|---|---|
| ZNF609 | S576 | Hyper | 8.27 | 1.65 | 2.22 | 2.55 | 1.69 |
| TBC1D9B | S432 | Hyper | 3.25 | 2.06 | 1.91 | 5.57 | 2.52 |
| RRP1B | S736 | Hyper | 2.09 | 2.21 | 1.74 | 2.25 | 2.19 |
| SIPA1L3 | S100 | Hyper | 4.24 | 2.00 | 2.81 | 2.07 | 2.23 |
| RCOR1 | S260 | Hyper | 6.09 | 2.07 | 2.87 | 2.37 | 2.32 |
| RCOR1 | S460 | Hyper | 19.72 | 1.88 | 3.10 | 19.86 | 1.93 |
| ZC3H4 | S1275 | Hyper | 7.11 | 1.91 | 2.44 | 2.83 | 1.82 |
| PRRC2C | S924 | Hyper | 15.11 | 1.97 | 4.25 | 2.51 | 2.33 |
| FKBP15 | S1195 | Hyper | 9.62 | 1.73 | 2.91 | 1.81 | 2.24 |
| USP24 | S1943 | Hyper | 12.03 | 2.62 | 3.07 | 2.84 | 1.83 |
| LARP1 | S440 | Hyper | 4.03 | 2.07 | 3.02 | 1.90 | 2.11 |
| LARP1 | S471 | Hyper | 4.30 | 2.34 | 2.15 | 2.44 | 1.87 |
| LARP1 | S554 | Hyper | 4.08 | 1.94 | 2.83 | 2.73 | 2.25 |
| LARP1 | T449 | Hyper | 3.75 | 1.84 | 2.95 | 2.63 | 1.86 |
| SCRIB | S1306 | Hyper | 5.01 | 1.74 | 2.20 | 2.67 | 2.09 |
| SCRIB | S1309 | Hyper | 5.59 | 1.78 | 2.20 | 2.67 | 2.09 |
| NIPBL | S318 | Hyper | 8.22 | 1.89 | 2.11 | 2.04 | 1.60 |
| C2CD2 | S435 | Hyper | 3.34 | 1.83 | 3.26 | 2.64 | 6.80 |
| C2CD2 | S441 | Hyper | 5.31 | 2.69 | 2.90 | 2.49 | 1.97 |
| IBTK | S1045 | Hyper | 7.97 | 1.76 | 2.99 | 3.09 | 1.61 |
| GORASP2 | T415 | Hyper | 8.19 | 1.86 | 3.15 | 1.78 | 2.04 |
| ANKRD17 | S2043 | Hyper | 3.16 | 2.22 | 2.02 | 2.36 | 1.97 |
| ANKRD17 | S2044 | Hyper | 4.26 | 2.03 | 1.94 | 2.16 | 1.96 |
| ANKRD17 | S2046 | Hyper | 4.04 | 2.42 | 1.94 | 2.30 | 1.84 |
| GPATCH4 | S135 | Hyper | 12.30 | 3.10 | 5.43 | 3.50 | 3.02 |
| NOC2L | S49 | Hyper | 11.96 | 3.57 | 3.23 | 2.60 | 2.12 |
| RSL1D1 | S427 | Hyper | 21.10 | 5.77 | 6.22 | 7.17 | 4.34 |
| EIF5B | S113 | Hyper | 5.29 | 2.33 | 2.65 | 4.77 | 2.38 |
| EIF5B | S1168 | Hyper | 4.79 | 1.98 | 3.32 | 2.29 | 2.30 |
| TRIM33 | S862 | Hyper | 6.02 | 2.54 | 3.04 | 2.33 | 1.77 |
| NOP58 | T508 | Hyper | 13.08 | 2.52 | 4.13 | 7.54 | 2.44 |
| NOP58 | T510 | Hyper | 13.80 | 3.29 | 4.09 | 8.56 | 3.31 |
| UTP18 | S121 | Hyper | 7.30 | 3.29 | 3.41 | 3.24 | 2.80 |
| WBP11 | S361 | Hyper | 5.77 | 1.95 | 4.04 | 1.77 | 2.08 |
| WBP11 | S364 | Hyper | 5.67 | 1.95 | 4.34 | 1.87 | 2.85 |
| NUP98 | T1053 | Hyper | 4.73 | 2.21 | 2.35 | 11.53 | 1.79 |
| NUP98 | T529 | Hyper | 4.34 | 2.67 | 2.65 | 2.52 | 2.16 |
| SRRM2 | S1083 | Hyper | 7.02 | 2.11 | 2.66 | 2.72 | 1.87 |
| SRRM2 | S1132 | Hyper | 14.53 | 2.49 | 3.40 | 2.57 | 1.97 |
| SRRM2 | S1320 | Hyper | 8.62 | 2.47 | 2.70 | 2.95 | 1.99 |
| SRRM2 | S1326 | Hyper | 11.32 | 2.43 | 2.76 | 2.87 | 2.01 |
| SRRM2 | S1329 | Hyper | 11.32 | 2.44 | 3.25 | 2.87 | 2.41 |
| SRRM2 | S1424 | Hyper | 15.09 | 2.53 | 2.17 | 2.57 | 2.26 |

| | | | | | | | |
|---|---|---|---|---|---|---|---|
| SRRM2 | S1462 | Hyper | 5.64 | 2.01 | 2.75 | 2.97 | 2.40 |
| SRRM2 | S1857 | Hyper | 14.60 | 2.41 | 3.23 | 2.99 | 1.98 |
| SRRM2 | S1925 | Hyper | 43.54 | 2.30 | 2.54 | 3.37 | 3.95 |
| SRRM2 | S1948 | Hyper | 43.54 | 2.30 | 2.54 | 3.37 | 3.95 |
| SRRM2 | S1960 | Hyper | 43.54 | 2.30 | 2.54 | 3.37 | 3.95 |
| SRRM2 | S2121 | Hyper | 4.79 | 1.93 | 2.76 | 2.89 | 2.71 |
| SRRM2 | S2132 | Hyper | 8.16 | 1.90 | 2.72 | 2.66 | 1.65 |
| SRRM2 | S2398 | Hyper | 7.67 | 2.13 | 2.82 | 2.07 | 1.77 |
| SRRM2 | S2449 | Hyper | 4.09 | 2.08 | 2.33 | 2.34 | 1.85 |
| SRRM2 | S2688 | Hyper | 13.74 | 1.82 | 3.15 | 2.31 | 1.82 |
| SRRM2 | S297 | Hyper | 5.66 | 1.96 | 2.94 | 2.05 | 2.62 |
| SRRM2 | S322 | Hyper | 9.03 | 2.55 | 2.83 | 83.62 | 2.50 |
| SRRM2 | S323 | Hyper | 12.22 | 2.47 | 2.83 | 84.61 | 4.15 |
| SRRM2 | S353 | Hyper | 11.12 | 2.37 | 2.87 | 6.68 | 2.24 |
| SRRM2 | S440 | Hyper | 2.93 | 2.62 | 2.58 | 1.66 | 3.73 |
| SRRM2 | S510 | Hyper | 12.90 | 2.64 | 3.12 | 4.55 | 1.80 |
| SRRM2 | S778 | Hyper | 8.76 | 2.41 | 2.37 | 2.04 | 1.76 |
| SRRM2 | S783 | Hyper | 8.76 | 2.05 | 2.24 | 2.30 | 2.28 |
| SRRM2 | S838 | Hyper | 2.55 | 3.38 | 2.44 | 17.46 | 4.00 |
| SRRM2 | S864 | Hyper | 13.08 | 1.96 | 2.65 | 2.12 | 1.88 |
| SRRM2 | S875 | Hyper | 10.15 | 2.08 | 3.16 | 1.65 | 2.01 |
| SRRM2 | S876 | Hyper | 8.61 | 2.08 | 3.11 | 6.84 | 2.01 |
| SRRM2 | S970 | Hyper | 11.38 | 2.23 | 2.85 | 4.35 | 2.09 |
| SRRM2 | S972 | Hyper | 9.10 | 2.22 | 2.85 | 3.56 | 2.21 |
| SRRM2 | S973 | Hyper | 9.10 | 2.11 | 2.39 | 4.16 | 2.22 |
| SRRM2 | S974 | Hyper | 11.38 | 2.02 | 2.64 | 4.19 | 2.17 |
| SRRM2 | S994 | Hyper | 18.21 | 2.01 | 2.76 | 2.42 | 1.78 |
| SRRM2 | T1003 | Hyper | 9.16 | 2.14 | 2.77 | 2.34 | 2.02 |
| SRRM2 | T1492 | Hyper | 7.05 | 2.12 | 2.46 | 2.71 | 1.64 |
| SRRM2 | T2034 | Hyper | 5.69 | 1.98 | 3.33 | 2.90 | 1.72 |
| SRRM2 | T2104 | Hyper | 8.05 | 2.42 | 2.59 | 2.57 | 1.84 |
| SRRM2 | T866 | Hyper | 9.54 | 1.96 | 3.10 | 4.35 | 2.83 |
| SRRM2 | T983 | Hyper | 11.44 | 2.45 | 2.76 | 3.79 | 2.07 |
| CENPF | S3079 | Hyper | 5.09 | 2.08 | 5.84 | 2.83 | 1.89 |
| HACD3 | S114 | Hyper | 4.80 | 2.32 | 2.08 | 1.84 | 1.94 |
| RSF1 | S1282 | Hyper | 12.93 | 1.87 | 2.87 | 6.26 | 3.39 |
| RSF1 | S1310 | Hyper | 10.80 | 1.73 | 3.02 | 4.43 | 1.91 |
| RSF1 | S1370 | Hyper | 6.38 | 1.90 | 1.73 | 2.73 | 2.07 |
| RSF1 | S1375 | Hyper | 6.66 | 1.92 | 4.88 | 2.23 | 1.97 |
| RSF1 | S397 | Hyper | 9.70 | 2.59 | 3.42 | 4.56 | 2.14 |
| RSF1 | T1371 | Hyper | 6.16 | 1.86 | 1.73 | 2.76 | 3.06 |
| RSF1 | T628 | Hyper | 5.49 | 2.06 | 1.95 | 8.15 | 1.72 |
| KDM3B | S314 | Hyper | 59.91 | 1.96 | 2.21 | 2.49 | 2.03 |

| | | | | | | | |
|---|---|---|---|---|---|---|---|
| RNPC3 | S108 | Hyper | 6.58 | 2.31 | 2.55 | 3.32 | 1.71 |
| VPS13C | S3598 | Hyper | 5.39 | 2.21 | 3.15 | 1.97 | 2.57 |
| CC2D1A | S455 | Hyper | 6.62 | 1.70 | 2.25 | 1.84 | 1.77 |
| ANKHD1 | S1598 | Hyper | 6.54 | 2.14 | 3.09 | 3.63 | 1.91 |
| ALKBH5 | S69 | Hyper | 5.48 | 1.78 | 2.66 | 4.13 | 1.81 |
| TBC1D22B | S132 | Hyper | 8.94 | 1.76 | 3.36 | 3.11 | 3.21 |
| CHD7 | T2567 | Hyper | 4.03 | 2.25 | 2.36 | 1.75 | 2.84 |
| PHIP | S1283 | Hyper | 2.07 | 2.59 | 3.03 | 3.16 | 5.40 |
| PHIP | S1783 | Hyper | 4.95 | 2.04 | 2.05 | 2.35 | 2.07 |
| IWS1 | S422 | Hyper | 10.07 | 2.39 | 5.18 | 1.82 | 4.63 |
| IWS1 | S513 | Hyper | 13.05 | 2.72 | 2.78 | 3.96 | 2.39 |
| KIF26B | S977 | Hyper | 13.59 | 1.98 | 4.27 | 2.68 | 1.96 |
| ASXL2 | S395 | Hyper | 8.41 | 2.14 | 2.78 | 2.01 | 1.70 |
| ASXL2 | S834 | Hyper | 5.84 | 1.79 | 2.17 | 2.22 | 2.07 |
| ZC3H15 | S381 | Hyper | 13.70 | 2.58 | 2.62 | 2.36 | 1.82 |
| TERF2IP | S154 | Hyper | 6.87 | 2.36 | 2.48 | 2.21 | 2.03 |
| SASH3 | S38 | Hyper | 4.04 | 2.20 | 2.21 | 2.25 | 2.68 |
| EML4 | S134 | Hyper | 9.37 | 2.46 | 4.62 | 3.73 | 2.62 |
| EML4 | S146 | Hyper | 6.70 | 1.76 | 2.84 | 3.46 | 2.48 |
| EML4 | T897 | Hyper | 5.87 | 1.57 | 1.78 | 4.18 | 1.95 |
| EML4 | T899 | Hyper | 5.47 | 1.62 | 2.03 | 4.18 | 1.95 |
| KIAA1217 | S1551 | Hyper | 4.97 | 2.42 | 3.05 | 1.90 | 2.05 |
| KIAA1217 | S361 | Hyper | 2.16 | 1.73 | 1.74 | 3.60 | 1.94 |
| KIAA1217 | S365 | Hyper | 4.39 | 1.87 | 1.91 | 3.33 | 1.80 |
| MEPCE | S254 | Hyper | 8.01 | 3.31 | 4.08 | 6.30 | 2.52 |
| MEPCE | S60 | Hyper | 5.40 | 2.16 | 2.80 | 2.75 | 2.09 |
| ZC3HAV1 | S275 | Hyper | 3.18 | 1.75 | 2.13 | 1.72 | 1.79 |
| ZC3HAV1 | S280 | Hyper | 6.28 | 2.10 | 2.19 | 2.70 | 1.76 |
| ZC3HAV1 | S346 | Hyper | 6.57 | 2.12 | 3.00 | 7.41 | 1.62 |
| ZC3HAV1 | S631 | Hyper | 3.51 | 1.57 | 1.73 | 1.95 | 1.59 |
| OTUD7B | S471 | Hyper | 7.25 | 1.75 | 2.66 | 2.53 | 5.37 |
| UTP3 | S368 | Hyper | 3.45 | 2.06 | 2.99 | 1.66 | 2.05 |
| CBX8 | S256 | Hyper | 10.08 | 1.68 | 3.00 | 8.45 | 2.28 |
| GATAD2B | S122 | Hyper | 5.98 | 2.72 | 2.08 | 2.69 | 5.04 |
| GATAD2B | T120 | Hyper | 4.35 | 2.00 | 2.06 | 3.16 | 5.04 |
| PRR12 | S1381 | Hyper | 3.79 | 1.91 | 2.38 | 2.42 | 1.62 |
| NUFIP2 | S214 | Hyper | 8.51 | 2.16 | 5.31 | 1.74 | 4.39 |
| NUFIP2 | S572 | Hyper | 6.15 | 2.16 | 2.96 | 2.80 | 3.68 |
| NUFIP2 | T87 | Hyper | 21.31 | 2.73 | 8.91 | 2.24 | 7.33 |
| CAMSAP3 | S334 | Hyper | 11.79 | 2.50 | 3.14 | 2.85 | 3.81 |
| CAMSAP3 | S814 | Hyper | 13.41 | 2.46 | 3.30 | 3.26 | 2.63 |
| PLEKHA4 | S575 | Hyper | 2.94 | 2.07 | 1.96 | 4.42 | 1.69 |
| ALS2 | S1335 | Hyper | 5.10 | 1.76 | 2.00 | 1.67 | 1.82 |

| | | | | | | | |
|---|---|---|---|---|---|---|---|
| NCOA5 | S29 | Hyper | 3.15 | 4.13 | 2.74 | 1.91 | 1.94 |
| NCOA5 | S34 | Hyper | 1.98 | 2.83 | 2.42 | 1.84 | 1.81 |
| NDUFV3 | S158 | Hyper | 5.22 | 1.88 | 2.29 | 1.76 | 1.87 |
| PPP1R11 | S77 | Hyper | 8.31 | 1.90 | 4.15 | 2.05 | 1.57 |
| PGM5 | S122 | Hypo | 0.47 | 0.23 | 0.52 | 0.18 | 0.32 |
| UBE2O | S896 | Hyper | 5.01 | 1.86 | 1.81 | 2.57 | 2.63 |
| CHTF18 | S871 | Hyper | 9.66 | 2.42 | 3.71 | 2.12 | 5.09 |
| GOLPH3 | S36 | Hyper | 14.04 | 1.94 | 3.95 | 2.09 | 2.15 |
| IFIH1 | S301 | Hyper | 16.92 | 2.20 | 2.56 | 4.33 | 2.82 |
| NCAPG | S674 | Hyper | 14.70 | 3.10 | 6.95 | 3.82 | 3.84 |
| NCAPG | S975 | Hyper | 8.04 | 2.67 | 6.02 | 2.55 | 4.06 |
| DNAJC1 | S480 | Hyper | 2.90 | 1.88 | 3.53 | 3.09 | 1.96 |
| NSD1 | S486 | Hyper | 5.77 | 1.84 | 2.86 | 2.65 | 2.09 |
| SUDS3 | S53 | Hyper | 8.14 | 2.09 | 2.91 | 2.82 | 2.23 |
| SUDS3 | T49 | Hyper | 6.97 | 2.31 | 2.29 | 2.38 | 2.28 |
| ANAPC1 | S688 | Hyper | 3.10 | 2.07 | 3.87 | 2.44 | 1.63 |
| NUCKS1 | S181 | Hyper | 2.26 | 1.93 | 9.39 | 3.00 | 2.60 |
| NUCKS1 | S58 | Hyper | 2.46 | 1.84 | 1.90 | 2.76 | 1.65 |
| NUCKS1 | S61 | Hyper | 3.06 | 1.95 | 1.78 | 2.71 | 1.77 |
| NUCKS1 | S73 | Hyper | 5.04 | 2.35 | 2.29 | 2.31 | 2.32 |
| NUCKS1 | T179 | Hyper | 3.06 | 2.01 | 4.59 | 1.93 | 2.29 |
| ACBD3 | S43 | Hyper | 4.53 | 2.23 | 2.61 | 2.49 | 2.25 |
| PLEKHG2 | S1261 | Hyper | 3.01 | 1.98 | 1.93 | 2.52 | 1.66 |
| CBFB | S173 | Hyper | 7.26 | 2.46 | 2.94 | 2.35 | 2.41 |
| KRI1 | S634 | Hyper | 9.00 | 1.98 | 2.13 | 2.56 | 1.86 |
| MARCKSL1 | S120 | Hyper | 8.49 | 1.68 | 2.01 | 4.29 | 3.67 |
| MARCKSL1 | T122 | Hyper | 9.27 | 2.93 | 2.01 | 5.50 | 3.67 |
| RSRC2 | S27 | Hyper | 12.49 | 1.97 | 2.11 | 1.76 | 2.15 |
| RSRC2 | S32 | Hyper | 8.32 | 1.97 | 1.91 | 2.08 | 1.93 |
| DDX50 | S41 | Hyper | 11.43 | 2.78 | 2.29 | 2.75 | 1.76 |
| C2orf49 | S189 | Hyper | 4.99 | 2.15 | 2.46 | 2.04 | 1.68 |
| CCDC86 | S102 | Hyper | 7.90 | 1.83 | 2.33 | 2.74 | 5.17 |
| CCDC86 | S110 | Hyper | 3.91 | 2.62 | 2.63 | 3.30 | 8.33 |
| CCDC86 | S113 | Hyper | 6.77 | 2.45 | 2.52 | 3.05 | 2.01 |
| DSC2 | S873 | Hyper | 3.51 | 1.54 | 3.97 | 4.16 | 2.30 |
| NKAP | S149 | Hyper | 10.81 | 2.28 | 3.96 | 2.12 | 1.84 |
| NKAP | S157 | Hyper | 9.55 | 2.20 | 4.50 | 2.92 | 2.01 |
| NKAP | T161 | Hyper | 9.55 | 2.36 | 4.50 | 2.92 | 2.01 |
| RNF219 | S210 | Hyper | 22.08 | 4.84 | 6.29 | 6.00 | 3.15 |
| NUP210 | T1844 | Hyper | 9.52 | 2.71 | 3.64 | 4.63 | 2.30 |
| TANC2 | S400 | Hyper | 11.51 | 1.97 | 2.91 | 2.92 | 1.76 |
| TTYH3 | S496 | Hyper | 5.48 | 2.20 | 3.76 | 2.88 | 2.09 |
| ARPC5L | S64 | Hyper | 3.94 | 2.06 | 2.56 | 2.04 | 1.60 |

| | | | | | | | |
|---|---|---|---|---|---|---|---|
| EPPK1 | S2508 | Hyper | 9.43 | 1.97 | 2.43 | 2.30 | 4.58 |
| EPPK1 | S3044 | Hyper | 9.43 | 1.97 | 2.43 | 2.30 | 4.58 |
| EPPK1 | S3575 | Hyper | 9.43 | 1.97 | 2.43 | 2.30 | 4.58 |
| EPPK1 | S4109 | Hyper | 9.43 | 1.97 | 2.43 | 2.30 | 4.58 |
| HUWE1 | S3757 | Hyper | 3.67 | 1.76 | 2.06 | 2.19 | 1.68 |
| HUWE1 | T3924 | Hyper | 4.81 | 1.86 | 2.19 | 4306.11 | 1.98 |
| L3MBTL2 | S683 | Hyper | 6.93 | 1.91 | 2.62 | 1.80 | 2.20 |
| HNRNPU | S59 | Hyper | 5.85 | 1.87 | 2.81 | 3.05 | 1.88 |
| NSRP1 | S254 | Hyper | 8.03 | 2.13 | 3.40 | 6.88 | 3.05 |
| PCNX3 | S1955 | Hyper | 4.95 | 1.69 | 2.39 | 2.24 | 1.74 |
| BAZ1B | S160 | Hyper | 19.80 | 3.15 | 2.11 | 6.11 | 2.48 |
| BAZ1B | S947 | Hyper | 11.68 | 1.84 | 2.00 | 2.13 | 1.68 |
| BUD13 | S163 | Hyper | 5.44 | 1.97 | 1.73 | 1.96 | 1.86 |
| LMNB2 | S407 | Hyper | 1.75 | 2.36 | 2.65 | 5.34 | 1.74 |
| SYAP1 | T248 | Hyper | 3.68 | 1.78 | 2.83 | 2.55 | 2.00 |
| CEP89 | S44 | Hyper | 6.41 | 1.73 | 2.96 | 1.97 | 2.11 |
| PRPF38A | S193 | Hyper | 4.74 | 1.83 | 2.28 | 6.60 | 3.80 |
| PRPF38A | S194 | Hyper | 4.74 | 1.83 | 2.28 | 6.60 | 2.74 |
| CASP2 | S340 | Hyper | 8.82 | 2.89 | 2.10 | 3.74 | 2.04 |
| PURB | S101 | Hyper | 9.66 | 2.13 | 2.87 | 2.66 | 1.90 |
| PML | S403 | Hyper | 3.34 | 2.69 | 2.55 | 3.34 | 1.72 |
| PML | S479 | Hyper | 3.39 | 1.90 | 2.13 | 2.15 | 1.97 |
| PML | S482 | Hyper | 3.02 | 2.15 | 2.10 | 3.40 | 1.97 |
| PML | T409 | Hyper | 7.73 | 2.66 | 2.55 | 4.59 | 1.72 |
| GPRIN1 | S615 | Hyper | 4.82 | 1.76 | 5.18 | 2.50 | 3.26 |
| RBM33 | S205 | Hyper | 5.52 | 2.50 | 2.83 | 4.16 | 1.62 |
| RBM33 | S765 | Hyper | 10.30 | 2.57 | 2.70 | 3.97 | 1.88 |
| GIT2 | S394 | Hyper | 4.66 | 3.15 | 2.93 | 5.44 | 1.90 |
| MYO18A | S2041 | Hyper | 4.98 | 1.61 | 2.10 | 1.70 | 1.95 |
| MYO18A | S2043 | Hyper | 4.35 | 1.78 | 2.02 | 2.50 | 1.64 |
| GLCCI1 | S145 | Hyper | 10.94 | 1.69 | 2.29 | 1.73 | 2.05 |
| GLCCI1 | S148 | Hyper | 3.06 | 1.58 | 2.67 | 2.62 | 2.13 |
| SON | S1076 | Hyper | 3.47 | 2.21 | 2.84 | 2.02 | 2.18 |
| SYNM | S777 | Hypo | 0.55 | 0.18 | 0.46 | 0.25 | 0.28 |
| SRFBP1 | S203 | Hyper | 12.43 | 3.04 | 3.71 | 5.01 | 2.76 |
| PROSER2 | T17 | Hyper | 5.99 | 2.27 | 2.23 | 1.95 | 17.27 |
| UBN2 | S13 | Hyper | 7.86 | 2.32 | 1.99 | 3.67 | 2.01 |
| UBN2 | S584 | Hyper | 20.14 | 2.69 | 3.23 | 4.12 | 1.79 |
| DIP2B | S146 | Hyper | 4.62 | 1.74 | 2.52 | 2.07 | 2.17 |
| DDX51 | S83 | Hyper | 8.05 | 3.08 | 3.04 | 2.61 | 1.70 |
| RETREG3 | S313 | Hyper | 3.54 | 1.95 | 2.35 | 3.24 | 1.90 |
| RETREG3 | S320 | Hyper | 3.80 | 1.86 | 2.39 | 3.18 | 2.06 |
| STT3B | S498 | Hyper | 7.91 | 2.31 | 2.32 | 3.68 | 2.17 |

| | | | | | | | |
|---|---|---|---|---|---|---|---|
| STT3B | S499 | Hyper | 4.59 | 2.17 | 2.14 | 3.11 | 1.96 |
| CRTC2 | S613 | Hyper | 5.21 | 1.73 | 2.20 | 2.48 | 2.20 |
| FAM83H | S516 | Hyper | 7.89 | 1.87 | 3.93 | 1.75 | 1.90 |
| FAM83H | S523 | Hyper | 7.45 | 2.10 | 3.81 | 2.04 | 1.91 |
| FAM83H | S892 | Hyper | 7.32 | 1.78 | 2.96 | 1.91 | 3.61 |
| FAM83H | S914 | Hyper | 8.05 | 1.73 | 2.41 | 1.87 | 2.13 |
| CCDC137 | S19 | Hyper | 6.00 | 2.87 | 2.25 | 3.16 | 1.95 |
| PLEC | S4613 | Hyper | 2.86 | 1.66 | 3.12 | 5.39 | 1.95 |
| PLEC | Y4615 | Hyper | 2.52 | 1.83 | 2.67 | 5.35 | 1.95 |
| PLEC | S4476 | Hyper | 2.86 | 1.66 | 3.12 | 5.39 | 1.95 |
| PLEC | Y4478 | Hyper | 2.52 | 1.83 | 2.67 | 5.35 | 1.95 |
| RBM12B | S280 | Hyper | 8.41 | 2.28 | 2.50 | 2.20 | 3.61 |
| RBM12B | S710 | Hyper | 11.16 | 1.93 | 2.18 | 2.57 | 1.73 |
| RBM12B | S718 | Hyper | 11.91 | 2.08 | 2.32 | 2.25 | 1.73 |
| RBM12B | S839 | Hyper | 4.54 | 2.19 | 2.15 | 2.10 | 1.73 |
| BLOC1S3 | S65 | Hyper | 4.23 | 1.72 | 3.11 | 1.95 | 1.79 |

Table S3: Proteins enriched in the cell cycle pathway

| **Gene symbol** | **Uniprot ID** | **ENSEMBLE ID** |
|---|---|---|
| AAAS | Q9NRG9 | |
| AHCTF1 | Q8WYP5 | |
| ANAPC1 | Q9H1A4 | |
| ANAPC2 | Q9UJX6 | |
| CDC20 | Q12834 | |
| CDCA8 | Q53HL2 | |
| CDK1 | P06493 | ENSG00000170312 |
| CENPF | P49454 | ENSG00000117724 |
| CEP152 | O94986 | |
| CLIP1 | P30622 | |
| CLSPN | Q9HAW4 | |
| DIDO1 | Q9BTC0-1 | |
| GOLGA2 | Q08379 | |
| GORASP2 | Q9H8Y8 | |
| HDAC1 | Q13547 | |
| HSP90AA1 | P07900 | |
| HSP90AB1 | P08238 | |
| INCENP | Q9NQS7 | |
| LIG1 | P18858 | |
| LMNB1 | P20700 | |
| MCM2 | P33993, P49736 | |
| MCM4 | P33991 | |

| | | |
|---|---|---|
| MCM6 | Q14566 | |
| MDC1 | Q14676 | |
| NCAPG | Q9BPX3 | |
| NIPBL | Q6KC79 | |
| NUDC | Q9Y266 | |
| NUP210 | Q8TEM1 | |
| NUP35 | Q8NFH5 | |
| NUP88 | Q99567 | |
| NUP98 | P52948-3, P52948-4, P52948-5 | |
| ORC2 | Q13416 | |
| PDS5A | Q29RF7 | |
| PDS5B | Q9NTI5 | |
| PHF8 | Q9UPP1-3, Q9UPP1-1, Q9UPP1-2 | |
| RANBP2 | P49792 | |
| RANGAP1 | P46060 | |
| RB1 | P06400 | |
| RBBP8 | Q99708 | |
| RSF1 | Q96T23 | |
| SET | Q01105 | |
| TERF2IP | Q9NYB0 | |
| TOP2A | P11388 | ENSG00000131747 |
| TP53BP1 | Q12888 | |
| TPR | P12270 | |
| TUBA1C | P68363, Q9BQE3 | |
| WEE1 | P30291, Q99640 | |
| WRAP53 | Q9BUR4 | |

Table S4: Kinase-substrate links from KSEA analysis

| **Kinase.Gene** | **Substrate.Gene** | **Substrate.Mod** | **Source** | **log2FC** |
|---|---|---|---|---|
| ADRBK1 | CAV1 | S37 | NetworKIN | -2.23897 |
| ADRBK1 | GIT2 | S394 | NetworKIN | 1.65423 |
| ADRBK1 | HDAC1 | S421 | NetworKIN | 1.4713 |
| ADRBK2 | HSP90AA1 | S385 | NetworKIN | 1.11314 |
| AKT1 | HSP90AB1 | S226 | NetworKIN | 1.50592 |
| AKT1 | CARHSP1 | S30 | NetworKIN | 1.16146 |
| AKT1 | CARHSP1 | S32 | NetworKIN | 1.131 |
| AKT1 | SLC9A1 | S605 | NetworKIN | 1.26777 |
| AKT1 | CRTC2 | S613 | NetworKIN | 1.14025 |
| AKT1 | DNMT1 | S143 | PhosphoSitePlus | 1.47309 |
| AKT2 | CRTC2 | S613 | NetworKIN | 1.14025 |

| | | | | |
|---|---|---|---|---|
| ARAF | PBK | S59 | NetworKIN | 1.90205 |
| ATM | TP53BP1 | S1073 | NetworKIN | 1.93093 |
| ATM | TP53BP1 | S836 | NetworKIN | 1.1972 |
| ATM | PNKP | S114 | PhosphoSitePlus | 1.02181 |
| ATR | TP53BP1 | S1073 | NetworKIN | 1.93093 |
| ATR | TP53BP1 | S836 | NetworKIN | 1.1972 |
| AURKA | TOP2A | S1106 | NetworKIN | 2.10765 |
| AURKA | MKI67 | S1131 | NetworKIN | 1.79269 |
| AURKA | TOP2A | S1247 | NetworKIN | 2.44481 |
| AURKA | TOP2A | S1393 | NetworKIN | 2.02465 |
| AURKA | SKA3 | S155 | NetworKIN | 1.18512 |
| AURKA | CDCA8 | S219 | NetworKIN | 1.78881 |
| AURKA | MKI67 | S2223 | NetworKIN | 1.8256 |
| AURKA | INCENP | S263 | NetworKIN | 1.16519 |
| AURKA | ANAPC2 | S314 | NetworKIN | 1.48343 |
| AURKA | MKI67 | S357 | NetworKIN | 1.69444 |
| AURKA | MKI67 | S374 | NetworKIN | 2.27461 |
| AURKA | CDC20 | S41 | NetworKIN | 1.57018 |
| AURKA | TACC3 | S434 | NetworKIN | 1.01613 |
| AURKA | PBK | S59 | NetworKIN | 1.90205 |
| AURKA | NCAPG | S674 | NetworKIN | 1.94288 |
| AURKA | ANAPC1 | S688 | NetworKIN | 1.28971 |
| AURKA | NCAPG | S975 | NetworKIN | 2.02109 |
| AURKA | MKI67 | T1923 | NetworKIN | 1.4364 |
| AURKB | RACGAP1 | S203 | NetworKIN | 2.17411 |
| AURKB | ANAPC1 | S688 | NetworKIN | 1.28971 |
| AURKB | NCAPG | S975 | NetworKIN | 2.02109 |
| AURKB | CDCA8 | S219 | PhosphoSitePlus | 1.78881 |
| AURKB | MKI67 | S374 | PhosphoSitePlus | 2.27461 |
| AURKC | INCENP | S263 | NetworKIN | 1.16519 |
| BMX | CAV1 | Y42 | NetworKIN | -1.49196 |
| BTK | CAV1 | Y42 | NetworKIN | -1.49196 |
| BUB1 | CDC20 | S41 | PhosphoSitePlus | 1.57018 |
| CAMK2A | KRT18 | S30 | NetworKIN | 1.78629 |
| CAMK4 | SRFBP1 | S203 | NetworKIN | 1.89092 |
| CAMK4 | CRTC2 | S613 | NetworKIN | 1.14025 |
| CDC42BPA | MYO18A | S2041 | NetworKIN | 0.96562 |
| CDC42BPA | MYO18A | S2043 | NetworKIN | 1.01456 |
| CDC42BPB | MYO18A | S2041 | NetworKIN | 0.96562 |
| CDC42BPB | MYO18A | S2043 | NetworKIN | 1.01456 |
| CDC7 | MCM2 | S139 | PhosphoSitePlus | 1.42615 |
| CDK1 | TP53BP1 | S1033 | NetworKIN | 1.15002 |
| CDK1 | TP53BP1 | S1099 | NetworKIN | 1.42362 |
| CDK1 | TP53BP1 | S1106 | NetworKIN | 1.47492 |
| CDK1 | MKI67 | S1131 | NetworKIN | 1.79269 |

| | | | | |
|---|---|---|---|---|
| CDK1 | NUDC | S139 | NetworKIN | 1.62252 |
| CDK1 | TP53BP1 | S1431 | NetworKIN | 1.15094 |
| CDK1 | SKA3 | S155 | NetworKIN | 1.18512 |
| CDK1 | WEE1 | S165 | NetworKIN | 1.71177 |
| CDK1 | GTF3C2 | S167 | NetworKIN | 1.11175 |
| CDK1 | TP53BP1 | S1683 | NetworKIN | 2.41735 |
| CDK1 | POLR2A | S1850 | NetworKIN | 1.19168 |
| CDK1 | POLR2A | S1917 | NetworKIN | 0.93825 |
| CDK1 | POLR2A | S1931 | NetworKIN | 0.93825 |
| CDK1 | CDCA8 | S219 | NetworKIN | 1.78881 |
| CDK1 | MKI67 | S2223 | NetworKIN | 1.8256 |
| CDK1 | XRCC1 | S241 | NetworKIN | 2.4465 |
| CDK1 | INCENP | S263 | NetworKIN | 1.16519 |
| CDK1 | HSF1 | S307 | NetworKIN | 1.23979 |
| CDK1 | ANAPC2 | S314 | NetworKIN | 1.48343 |
| CDK1 | DES | S32 | NetworKIN | -1.81277 |
| CDK1 | ATAD2 | S342 | NetworKIN | 2.02787 |
| CDK1 | MKI67 | S357 | NetworKIN | 1.69444 |
| CDK1 | CDC20 | S41 | NetworKIN | 1.57018 |
| CDK1 | USP20 | S413 | NetworKIN | 1.0049 |
| CDK1 | ASPM | S425 | NetworKIN | 1.53023 |
| CDK1 | TACC3 | S434 | NetworKIN | 1.01613 |
| CDK1 | RANGAP1 | S442 | NetworKIN | 2.07844 |
| CDK1 | XRCC1 | S447 | NetworKIN | 1.78105 |
| CDK1 | LIG1 | S51 | NetworKIN | 2.22764 |
| CDK1 | CD2AP | S510 | NetworKIN | 1.94251 |
| CDK1 | CD2AP | S514 | NetworKIN | 1.58184 |
| CDK1 | TP53BP1 | S530 | NetworKIN | 1.07132 |
| CDK1 | PBK | S59 | NetworKIN | 1.90205 |
| CDK1 | NCL | S67 | NetworKIN | 2.06734 |
| CDK1 | SET | S7 | NetworKIN | 1.36837 |
| CDK1 | HSPD1 | S70 | NetworKIN | 1.68869 |
| CDK1 | RBM15 | S700 | NetworKIN | 1.53344 |
| CDK1 | RBM15 | S741 | NetworKIN | 1.42711 |
| CDK1 | RANBP2 | S781 | NetworKIN | 1.72538 |
| CDK1 | MSH6 | S830 | NetworKIN | 1.56627 |
| CDK1 | SIN3A | S860 | NetworKIN | 1.27663 |
| CDK1 | DGCR8 | S95 | NetworKIN | 1.09056 |
| CDK1 | NUCKS1 | T179 | NetworKIN | 1.1932 |
| CDK1 | LIG1 | T183 | NetworKIN | 2.07477 |
| CDK1 | NUP210 | T1844 | NetworKIN | 1.86309 |
| CDK1 | MKI67 | T1923 | NetworKIN | 1.4364 |
| CDK1 | RACGAP1 | T342 | NetworKIN | 1.4927 |
| CDK1 | GORASP2 | T415 | NetworKIN | 1.02916 |
| CDK1 | NCL | T76 | NetworKIN | 2.02747 |
| CDK1 | RANBP2 | T799 | NetworKIN | 1.66902 |

| | | | | |
|---|---|---|---|---|
| CDK1 | TOP2A | S1247 | PhosphoSitePlus | 2.44481 |
| CDK1 | RSF1 | S1375 | PhosphoSitePlus | 1.15962 |
| CDK1 | TOP2A | S1393 | PhosphoSitePlus | 2.02465 |
| CDK1 | DNMT1 | S154 | PhosphoSitePlus | 1.9667 |
| CDK1 | TCOF1 | S156 | PhosphoSitePlus | 2.04302 |
| CDK1 | NUCKS1 | S181 | PhosphoSitePlus | 1.37912 |
| CDK1 | ELAVL1 | S202 | PhosphoSitePlus | 1.15523 |
| CDK1 | LIG3 | S210 | PhosphoSitePlus | 1.46939 |
| CDK1 | RB1 | S249 | PhosphoSitePlus | 1.32839 |
| CDK1 | PAICS | S27 | PhosphoSitePlus | 1.62824 |
| CDK1 | SQSTM1 | S272 | PhosphoSitePlus | 1.31807 |
| CDK1 | HMGA1 | S36 | PhosphoSitePlus | 1.28941 |
| CDK1 | OGFR | S378 | PhosphoSitePlus | 1.17955 |
| CDK1 | LMNB1 | S393 | PhosphoSitePlus | 1.23714 |
| CDK1 | ANAPC1 | S688 | PhosphoSitePlus | 1.28971 |
| CDK1 | RB1 | S807 | PhosphoSitePlus | 1.60762 |
| CDK1 | RANBP2 | T2153 | PhosphoSitePlus | 1.62718 |
| CDK1 | RB1 | T373 | PhosphoSitePlus | 1.47486 |
| CDK1 | EZH2 | T487 | PhosphoSitePlus | 1.26217 |
| CDK1 | NUP98 | T529 | PhosphoSitePlus | 1.40661 |
| CDK1 | HMGA1 | T53 | PhosphoSitePlus | 1.89968 |
| CDK1 | EIF4EBP1 | T70 | PhosphoSitePlus | 1.16322 |
| CDK1 | SRRM2 | T866 | PhosphoSitePlus | 1.63458 |
| CDK2 | WEE1 | S165 | NetworKIN | 1.71177 |
| CDK2 | ANAPC2 | S314 | NetworKIN | 1.48343 |
| CDK2 | LMNB1 | S393 | NetworKIN | 1.23714 |
| CDK2 | AXIN1 | S486 | NetworKIN | 2.0635 |
| CDK2 | AXIN1 | S493 | NetworKIN | 2.10376 |
| CDK2 | ANAPC1 | S688 | NetworKIN | 1.28971 |
| CDK2 | MCM2 | S139 | PhosphoSitePlus | 1.42615 |
| CDK2 | NUDC | S139 | PhosphoSitePlus | 1.62252 |
| CDK2 | DNMT1 | S154 | PhosphoSitePlus | 1.9667 |
| CDK2 | PHF6 | S154 | PhosphoSitePlus | 1.7175 |
| CDK2 | TPR | S1676 | PhosphoSitePlus | 2.12743 |
| CDK2 | NUCKS1 | S181 | PhosphoSitePlus | 1.37912 |
| CDK2 | POLR2A | S1917 | PhosphoSitePlus | 0.93825 |
| CDK2 | ELAVL1 | S202 | PhosphoSitePlus | 1.15523 |
| CDK2 | ANKRD17 | S2044 | PhosphoSitePlus | 1.01822 |
| CDK2 | LIG3 | S210 | PhosphoSitePlus | 1.46939 |
| CDK2 | SNRNP70 | S226 | PhosphoSitePlus | 1.51062 |
| CDK2 | RB1 | S249 | PhosphoSitePlus | 1.32839 |
| CDK2 | PAICS | S27 | PhosphoSitePlus | 1.62824 |
| CDK2 | MKI67 | S357 | PhosphoSitePlus | 1.69444 |
| CDK2 | HMGA1 | S36 | PhosphoSitePlus | 1.28941 |
| CDK2 | RPL12 | S38 | PhosphoSitePlus | 1.98349 |
| CDK2 | LIG1 | S51 | PhosphoSitePlus | 2.22764 |

| | | | | |
|---|---|---|---|---|
| CDK2 | PPP1R35 | S52 | PhosphoSitePlus | 0.91985 |
| CDK2 | NCL | S67 | PhosphoSitePlus | 2.06734 |
| CDK2 | RB1 | S807 | PhosphoSitePlus | 1.60762 |
| CDK2 | C17orf49 | S96 | PhosphoSitePlus | 1.56743 |
| CDK2 | MARCKS | T150 | PhosphoSitePlus | 1.05804 |
| CDK2 | ORC2 | T226 | PhosphoSitePlus | 2.00628 |
| CDK2 | RB1 | T373 | PhosphoSitePlus | 1.47486 |
| CDK2 | NCL | T76 | PhosphoSitePlus | 2.02747 |
| CDK3 | RB1 | S807 | PhosphoSitePlus | 1.60762 |
| CDK4 | MCM4 | S120 | NetworKIN | 1.89214 |
| CDK4 | HSP90AA1 | S385 | NetworKIN | 1.11314 |
| CDK4 | MCM6 | S762 | NetworKIN | 1.3484 |
| CDK4 | CLSPN | S83 | NetworKIN | 2.27774 |
| CDK4 | RB1 | S807 | PhosphoSitePlus | 1.60762 |
| CDK4 | RB1 | T373 | PhosphoSitePlus | 1.47486 |
| CDK5 | NUDC | S139 | NetworKIN | 1.62252 |
| CDK5 | KATNA1 | S170 | NetworKIN | 0.97103 |
| CDK5 | ABI1 | S183 | NetworKIN | 0.84985 |
| CDK5 | SRFBP1 | S203 | NetworKIN | 1.89092 |
| CDK5 | TACC3 | S434 | NetworKIN | 1.01613 |
| CDK5 | SET | S7 | NetworKIN | 1.36837 |
| CDK5 | DNMT1 | S154 | PhosphoSitePlus | 1.9667 |
| CDK5 | ELAVL1 | S202 | PhosphoSitePlus | 1.15523 |
| CDK5 | RB1 | S807 | PhosphoSitePlus | 1.60762 |
| CDK6 | HDAC1 | S423 | NetworKIN | 1.5148 |
| CDK6 | RB1 | S807 | PhosphoSitePlus | 1.60762 |
| CDK7 | POLR2A | S1850 | NetworKIN | 1.19168 |
| CDK7 | POLR2A | S1917 | NetworKIN | 0.93825 |
| CDK7 | POLR2A | S1931 | NetworKIN | 0.93825 |
| CDK7 | SSRP1 | S444 | NetworKIN | 1.35103 |
| CDK7 | MCM2 | S139 | PhosphoSitePlus | 1.42615 |
| CDK7 | CDK1 | T161 | PhosphoSitePlus | 1.22206 |
| CDK9 | RB1 | S807 | PhosphoSitePlus | 1.60762 |
| CHEK1 | RACGAP1 | S203 | PhosphoSitePlus | 2.17411 |
| CHEK1 | CCDC6 | S240 | PhosphoSitePlus | 0.93911 |
| CHUK | SQSTM1 | S24 | NetworKIN | 1.0846 |
| CHUK | SQSTM1 | S272 | NetworKIN | 1.31807 |
| CHUK | HSP90AA1 | S385 | NetworKIN | 1.11314 |
| CLK1 | SRRM2 | S1424 | NetworKIN | 1.33784 |
| CLK1 | SRRM2 | S1857 | NetworKIN | 1.5782 |
| CLK1 | SRRM2 | S1925 | NetworKIN | 1.75443 |
| CLK1 | SRRM2 | S1948 | NetworKIN | 1.75443 |
| CLK1 | BCLAF1 | S196 | NetworKIN | 1.52943 |
| CLK1 | SRRM2 | S1960 | NetworKIN | 1.75443 |
| CLK1 | SRRM2 | S2132 | NetworKIN | 1.40875 |
| CLK1 | SNRNP70 | S226 | NetworKIN | 1.51062 |

| | | | | |
|---|---|---|---|---|
| CLK1 | SRRM2 | S297 | NetworKIN | 1.38899 |
| CLK1 | RSRC2 | S32 | NetworKIN | 0.97756 |
| CLK1 | SRRM2 | S323 | NetworKIN | 2.05395 |
| CLK1 | SRRM2 | S353 | NetworKIN | 1.5189 |
| CLK1 | SRRM2 | S510 | NetworKIN | 1.63986 |
| CLK1 | PPIG | S745 | NetworKIN | 1.30784 |
| CLK1 | SRRM2 | S783 | NetworKIN | 1.18805 |
| CLK1 | SRRM2 | S972 | NetworKIN | 1.51052 |
| CLK1 | SRRM2 | T1003 | NetworKIN | 1.22554 |
| CLK1 | SRRM2 | T2034 | NetworKIN | 1.53495 |
| CLK1 | SRRM2 | T2104 | NetworKIN | 1.36049 |
| CLK1 | SRRM2 | T983 | NetworKIN | 1.46639 |
| CLK2 | CLK3 | S224 | NetworKIN | 1.13123 |
| CLK2 | CLK3 | S226 | NetworKIN | 1.08368 |
| CLK2 | TRA2B | S264 | NetworKIN | 1.71092 |
| CLK2 | TRA2B | S266 | NetworKIN | 1.61851 |
| CLK3 | CLK3 | S224 | NetworKIN | 1.13123 |
| CLK3 | CLK3 | S226 | NetworKIN | 1.08368 |
| CSNK1A1 | SSRP1 | S444 | NetworKIN | 1.35103 |
| CSNK1A1 | AXIN1 | S486 | NetworKIN | 2.0635 |
| CSNK1A1 | RANBP2 | S781 | NetworKIN | 1.72538 |
| CSNK1A1 | RANBP2 | T1396 | NetworKIN | 1.59812 |
| CSNK1A1 | RANBP2 | T799 | NetworKIN | 1.66902 |
| CSNK1D | CLIP1 | S204 | NetworKIN | 1.01096 |
| CSNK1D | CLIP2 | S207 | NetworKIN | 1.1388 |
| CSNK1D | BLOC1S3 | S65 | NetworKIN | 0.96296 |
| CSNK1D | CLSPN | S83 | NetworKIN | 2.27774 |
| CSNK1D | DCK | T72 | PhosphoSitePlus | 0.86846 |
| CSNK1E | AXIN1 | S486 | NetworKIN | 2.0635 |
| CSNK1G1 | DIDO1 | S152 | NetworKIN | 1.25872 |
| CSNK1G1 | AXIN1 | S486 | NetworKIN | 2.0635 |
| CSNK1G1 | DIDO1 | S805 | NetworKIN | 1.27503 |
| CSNK1G1 | DIDO1 | S809 | NetworKIN | 0.94361 |
| CSNK1G2 | TOP2A | S1106 | NetworKIN | 2.10765 |
| CSNK1G2 | TOP2A | S1247 | NetworKIN | 2.44481 |
| CSNK1G2 | AXIN1 | S486 | NetworKIN | 2.0635 |
| CSNK2A1 | TP53BP1 | S1073 | NetworKIN | 1.93093 |
| CSNK2A1 | TOP2A | S1106 | NetworKIN | 2.10765 |
| CSNK2A1 | EIF5B | S113 | NetworKIN | 1.40389 |
| CSNK2A1 | SUB1 | S118 | NetworKIN | 0.9392 |
| CSNK2A1 | TOP2A | S1247 | NetworKIN | 2.44481 |
| CSNK2A1 | RSF1 | S1282 | NetworKIN | 1.76087 |
| CSNK2A1 | RSF1 | S1310 | NetworKIN | 1.59464 |
| CSNK2A1 | ATRX | S1352 | NetworKIN | 1.31504 |
| CSNK2A1 | LIG1 | S141 | NetworKIN | 1.05089 |
| CSNK2A1 | SUB1 | S15 | NetworKIN | 1.33994 |

| | | | | |
|---|---|---|---|---|
| CSNK2A1 | SUB1 | S19 | NetworKIN | 1.68987 |
| CSNK2A1 | MYO18A | S2043 | NetworKIN | 1.01456 |
| CSNK2A1 | SPP1 | S219 | NetworKIN | 1.62338 |
| CSNK2A1 | SPP1 | S254 | NetworKIN | 2.28836 |
| CSNK2A1 | SPP1 | S258 | NetworKIN | 2.05269 |
| CSNK2A1 | BRD7 | S279 | NetworKIN | 1.14635 |
| CSNK2A1 | AATF | S321 | NetworKIN | 1.4587 |
| CSNK2A1 | NCL | S34 | NetworKIN | 1.48753 |
| CSNK2A1 | CAV1 | S37 | NetworKIN | -2.23897 |
| CSNK2A1 | HSP90AA1 | S385 | NetworKIN | 1.11314 |
| CSNK2A1 | NCL | S41 | NetworKIN | 1.31122 |
| CSNK2A1 | NCL | S42 | NetworKIN | 1.31122 |
| CSNK2A1 | IWS1 | S422 | NetworKIN | 2.21232 |
| CSNK2A1 | SSRP1 | S444 | NetworKIN | 1.35103 |
| CSNK2A1 | IWS1 | S513 | NetworKIN | 1.47573 |
| CSNK2A1 | SUDS3 | S53 | NetworKIN | 1.49624 |
| CSNK2A1 | PRPF3 | S619 | NetworKIN | 1.34078 |
| CSNK2A1 | PDAP1 | S63 | NetworKIN | 1.37657 |
| CSNK2A1 | KIF1C | S676 | NetworKIN | 1.13621 |
| CSNK2A1 | ST13 | S75 | NetworKIN | 1.32411 |
| CSNK2A1 | CLSPN | S83 | NetworKIN | 2.27774 |
| CSNK2A1 | SUDS3 | T49 | NetworKIN | 1.20743 |
| CSNK2A1 | ABCF1 | S109 | PhosphoSitePlus | 1.29626 |
| CSNK2A1 | MCM2 | S139 | PhosphoSitePlus | 1.42615 |
| CSNK2A1 | HSP90AB1 | S226 | PhosphoSitePlus | 1.50592 |
| CSNK2A1 | HDAC1 | S421 | PhosphoSitePlus | 1.4713 |
| CSNK2A1 | HDAC1 | S423 | PhosphoSitePlus | 1.5148 |
| CSNK2A1 | NCAPG | S975 | PhosphoSitePlus | 2.02109 |
| CSNK2A2 | SPP1 | S219 | NetworKIN | 1.62338 |
| CSNK2A2 | SPP1 | S234 | NetworKIN | 2.04894 |
| CSNK2A2 | SPP1 | S254 | NetworKIN | 2.28836 |
| CSNK2A2 | SPP1 | S258 | NetworKIN | 2.05269 |
| CSNK2A2 | SPP1 | S263 | NetworKIN | 2.5022 |
| CSNK2A2 | SPP1 | S267 | NetworKIN | 2.53247 |
| CSNK2A2 | NCL | S34 | NetworKIN | 1.48753 |
| CSNK2A2 | CAV1 | S37 | NetworKIN | -2.23897 |
| CSNK2A2 | HSP90AA1 | S385 | NetworKIN | 1.11314 |
| CSNK2A2 | NCL | S41 | NetworKIN | 1.31122 |
| CSNK2A2 | NCL | S42 | NetworKIN | 1.31122 |
| CSNK2A2 | HDAC1 | S421 | NetworKIN | 1.4713 |
| CSNK2A2 | HDAC1 | S423 | NetworKIN | 1.5148 |
| CSNK2A2 | SSRP1 | S444 | NetworKIN | 1.35103 |
| CSNK2A2 | KIF1C | S676 | NetworKIN | 1.13621 |
| CSNK2A2 | XRCC1 | T453 | NetworKIN | 1.74251 |
| DAPK1 | RB1 | T373 | NetworKIN | 1.47486 |

| | | | | |
|---|---|---|---|---|
| DYRK2 | CARHSP1 | S30 | PhosphoSitePlus | 1.16146 |
| DYRK2 | CARHSP1 | S32 | PhosphoSitePlus | 1.131 |
| EGFR | CAV1 | Y42 | NetworKIN | -1.49196 |
| EIF2AK2 | IFIH1 | S301 | NetworKIN | 1.49612 |
| EIF2AK2 | HSP90AA1 | S385 | NetworKIN | 1.11314 |
| ERBB2 | CAV1 | Y42 | NetworKIN | -1.49196 |
| FGR | CAV1 | Y42 | NetworKIN | -1.49196 |
| FYN | CAV1 | Y42 | NetworKIN | -1.49196 |
| GRK1 | CAV1 | S37 | NetworKIN | -2.23897 |
| GRK5 | CAV1 | S37 | NetworKIN | -2.23897 |
| GSK3A | HSF1 | S307 | NetworKIN | 1.23979 |
| GSK3A | AXIN1 | S486 | NetworKIN | 2.0635 |
| GSK3A | AXIN1 | S493 | NetworKIN | 2.10376 |
| GSK3A | SEC31A | S799 | NetworKIN | 1.42551 |
| GSK3A | EIF4EBP1 | T70 | NetworKIN | 1.16322 |
| GSK3B | CLIP2 | S207 | NetworKIN | 1.1388 |
| GSK3B | HSF1 | S307 | NetworKIN | 1.23979 |
| GSK3B | ACBD3 | S43 | NetworKIN | 1.31631 |
| GSK3B | ACLY | S481 | NetworKIN | 0.89944 |
| GSK3B | AXIN1 | S486 | NetworKIN | 2.0635 |
| GSK3B | AXIN1 | S493 | NetworKIN | 2.10376 |
| GSK3B | EIF4EBP1 | T70 | NetworKIN | 1.16322 |
| HIPK1 | PML | S403 | NetworKIN | 1.42702 |
| HIPK1 | PML | T409 | NetworKIN | 1.4118 |
| HIPK2 | SUB1 | S118 | NetworKIN | 0.9392 |
| HIPK2 | MCM4 | S120 | NetworKIN | 1.89214 |
| HIPK2 | CAV1 | S37 | NetworKIN | -2.23897 |
| HIPK2 | PML | S403 | NetworKIN | 1.42702 |
| HIPK2 | RANGAP1 | S442 | NetworKIN | 2.07844 |
| HIPK2 | AXIN1 | S486 | NetworKIN | 2.0635 |
| HIPK2 | RANBP2 | S781 | NetworKIN | 1.72538 |
| HIPK2 | RB1 | S807 | NetworKIN | 1.60762 |
| HIPK2 | VPS4A | S95 | NetworKIN | 1.06374 |
| HIPK2 | NCL | T76 | NetworKIN | 2.02747 |
| HIPK2 | HMGA1 | S36 | PhosphoSitePlus | 1.28941 |
| HIPK2 | HMGA1 | T53 | PhosphoSitePlus | 1.89968 |
| IKBKB | SQSTM1 | S24 | NetworKIN | 1.0846 |
| IKBKB | SQSTM1 | S272 | NetworKIN | 1.31807 |
| IKBKB | HSP90AA1 | S385 | NetworKIN | 1.11314 |
| INSR | CAV1 | Y42 | NetworKIN | -1.49196 |

| | | | | |
|---|---|---|---|---|
| KDR | CAV1 | Y42 | NetworKIN | -1.49196 |
| MAP2K1 | EIF4EBP1 | T70 | NetworKIN | 1.16322 |
| MAP3K8 | CDK1 | T161 | PhosphoSitePlus | 1.22206 |
| MAP3K8 | EIF4EBP1 | T70 | PhosphoSitePlus | 1.16322 |
| MAPK1 | MARCKS | S145 | NetworKIN | 1.32334 |
| MAPK1 | TPR | S1676 | NetworKIN | 2.12743 |
| MAPK1 | ETV6 | S22 | NetworKIN | 1.14926 |
| MAPK1 | RB1 | S249 | NetworKIN | 1.32839 |
| MAPK1 | AXIN1 | S486 | NetworKIN | 2.0635 |
| MAPK1 | RB1 | S807 | NetworKIN | 1.60762 |
| MAPK1 | MARCKS | T150 | NetworKIN | 1.05804 |
| MAPK1 | RB1 | T373 | NetworKIN | 1.47486 |
| MAPK1 | HUWE1 | T3924 | NetworKIN | 1.12847 |
| MAPK1 | GORASP2 | T415 | NetworKIN | 1.02916 |
| MAPK1 | DDX20 | T705 | NetworKIN | 1.19313 |
| MAPK1 | RANBP2 | T799 | NetworKIN | 1.66902 |
| MAPK1 | TOP2A | S1247 | PhosphoSitePlus | 2.44481 |
| MAPK1 | TOP2A | S1393 | PhosphoSitePlus | 2.02465 |
| MAPK1 | ABI1 | S183 | PhosphoSitePlus | 0.84985 |
| MAPK1 | PGK1 | S203 | PhosphoSitePlus | 1.67388 |
| MAPK1 | HNRNPK | S284 | PhosphoSitePlus | 1.22544 |
| MAPK1 | CTTN | S418 | PhosphoSitePlus | 1.15428 |
| MAPK1 | EIF4EBP1 | T70 | PhosphoSitePlus | 1.16322 |
| MAPK1 | RBM17 | T71 | PhosphoSitePlus | 1.3646 |
| MAPK11 | PML | S403 | NetworKIN | 1.42702 |
| MAPK11 | PML | T409 | NetworKIN | 1.4118 |
| MAPK12 | DIDO1 | S805 | NetworKIN | 1.27503 |
| MAPK13 | EIF4EBP1 | T70 | NetworKIN | 1.16322 |
| MAPK13 | SQSTM1 | S272 | PhosphoSitePlus | 1.31807 |
| MAPK14 | ETV6 | S22 | PhosphoSitePlus | 1.14926 |
| MAPK3 | PAK4 | S104 | NetworKIN | 0.94463 |
| MAPK3 | TERF2IP | S154 | NetworKIN | 1.23622 |
| MAPK3 | ETV6 | S22 | NetworKIN | 1.14926 |
| MAPK3 | SQSTM1 | S272 | NetworKIN | 1.31807 |
| MAPK3 | DES | S32 | NetworKIN | -1.81277 |
| MAPK3 | AXIN1 | S486 | NetworKIN | 2.0635 |
| MAPK3 | AXIN1 | S493 | NetworKIN | 2.10376 |
| MAPK3 | CD2AP | S510 | NetworKIN | 1.94251 |
| MAPK3 | CD2AP | S514 | NetworKIN | 1.58184 |
| MAPK3 | ANAPC1 | S688 | NetworKIN | 1.28971 |
| MAPK3 | RB1 | S807 | NetworKIN | 1.60762 |
| MAPK3 | CHD4 | T1542 | NetworKIN | 1.11205 |
| MAPK3 | RB1 | T373 | NetworKIN | 1.47486 |
| MAPK3 | HUWE1 | T3924 | NetworKIN | 1.12847 |
| MAPK3 | EIF4EBP1 | T70 | NetworKIN | 1.16322 |

| | | | | |
|---|---|---|---|---|
| MAPK3 | SGTA | T81 | NetworKIN | 1.12051 |
| MAPK3 | TOP2A | S1247 | PhosphoSitePlus | 2.44481 |
| MAPK3 | TOP2A | S1393 | PhosphoSitePlus | 2.02465 |
| MAPK3 | ABI1 | S183 | PhosphoSitePlus | 0.84985 |
| MAPK3 | HNRNPK | S284 | PhosphoSitePlus | 1.22544 |
| MAPK3 | HSF1 | S307 | PhosphoSitePlus | 1.23979 |
| MAPK3 | CTTN | S418 | PhosphoSitePlus | 1.15428 |
| MAPK7 | TERF2IP | S154 | NetworKIN | 1.23622 |
| MAPK7 | PML | S403 | PhosphoSitePlus | 1.42702 |
| MAPK7 | PML | T409 | PhosphoSitePlus | 1.4118 |
| MAPK8 | RB1 | S249 | NetworKIN | 1.32839 |
| MAPK8 | HSF1 | S307 | NetworKIN | 1.23979 |
| MAPK8 | AXIN1 | S486 | NetworKIN | 2.0635 |
| MAPK8 | AXIN1 | S493 | NetworKIN | 2.10376 |
| MAPK8 | RB1 | S807 | NetworKIN | 1.60762 |
| MAPK9 | RB1 | S249 | NetworKIN | 1.32839 |
| MAPK9 | RB1 | S807 | NetworKIN | 1.60762 |
| MARK3 | PKP2 | S155 | NetworKIN | 1.14792 |
| MARK3 | AXIN1 | S493 | NetworKIN | 2.10376 |
| MARK4 | MYO18A | S2041 | NetworKIN | 0.96562 |
| MARK4 | MYO18A | S2043 | NetworKIN | 1.01456 |
| MTOR | SRRM2 | S1326 | PhosphoSitePlus | 1.46519 |
| MTOR | SRRM2 | S1329 | PhosphoSitePlus | 1.51865 |
| MTOR | DAP | S51 | PhosphoSitePlus | 1.52547 |
| MTOR | DNMT1 | S714 | PhosphoSitePlus | 1.4996 |
| MTOR | EIF4EBP1 | T70 | PhosphoSitePlus | 1.16322 |
| NEK1 | VDAC1 | S104 | NetworKIN | 1.7176 |
| NEK2 | TOP2A | S1106 | NetworKIN | 2.10765 |
| NEK2 | MKI67 | S1131 | NetworKIN | 1.79269 |
| NEK2 | TOP2A | S1247 | NetworKIN | 2.44481 |
| NEK2 | TOP2A | S1393 | NetworKIN | 2.02465 |
| NEK2 | TPR | S1676 | NetworKIN | 2.12743 |
| NEK2 | CLIP1 | S204 | NetworKIN | 1.01096 |
| NEK2 | CDCA8 | S219 | NetworKIN | 1.78881 |
| NEK2 | MKI67 | S2223 | NetworKIN | 1.8256 |
| NEK2 | INCENP | S263 | NetworKIN | 1.16519 |
| NEK2 | ANAPC2 | S314 | NetworKIN | 1.48343 |
| NEK2 | MKI67 | S357 | NetworKIN | 1.69444 |
| NEK2 | MKI67 | S374 | NetworKIN | 2.27461 |
| NEK2 | CDC20 | S41 | NetworKIN | 1.57018 |
| NEK2 | ASPM | S425 | NetworKIN | 1.53023 |
| NEK2 | RANGAP1 | S442 | NetworKIN | 2.07844 |
| NEK2 | PBK | S59 | NetworKIN | 1.90205 |
| NEK2 | NCAPG | S674 | NetworKIN | 1.94288 |
| NEK2 | ANAPC1 | S688 | NetworKIN | 1.28971 |
| NEK2 | NCAPG | S975 | NetworKIN | 2.02109 |

| | | | | |
|---|---|---|---|---|
| NEK2 | MKI67 | T1923 | NetworKIN | 1.4364 |
| PAK1 | GIT2 | S394 | NetworKIN | 1.65423 |
| PAK1 | CTTN | S418 | PhosphoSitePlus | 1.15428 |
| PAK2 | MYO18A | S2041 | NetworKIN | 0.96562 |
| PAK3 | GIT2 | S394 | NetworKIN | 1.65423 |
| PAK4 | PAK4 | S104 | NetworKIN | 0.94463 |
| PDK1 | HSP90AA1 | S385 | NetworKIN | 1.11314 |
| PIM1 | CBX3 | S97 | NetworKIN | 1.32785 |
| PKD1 | OSBP | S389 | NetworKIN | 1.32172 |
| PKD1 | SLC9A1 | S605 | NetworKIN | 1.26777 |
| PKD1 | CFL1 | T25 | NetworKIN | 1.26505 |
| PKM | AKT1S1 | S203 | PhosphoSitePlus | 1.08363 |
| PLK1 | TP53BP1 | S1072 | NetworKIN | 1.70646 |
| PLK1 | TP53BP1 | S1073 | NetworKIN | 1.93093 |
| PLK1 | MCM2 | S139 | NetworKIN | 1.42615 |
| PLK1 | TP53BP1 | S1431 | NetworKIN | 1.15094 |
| PLK1 | TP53BP1 | S1623 | NetworKIN | 1.27193 |
| PLK1 | TP53BP1 | S1683 | NetworKIN | 2.41735 |
| PLK1 | CLIP1 | S204 | NetworKIN | 1.01096 |
| PLK1 | CDCA8 | S219 | NetworKIN | 1.78881 |
| PLK1 | TP53BP1 | S530 | NetworKIN | 1.07132 |
| PLK1 | TP53BP1 | S644 | NetworKIN | 1.15098 |
| PLK1 | TP53BP1 | S645 | NetworKIN | 0.95277 |
| PLK1 | CLSPN | S83 | NetworKIN | 2.27774 |
| PLK1 | TP53BP1 | S836 | NetworKIN | 1.1972 |
| PLK1 | TP53BP1 | S839 | NetworKIN | 1.43801 |
| PLK1 | NUDC | T145 | NetworKIN | 1.66862 |
| PLK1 | HNRNPU | S59 | PhosphoSitePlus | 1.4917 |
| PLK1 | ANAPC1 | S688 | PhosphoSitePlus | 1.28971 |
| PLK4 | TOP2A | S1106 | NetworKIN | 2.10765 |
| PLK4 | TOP2A | S1247 | NetworKIN | 2.44481 |
| PLK4 | PBK | S59 | NetworKIN | 1.90205 |
| PLK4 | NCAPG | S674 | NetworKIN | 1.94288 |
| PLK4 | NCAPG | S975 | NetworKIN | 2.02109 |
| PRKAA1 | PRKAB1 | S96 | NetworKIN | 1.17051 |
| PRKAA2 | CRTC2 | S613 | NetworKIN | 1.14025 |
| PRKAA2 | PRKAB1 | S96 | NetworKIN | 1.17051 |
| PRKACB | ITPR3 | S2670 | NetworKIN | 0.95805 |
| PRKACG | ITPR3 | S2670 | NetworKIN | 0.95805 |
| PRKCA | CLIP1 | S204 | NetworKIN | 1.01096 |
| PRKCA | ITPR3 | S2670 | NetworKIN | 0.95805 |
| PRKCA | CAV1 | S37 | NetworKIN | -2.23897 |
| PRKCA | MARCKS | T150 | NetworKIN | 1.05804 |
| PRKCB | PPP1R13L | S134 | NetworKIN | 1.04302 |
| PRKCB | SRRM2 | S1948 | NetworKIN | 1.75443 |
| PRKCB | SRRM2 | S1960 | NetworKIN | 1.75443 |

| | | | | |
|---|---|---|---|---|
| PRKCB | CLIP1 | S204 | NetworKIN | 1.01096 |
| PRKCB | ZC3HAV1 | S280 | NetworKIN | 1.12906 |
| PRKCB | NCOA5 | S34 | NetworKIN | 0.98535 |
| PRKCB | PRKAB1 | S96 | NetworKIN | 1.17051 |
| PRKCB | CARHSP1 | T45 | NetworKIN | 1.41489 |
| PRKCB | EEF2 | T59 | NetworKIN | 1.40194 |
| PRKCD | MARCKS | T150 | NetworKIN | 1.05804 |
| PRKCE | CAV1 | S37 | NetworKIN | -2.23897 |
| PRKCE | MARCKS | T150 | NetworKIN | 1.05804 |
| PRKCG | ITPR3 | S2670 | NetworKIN | 0.95805 |
| PRKCG | MARCKS | T150 | NetworKIN | 1.05804 |
| PRKCI | SQSTM1 | S24 | NetworKIN | 1.0846 |
| PRKCZ | SQSTM1 | S24 | NetworKIN | 1.0846 |
| PRKCZ | MARCKS | T150 | NetworKIN | 1.05804 |
| PRKDC | TP53BP1 | S1033 | NetworKIN | 1.15002 |
| PRKDC | TP53BP1 | S1073 | NetworKIN | 1.93093 |
| PRKDC | TP53BP1 | S1099 | NetworKIN | 1.42362 |
| PRKDC | TP53BP1 | S1106 | NetworKIN | 1.47492 |
| PRKDC | SUB1 | S118 | NetworKIN | 0.9392 |
| PRKDC | MCM4 | S120 | NetworKIN | 1.89214 |
| PRKDC | TP53BP1 | S1431 | NetworKIN | 1.15094 |
| PRKDC | SUB1 | S15 | NetworKIN | 1.33994 |
| PRKDC | TP53BP1 | S1623 | NetworKIN | 1.27193 |
| PRKDC | SUB1 | S19 | NetworKIN | 1.68987 |
| PRKDC | XRCC1 | S241 | NetworKIN | 2.4465 |
| PRKDC | HSF1 | S307 | NetworKIN | 1.23979 |
| PRKDC | XRCC1 | S447 | NetworKIN | 1.78105 |
| PRKDC | TP53BP1 | S530 | NetworKIN | 1.07132 |
| PRKDC | TP53BP1 | S644 | NetworKIN | 1.15098 |
| PRKDC | TP53BP1 | S645 | NetworKIN | 0.95277 |
| PRKDC | TP53BP1 | S836 | NetworKIN | 1.1972 |
| PRKDC | TP53BP1 | S839 | NetworKIN | 1.43801 |
| PRKDC | HNRNPU | S59 | PhosphoSitePlus | 1.4917 |
| PRKG1 | PGK1 | S203 | NetworKIN | 1.67388 |
| PRKG1 | SPP1 | S219 | NetworKIN | 1.62338 |
| PRKG1 | SPP1 | S234 | NetworKIN | 2.04894 |
| PRKG1 | SPP1 | S254 | NetworKIN | 2.28836 |
| PRKG1 | SPP1 | S258 | NetworKIN | 2.05269 |
| PRKG1 | SPP1 | S263 | NetworKIN | 2.5022 |
| PRKG1 | SPP1 | S267 | NetworKIN | 2.53247 |
| PRKG1 | ITPR3 | S2670 | NetworKIN | 0.95805 |
| PRKG1 | BLOC1S3 | S65 | NetworKIN | 0.96296 |
| PRKG2 | ITPR3 | S2670 | NetworKIN | 0.95805 |
| PRKG2 | BLOC1S3 | S65 | NetworKIN | 0.96296 |
| RAF1 | PAK4 | S104 | NetworKIN | 0.94463 |
| RAF1 | VDAC1 | S104 | NetworKIN | 1.7176 |

| | | | | |
|---|---|---|---|---|
| RAF1 | TERF2IP | S154 | NetworKIN | 1.23622 |
| RAF1 | HSP90AB1 | S226 | NetworKIN | 1.50592 |
| RAF1 | RB1 | S249 | NetworKIN | 1.32839 |
| RAF1 | HSP90AA1 | S385 | NetworKIN | 1.11314 |
| RAF1 | PBK | S59 | NetworKIN | 1.90205 |
| RAF1 | RB1 | S807 | NetworKIN | 1.60762 |
| ROCK1 | RTKN | S106 | NetworKIN | 1.35638 |
| ROCK1 | DES | S32 | NetworKIN | -1.81277 |
| ROCK1 | RTKN | S543 | NetworKIN | 1.35739 |
| ROCK1 | SLC9A1 | S605 | NetworKIN | 1.26777 |
| ROCK1 | CFL1 | T25 | NetworKIN | 1.26505 |
| ROCK2 | RTKN | S106 | NetworKIN | 1.35638 |
| RPS6KA2 | YBX1 | S165 | NetworKIN | 1.37003 |
| RPS6KA2 | YBX1 | S174 | NetworKIN | 1.2611 |
| RPS6KA2 | CARHSP1 | S30 | NetworKIN | 1.16146 |
| RPS6KA2 | CARHSP1 | S32 | NetworKIN | 1.131 |
| RPS6KA2 | CARHSP1 | T45 | NetworKIN | 1.41489 |
| RPS6KA2 | EIF4EBP1 | T70 | NetworKIN | 1.16322 |
| SRC | CAV1 | Y42 | NetworKIN | -1.49196 |
| STK11 | SGTA | T81 | NetworKIN | 1.12051 |
| STK39 | SLC12A7 | T973 | PhosphoSitePlus | 0.96572 |
| TGFBR2 | CAV1 | S37 | NetworKIN | -2.23897 |
| TGFBR2 | HSP90AA1 | S385 | NetworKIN | 1.11314 |
| TTK | MKI67 | S374 | NetworKIN | 2.27461 |
| TTK | RSF1 | T1371 | NetworKIN | 1.46563 |
| TTK | NUCKS1 | T179 | NetworKIN | 1.1932 |
| TTK | MKI67 | T1923 | NetworKIN | 1.4364 |
| TTK | RACGAP1 | T342 | NetworKIN | 1.4927 |
| TTK | RSF1 | T628 | NetworKIN | 1.04594 |
| TTK | EIF4EBP1 | T70 | NetworKIN | 1.16322 |

Table S5: Kinase enrichment score from KSEA analysis

| **Kinase.Gene** | **mS** | **Enrichment** | **m** | **z.score** | **p.value** | **FDR** |
|---|---|---|---|---|---|---|
| ADRBK1 | 0.295517 | 0.220374816 | 3 | -3.537 | 0.000202 | 0.001303 |
| ADRBK2 | 1.113138 | 0.830096642 | 1 | -0.44503 | 0.328148 | 0.453172 |
| AKT1 | 1.279915 | 0.954467009 | 6 | -0.29214 | 0.38509 | 0.460769 |
| AKT2 | 1.14025 | 0.850315061 | 1 | -0.39207 | 0.347502 | 0.453172 |
| ARAF | 1.902048 | 1.418408172 | 1 | 1.095946 | 0.136551 | 0.426205 |
| ATM | 1.383314 | 1.031574341 | 3 | 0.143246 | 0.443048 | 0.465652 |
| ATR | 1.564068 | 1.166367507 | 2 | 0.616272 | 0.268857 | 0.453172 |

| | | | | | | |
|---|---|---|---|---|---|---|
| AURKA | 1.720302 | 1.282875442 | 18 | 3.143551 | 0.000835 | 0.004776 |
| AURKB | 1.909665 | 1.424087903 | 5 | 2.483876 | 0.006498 | 0.027887 |
| AURKC | 1.165187 | 0.868911153 | 1 | -0.34336 | 0.365662 | 0.453172 |
| BMX | -1.49196 | -1.112595154 | 1 | -5.53357 | 1.57E-08 | 1.47E-07 |
| BTK | -1.49196 | -1.112595154 | 1 | -5.53357 | 1.57E-08 | 1.47E-07 |
| BUB1 | 1.570181 | 1.170925806 | 1 | 0.44771 | 0.327181 | 0.453172 |
| CAMK2A | 1.786289 | 1.33208301 | 1 | 0.869833 | 0.192196 | 0.453172 |
| CAMK4 | 1.515585 | 1.130212032 | 2 | 0.482342 | 0.314781 | 0.453172 |
| CDC42BPA | 0.990093 | 0.738338841 | 2 | -0.96927 | 0.166206 | 0.450506 |
| CDC42BPB | 0.990093 | 0.738338841 | 2 | -0.96927 | 0.166206 | 0.450506 |
| CDC7 | 1.426154 | 1.063520806 | 1 | 0.166382 | 0.433928 | 0.460769 |
| CDK1 | 1.495789 | 1.115449576 | 73 | 2.583704 | 0.004887 | 0.021886 |
| CDK2 | 1.583902 | 1.181157796 | 31 | 2.641964 | 0.004121 | 0.019295 |
| CDK3 | 1.607621 | 1.198845496 | 1 | 0.520841 | 0.301239 | 0.453172 |
| CDK4 | 1.618984 | 1.207319172 | 6 | 1.330161 | 0.091733 | 0.337445 |
| CDK5 | 1.383153 | 1.03145377 | 9 | 0.247163 | 0.402391 | 0.460769 |
| CDK6 | 1.561213 | 1.164237936 | 2 | 0.608384 | 0.271467 | 0.453172 |
| CDK7 | 1.177903 | 0.878393492 | 6 | -0.78023 | 0.217628 | 0.453172 |
| CDK9 | 1.607621 | 1.198845496 | 1 | 0.520841 | 0.301239 | 0.453172 |
| CHEK1 | 1.556607 | 1.160803042 | 2 | 0.59566 | 0.275701 | 0.453172 |
| CHUK | 1.171936 | 0.873944053 | 3 | -0.57189 | 0.283698 | 0.453172 |
| CLK1 | 1.490059 | 1.111176607 | 20 | 1.302319 | 0.096404 | 0.342399 |
| CLK2 | 1.386084 | 1.033639846 | 4 | 0.176227 | 0.430058 | 0.460769 |
| CLK3 | 1.107453 | 0.825857558 | 2 | -0.64507 | 0.25944 | 0.453172 |
| CSNK1A1 | 1.681411 | 1.253873231 | 5 | 1.486932 | 0.068516 | 0.282288 |
| CSNK1D | 1.251783 | 0.933487813 | 5 | -0.38956 | 0.348431 | 0.453172 |
| CSNK1E | 2.063504 | 1.538809821 | 1 | 1.411317 | 0.079076 | 0.301659 |
| CSNK1G1 | 1.385214 | 1.032990831 | 4 | 0.172827 | 0.431394 | 0.460769 |
| CSNK1G2 | 2.205322 | 1.6445674 | 3 | 2.924274 | 0.001726 | 0.00889 |
| CSNK2A1 | 1.436305 | 1.071090659 | 38 | 1.147872 | 0.125511 | 0.41702 |
| CSNK2A2 | 1.453001 | 1.083541469 | 16 | 0.875288 | 0.190709 | 0.453172 |
| DAPK1 | 1.474865 | 1.099846117 | 1 | 0.261529 | 0.396842 | 0.460769 |
| DYRK2 | 1.14623 | 0.854774581 | 2 | -0.53796 | 0.295304 | 0.453172 |
| EGFR | -1.49196 | -1.112595154 | 1 | -5.53357 | 1.57E-08 | 1.47E-07 |
| EIF2AK2 | 1.304631 | 0.972897977 | 2 | -0.10039 | 0.460016 | 0.473816 |
| ERBB2 | -1.49196 | -1.112595154 | 1 | -5.53357 | 1.57E-08 | 1.47E-07 |
| FGR | -1.49196 | -1.112595154 | 1 | -5.53357 | 1.57E-08 | 1.47E-07 |
| FYN | -1.49196 | -1.112595154 | 1 | -5.53357 | 1.57E-08 | 1.47E-07 |
| GRK1 | -2.23897 | -1.669662118 | 1 | -6.99271 | 1.35E-12 | 6.94E-11 |
| GRK5 | -2.23897 | -1.669662118 | 1 | -6.99271 | 1.35E-12 | 6.94E-11 |
| GSK3A | 1.599159 | 1.192535265 | 5 | 1.127676 | 0.129728 | 0.417563 |
| GSK3B | 1.417834 | 1.057316517 | 7 | 0.397208 | 0.345607 | 0.453172 |
| HIPK1 | 1.41941 | 1.058491699 | 2 | 0.21667 | 0.414233 | 0.460769 |
| HIPK2 | 1.314554 | 0.980297884 | 12 | -0.17877 | 0.429059 | 0.460769 |
| IKBKB | 1.171936 | 0.873944053 | 3 | -0.57189 | 0.283698 | 0.453172 |
| INSR | -1.49196 | -1.112595154 | 1 | -5.53357 | 1.57E-08 | 1.47E-07 |

| | | | | | | |
|---|---|---|---|---|---|---|
| KDR | -1.49196 | -1.112595154 | 1 | -5.53357 | 1.57E-08 | 1.47E-07 |
| MAP2K1 | 1.163223 | 0.867446581 | 1 | -0.3472 | 0.36422 | 0.453172 |
| MAP3K8 | 1.19264 | 0.889383762 | 2 | -0.40975 | 0.340993 | 0.453172 |
| MAPK1 | 1.452649 | 1.083278944 | 20 | 0.975527 | 0.164649 | 0.450506 |
| MAPK11 | 1.41941 | 1.058491699 | 2 | 0.21667 | 0.414233 | 0.460769 |
| MAPK12 | 1.275026 | 0.950820527 | 1 | -0.12882 | 0.448751 | 0.466883 |
| MAPK13 | 1.240646 | 0.925182765 | 2 | -0.27714 | 0.390835 | 0.460769 |
| MAPK14 | 1.149262 | 0.857035206 | 1 | -0.37447 | 0.354027 | 0.453172 |
| MAPK3 | 1.289196 | 0.961387589 | 22 | -0.47438 | 0.317614 | 0.453172 |
| MAPK7 | 1.358346 | 1.012954745 | 3 | 0.058773 | 0.476566 | 0.486003 |
| MAPK8 | 1.668613 | 1.244329149 | 5 | 1.431032 | 0.076211 | 0.301659 |
| MAPK9 | 1.468003 | 1.094729093 | 2 | 0.350903 | 0.36283 | 0.453172 |
| MARK3 | 1.625841 | 1.212432744 | 2 | 0.786911 | 0.215667 | 0.453172 |
| MARK4 | 0.990093 | 0.738338841 | 2 | -0.96927 | 0.166206 | 0.450506 |
| MTOR | 1.434425 | 1.069688685 | 5 | 0.408166 | 0.341576 | 0.453172 |
| NEK1 | 1.717597 | 1.280857859 | 1 | 0.735657 | 0.23097 | 0.453172 |
| NEK2 | 1.775563 | 1.324084589 | 20 | 3.796317 | 7.34E-05 | 0.000504 |
| PAK1 | 1.404252 | 1.04718829 | 2 | 0.174799 | 0.430619 | 0.460769 |
| PAK2 | 0.965625 | 0.720092211 | 1 | -0.73317 | 0.231728 | 0.453172 |
| PAK3 | 1.654225 | 1.23359982 | 1 | 0.611873 | 0.270311 | 0.453172 |
| PAK4 | 0.944628 | 0.704434222 | 1 | -0.77418 | 0.219412 | 0.453172 |
| PDK1 | 1.113138 | 0.830096642 | 1 | -0.44503 | 0.328148 | 0.453172 |
| PIM1 | 1.327853 | 0.990215183 | 1 | -0.02563 | 0.489776 | 0.489776 |
| PKD1 | 1.284846 | 0.95814408 | 3 | -0.18989 | 0.424697 | 0.460769 |
| PKM | 1.083634 | 0.808094401 | 1 | -0.50266 | 0.307601 | 0.453172 |
| PLK1 | 1.484799 | 1.107254352 | 17 | 1.15832 | 0.123367 | 0.41702 |
| PLK4 | 2.083697 | 1.553868127 | 5 | 3.243997 | 0.000589 | 0.003571 |
| PRKAA1 | 1.170506 | 0.872877783 | 1 | -0.33297 | 0.369577 | 0.453172 |
| PRKAA2 | 1.155378 | 0.861596422 | 2 | -0.51269 | 0.304086 | 0.453172 |
| PRKACB | 0.958051 | 0.714444052 | 1 | -0.74796 | 0.227241 | 0.453172 |
| PRKACG | 0.958051 | 0.714444052 | 1 | -0.74796 | 0.227241 | 0.453172 |
| PRKCA | 0.197021 | 0.146923652 | 4 | -4.46897 | 3.93E-06 | 2.89E-05 |
| PRKCB | 1.296065 | 0.96651032 | 9 | -0.26316 | 0.396213 | 0.460769 |
| PRKCD | 1.058043 | 0.789010921 | 1 | -0.55265 | 0.290252 | 0.453172 |
| PRKCE | -0.59047 | -0.440325599 | 2 | -5.33537 | 4.77E-08 | 4.09E-07 |
| PRKCG | 1.008047 | 0.751727486 | 2 | -0.91967 | 0.178872 | 0.453172 |
| PRKCI | 1.084602 | 0.80881657 | 1 | -0.50077 | 0.308266 | 0.453172 |
| PRKCZ | 1.071322 | 0.798913745 | 2 | -0.74488 | 0.228172 | 0.453172 |
| PRKDC | 1.422781 | 1.061005509 | 19 | 0.696522 | 0.243051 | 0.453172 |
| PRKG1 | 1.849214 | 1.379008277 | 9 | 2.978235 | 0.00145 | 0.007858 |
| PRKG2 | 0.960504 | 0.716273083 | 2 | -1.051 | 0.146628 | 0.444197 |
| RAF1 | 1.419444 | 1.058517527 | 8 | 0.433531 | 0.332315 | 0.453172 |
| ROCK1 | 0.686762 | 0.512136923 | 5 | -2.85741 | 0.002136 | 0.010475 |
| ROCK2 | 1.35638 | 1.011489081 | 1 | 0.030094 | 0.487996 | 0.489776 |
| RPS6KA2 | 1.250284 | 0.932369881 | 6 | -0.43392 | 0.332175 | 0.453172 |
| SRC | -1.49196 | -1.112595154 | 1 | -5.53357 | 1.57E-08 | 1.47E-07 |

| | | | | | | |
|---|---|---|---|---|---|---|
| STK11 | 1.120514 | 0.835596824 | 1 | -0.43063 | 0.333371 | 0.453172 |
| STK39 | 0.965721 | 0.720163914 | 1 | -0.73298 | 0.231785 | 0.453172 |
| TGFBR2 | -0.56292 | -0.419782738 | 2 | -5.25928 | 7.23E-08 | 5.73E-07 |
| TTK | 1.438814 | 1.072961703 | 7 | 0.50563 | 0.306558 | 0.453172 |
